# Characterizing selective pressures on the pathway for de novo biosynthesis of pyrimidines in yeast

**DOI:** 10.1186/s12862-015-0515-x

**Published:** 2015-10-28

**Authors:** Russell A. Hermansen, Brian K. Mannakee, Wolfgang Knecht, David A. Liberles, Ryan N. Gutenkunst

**Affiliations:** Department of Molecular Biology, University of Wyoming, Laramie, WY 82071 USA; Department of Biology and Center for Computational Genetics and Genomics, Temple University, Philadelphia, PA 19122 USA; Division of Epidemiology and Biostatistics, Mel and Enid Zuckerman College of Public Health, University of Arizona, Tucson, AZ 85721 USA; Department of Biology and Lund Protein Production Platform, Lund University, 22362 Lund, Sweden; Department of Molecular and Cellular Biology, University of Arizona, Tucson, AZ 85721 USA

**Keywords:** Evolutionary systems biology, Metabolic pathway evolution, Phylogenetics, Kinetic model, Enzyme evolution, Substitution rate

## Abstract

**Background:**

Selection on proteins is typically measured with the assumption that each protein acts independently. However, selection more likely acts at higher levels of biological organization, requiring an integrative view of protein function. Here, we built a kinetic model for *de novo* pyrimidine biosynthesis in the yeast *Saccharomyces cerevisiae* to relate pathway function to selective pressures on individual protein-encoding genes.

**Results:**

Gene families across yeast were constructed for each member of the pathway and the ratio of nonsynonymous to synonymous nucleotide substitution rates (dN/dS) was estimated for each enzyme from *S. cerevisiae* and closely related species. We found a positive relationship between the influence that each enzyme has on pathway function and its selective constraint.

**Conclusions:**

We expect this trend to be locally present for enzymes that have pathway control, but over longer evolutionary timescales we expect that mutation-selection balance may change the enzymes that have pathway control.

**Electronic supplementary material:**

The online version of this article (doi:10.1186/s12862-015-0515-x) contains supplementary material, which is available to authorized users.

## Background

Predicting functional change in proteins based upon either mutations segregating in a population or substitutions fixed between populations is a fundamental goal in modern computational genomics. Many approaches for making such predictions rely upon tests for selection, with (for example) the view that inter-specific functional changes may have been driven to fixation by positive directional selection. A common test for this type of problem is dN/dS, the ratio of nonsynonymous to synonymous nucleotide substitution rates. In using this measure (or other measures of selection) to predict functional shifts, one is making the assumption that each protein-coding gene functions independently. However, it is well known that proteins function as part of larger pathways or macromolecular structures and it is through these combined functions that selection actually acts (see [1]). An example of this that will be applied here in metabolism is the contribution of each enzyme to steady-state pathway flux, as described by a kinetic model (characterizing the kinetics of each step of a pathway based upon underlying enzymatic rate parameters).

One prediction from coupling between enzymes in a pathway is that when a pathway is under negative selection (or other types of selection), that the selective pressure on amino acid change in an individual protein will relate to the sensitivity of pathway function to perturbation of each individual enzyme based upon amino acid changes. This model is based upon an expectation that enzyme function will account for a sizeable fraction of selective constraint on a protein. Several previous studies have examined the relationship between evolutionary rate and pathway flux, including examination of the effects of network topology [2–5], although a picture linked to underlying evolutionary processes has not yet fully emerged.

Other factors have also been discussed as contributors to amino acid substitution. Folding stability and specificity independent of function contribute to amino acid substitution, and this drives faster substitution on the surface than the core, with the surface area to volume ratio of a protein’s fold providing some potential signal for a difference in relative rates [6, 7]. Negative aspects of function (selective pressures to prevent spurious interactions or activities) are also a potential contributor to relative substitution rates and selective pressures, with an expected link between surface hydrophobicity and rates of evolution [8]. This second mechanism is linked to an observation that expression level is an important driver of selective constraint. Highly expressed proteins are thought to be under stronger constraint to avoid spurious interactions that become more probable at increased concentration [9–11]. Lastly, it has been proposed that selection for translational fidelity is a major contributor to relative substitution rate [12]. Ultimately, all of these factors will interplay in determining which amino acid substitutions are fixed and the relative rate of fixation. This makes naïve measures of selection on proteins (like dN/dS) potentially poor predictors of functional change.

We are interested in examining the contribution to functional selective constraint as well as amino acid substitution more generally, from protein function defined at the pathway level. The ultimate aim of this study and research trajectory is to understand the evolution of protein functions in a cellular and organismal context (and to develop tools to do so). Here, we develop a model pathway to study, that of pyrimidine biosynthesis in yeast, with a particular emphasis on *S. cerevisiae*.

**Fig. 1:**
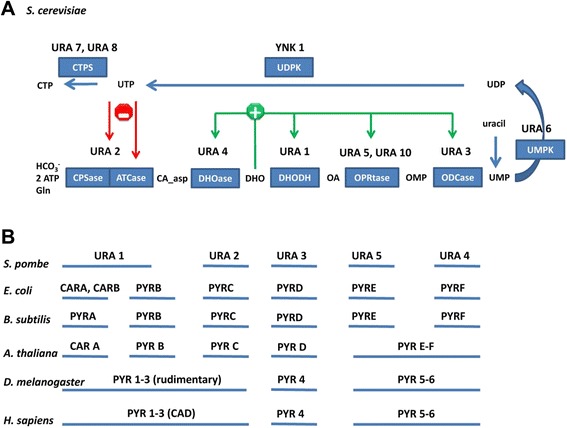
**a.** Schematic presentation of the *de novo* pyrimidine biosynthesis and its feedback regulation in *S. cerevisiae*. Red arrows show downregulation of enzymatic activity and gene expression, respectively. Green arrows show upregulation of gene expression. Enzymatic activities are represented in the boxes, with *S. cerevisiae* gene names above them: Carbamoyl-phosphate synthetase (CPSase, URA2) and aspartate transcarbamylase (ATCase, URA2), dihydroorotase (DHOase, URA4), dihydroorotate dehydrogenase (DHODH, URA1), orotate phosphoribosyltransferase (OPRtase, URA5 and URA10), orotidine-5’-phosphate decarboxylase (ODCase, URA3), uridylate kinase (UMPK, URA6), nucleoside diphosphate kinase (UDPK, YNK1), CTP synthase (CTPS, URA7 and URA8). The free intermediates of the pathway are N-carbamoyl-aspartate (CA_asp), dihydroorotate (DHO), orotate (OA), orotidylate (OMP). **b.** Comparison of the *de novo* pyrimidine biosynthesis in selected organisms. Figure modified after [13, 14, 18]

The six enzymatic steps involved in pyrimidine biosynthesis occur nearly universally in all organisms (Fig. 1). However organization into multifunctional enzymes as well as subcellular localization and regulation change with evolution [13–16]. The end product of the pathway, uridine monophosphate (UMP), is further phosphorylated to uridine diphosphate (UDP) and uridine triphosphate (UTP) that can be further converted to cytidine triphosphate (CTP), thereby providing the two pyrimidine building blocks of RNA. At the diphosphate level, they are substrates for ribonucleotide reductase, channeling them into the synthesis of DNA precursors, deoxyribonucleoside triphosphates. *S. cerevisiae* can salvage uracil, e.g. from the surrounding environment, and this salvage pathways enters into a common RNA and DNA precursor synthesis at the UMP level. In *S. cerevisiae* the pathway consists of 6 proteins (Fig. 1). URA2 is a multifunctional enzyme catalyzing the first two enzymatic steps of the pathway, and its activity is negatively regulated in a feedback loop by the RNA precursor UTP, both at the gene expression level as well as at the enzymatic activity level [13, 17]. The organization of the first two activities into one enzyme is also seen in other yeast like *S. pombe* [18]. The only other regulation known in yeast in this pathway is by dihyhdroorotate that positively affects the gene expression of all other proteins of the pathway [13]. The fourth reaction of the pathway is catalyzed in *S. cerevisiae* by a cytosolic enzyme, while in other yeast this step can be catalyzed by a mitochondrial enzyme coupled to the respiratory chain. Yeast like *S. kluyveri* have both isoenzymes [19–21]. In contrast to other species, including other yeasts, two isoenzymes URA5 and URA10 catalyze the 5^th^ reaction of the pathway in *S. cerevisiae* [13]. The third and sixth reactions are catalyzed by single enzymes, URA4 and URA3, respectively [22–24].

In this study, we characterize the phylogenetic history of the enzyme gene families in yeast, including identification of the relative rates of evolution in the clade including *S. cerevisiae*. We also build a kinetic model for the pathway in *S. cerevisiae* by homology to the model from *E. coli*. Lastly, we compare the sensitivity of steady-state pathway flux for each enzyme to the relative level of selective constraint each enzyme is under in an attempt to assess the importance of pathway function in driving enzyme evolution as well as the relationship between local evolutionary constraint and function at a higher level of organization. This involves phrasing an important evolutionary question in two directions, “How well does a more sophisticated model of protein function explain observed evolutionary patterns?” and conversely, “How well do simple evolutionary statistics describing selective constraint (here dN/dS) describe conservation of function or the opportunity for functional shifts in a pathway?”. Ultimately, this modeling framework can be envisioned as leading to the development of new statistical tests for functional shifts in comparative and population genomics.

## Results and Discussion

### Phylogenetic analysis

Phylogenetic trees for each of the URA genes involved in the *de novo* pyrimidine biosynthesis pathway in *S. cerevisiae* S288c were constructed. The constructed phylogenies were then assessed for selective strength using two different tests for dN/dS; the free-ratios model, in which ω is allowed to vary across each branch, and the 2-ratio model, in which ω is constrained for each branch except for the lineage of interest. The 2-ratio model was supported in the YNK1 gene family, but not in any of the other gene families. The free-ratios model was supported in four of the gene families (two of which had no target sequence for kinetic analysis), but the dS ratio for these lineages showed extremely low dS such that the dN/dS estimates for these lineages were unreliable. Hence the dN/dS ratio from the 1-ratio model was compared with the kinetic parameter sensitivity for each of the gene families. It should be noted as a caveat that there are potential errors in estimation for maximum likelihood point estimates.

In each family, proteins with high dN/dS ratios may reflect selective pressures that alter enzymatic function, either quantitatively (corresponding to changes in kinetic parameter values) or qualitatively (corresponding to changes in the structure of the differential equation kinetic model). Of course, as discussed in the introduction, selection may also be acting on attributes of protein sequence that are independent of these types of function. Further, our phylogenetic analysis was used to pinpoint well-supported candidate gene duplication and lateral transfer events. We hypothesize that these events may have functionally changed either kinetic parameter values or the structure of the kinetic model. Unfortunately, because kinetic data is currently unavailable outside of *S. cerevisiae*, we cannot presently test these hypothesises.

#### URA1

The phylogenetic analysis of the URA1 gene family revealed that the URA1 gene in *S. cerevisiae* S288c is evolving under negative selection with a dN/dS value of 0.31 (Table 1). However, it is under more relaxed selection than in several other strains of *S. cerevisiae*. The S288c strain of yeast is a laboratory strain that has undergone strong selective pressure for rapid growth in a nutrient rich environment [25]. Other strains of yeast have additionally been placed under strong selective pressures based upon the different industrial uses to which they are applied. Within the URA1 gene family we found that both the laboratory S288c strain and the VL3 wine production strain were under weaker selective constraint than both the wine strain AWRI796 and the human pathogen strain YJM789. The VL3 strain was under slight negative selection with a dN/dS ratio of 0.90 (Additional file 1: Figure S1), which is close to the dN/dS of 1 that would indicate the absence of selective pressure on the gene. This could be indicative of the domestication process that each of these strains has evolved under. Since the laboratory strain has been domesticated in a nutrient rich environment (and protected from competition), there is potentially less selective pressure on the pyrimidine biosynthesis pathway than there would be for other strains. As noted by Borneman et al. [26], six sequenced strains of yeast from similar and different industrial and laboratory backgrounds showed substantial genomic differences, even for yeast strains from the same industrial setting. These differences include differences in chromosome copy numbers, ORFs, and novel genes, which may explain why each of these strains appears to be evolving nearly independently of each other and therefore may be under different selective constraints.

**Table 1:** Results of the phylogenetic analysis

Gene Family	GI#	dS tree length	dN/dS (Free-ratio)	dS branch (Free-ratio)	dN/dS (2-ratio)	dS branch length (2-ratio)	dN/dS (1-ratio)	*P*-value (Free vs 2-Ratio)	*P*-value (Free vs 1-Ratio)	*P*-value (2 vs 1-Ratio)
URA1	6322633	2.38	0.31	<0.01	0.29	<0.01	***0.05***	<0.01	<0.01	0.26
URA2	--	3.05	--	--	--	--	***0.03***	--	--	--
ATC_ase_	--	2.58	--	--	--	--	***0.02***	--	--	--
CPS_ase_	--	0.78	--	--	--	--	***0.01***	--	--	--
URA3	398364267	1.63	<0.01	<0.01	2.00	<0.01	***0.04***	0.09	0.12	>0.99
URA4	--	0.93	--	--	--	--	***0.14***	--	--	--
URA5	6323530	0.09	<0.01	<0.01	<0.01	<0.01	***0.14***	**<0.01**	**<0.01**	>0.99
URA6	398364671	1.05	0.40	0.01	0.39	0.01	***0.11***	0.69	0.69	0.37
URA7	6319432	1.02	1.71	<0.01	2.00	<0.01	***0.05***	**<0.01**	**<0.01**	>0.99
URA10	6323927	1.54	<0.01	0.01	<0.01	<0.01	***0.08***	0.08	0.13	0.99
YNK1	6322783	1.30	<0.01	<0.01	<0.01	<0.01	***0.05***	0.10	**0.05**	**0.005**

dN/dS values according to different nested approaches in PAML [38] were estimated. dN/dS values in bold were compared with kinetic parameter sensitivities. Underlined dN/dS values showed low dS and were considered to be poorly estimated (dS < 0.001)

The divergence of *S. cerevisiae* URA1 might also be explained by the way *S. cerevisiae* catalyzes the fourth reaction in the *de novo* pyrimidine biosynthesis pathway compared to other yeast. In other yeast this reaction is performed by a mitochondrial enzyme coupled to the respiratory chain, while in *S. cerevisiae* it is performed in the cytosol.

**Fig. 2:**
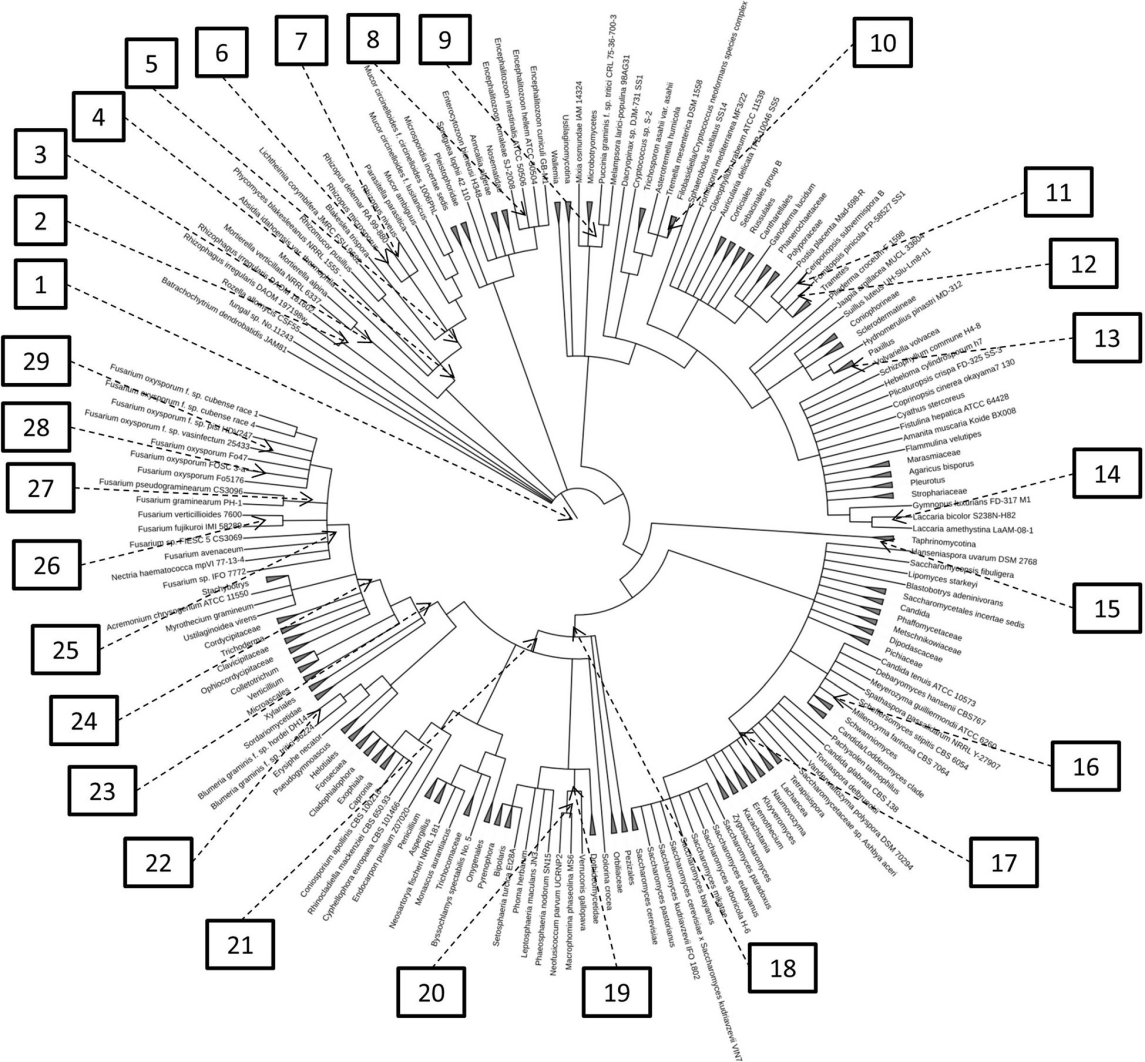
Gene evolution within the fungal species tree. Shown is the NCBI fungal species tree annotated with inferred gene duplication and lateral transfer events following gene tree/species tree reconciliation. Duplication events marked as paralog/xenolog were ambiguous and not obviously differentiable between being a gene duplication event and a lateral transfer event. The numbered branches within the figure indicate the following duplication and lateral transfer events: 1) Branch: Fungi [URA1 – Paralog(2), URA6 – Paralog(3), URA6 – Paralog/Xenolog(3), YNK1 – Paralog, YNK1 – Paralog/Xenolog], 2) Branch: *Rhizophagus irregulare* [URA5/10 – Paralog], 3) Branch: Mortierella [URA7 – Paralog, YNK1 – Paralog], 4) Branch: Mucorales [URA7 – Paralog], 5) Mucorineae [URA2 – Paralog, URA7 – Paralog], 6) Branch: *Rhizopus microsporus* [URA1 – Paralog, URA7 – Paralog, YNK1 – Paralog], 7) Branch: *Rhizopus delemar* [URA2 – Paralog], 8) Branch: *Encephalitozoon intestinalis* [URA7 – Paralog], 9) Branch: Pucciniales [URA6 – Paralog], 10) Branch: Filobasidiella/*Cryptococcus neoformans* species complex [URA5/10 – Paralog(5)], 11) Branch: Ceriporiopsis [URA1 – Paralog], 12) Branch: *Fomitopsis pinicola* [URA7 – Paralog], 13) Branch: *Paxillus involutus* [YNK1 – Paralog], 14) Branch: *Laccaria bicolor* [URA5/10 – Paralog], 15) Branch: Taphrinomycotina [YNK1 – Xenolog], 16) Branch: *Millerozyma farinosa* [YNK1 – Paralog], 17) Branch: Saccharomycetacea [URA1 – Xenolog, URA5/10 – Paralog(2), URA7 – Paralog], 18) Branch: Pezizomycotina [URA3 – Paralog], 19) Pleosporineae [URA7 – Paralog], 20) Branch: Botryosphaeriaceae [URA3 – Paralog], 21) Branch: Leotiomyceta [URA3 – Paralog/Xenolog, URA7 – Paralog, URA7 – Paralog/Xenolog], 22) Branch: *Blumeria graminis f. sp. Hordei* DH14 [URA5/10 – Paralog], 23) Branch: Sordariomycetes [URA7 – Paralog], 24) Branch: Hypocreales [URA7 – Paralog], 25) Branch: Fusarium [URA7 – Paralog(3)], 26) Branch: *Fusarium verticillioides* [URA7 – Paralog], 27) Branch: *Fusarium sambucinum species complex* [URA7 – Paralog], 28) Branch: *Fusarium oxysporum* FOSC 3-a [URA7 – Paralog], 29) Branch: *Fusarium oxysporum* f. sp. Vasinfectum 25433 [URA7 – Paralog]. An expandable pdf version of Fig. 2 is also found within the supplementary materials

An examination of the entire URA1 gene family additionally showed four different high confidence duplication events and one potential horizontal gene transfer (HGT) event after being reconciled against the fungal species tree using a soft parsimony-based approach (Fig. 2; Additional file 1: Figure S12).

#### URA2

The kinetic parameters for this gene were drawn from the strain MD171-1C, which derives from the wild-type strain FL-100 [27, 28]. This strain was not present in the phylogenetic analysis. Although a phylogenetic tree for the URA2 gene family was constructed, we were unable to determine relative rates of dN/dS for the MD171-1C strain and thus used the 1-ratio estimate of dN/dS of 0.03 (Table 1) to infer the dN/dS ratio for the lineage of MD171-1C. Additionally, we examined the CPSase and ATCase domains from the URA2 gene (strain S288c) individually to determine the relative rates of dN/dS for these domains, because they carry out distinct enzymatic reactions. The 1-ratio estimate of dN/dS was again used for both of the domains, with the CPSase domain having an estimate of 0.01 (Additional file 1: Figure S11) and the ATCase domain having an estimate of 0.02 (Additional file 1: Figure S10). This conformed to the hypothesis that the two domains are under stronger negative selection than the complete protein, with the CPSase domain under slightly stronger negative constraint.

From the URA2 gene family, it was possible to additionally assess two different high confidence duplication events which occurred in the early diverging fungal lineages. The two paralogs mapped to the Mucorineae lineage and a lineage specific duplication on the lineage leading to *Rhizopus delemar* (Fig. 2; Additional file 1: Figure S13).

#### URA3

The dN/dS analysis of the URA3 gene tree did not show support for either the free-ratio or 2-ratio analysis, therefore the 1-ratio dN/dS value of 0.04 was used as the rate of evolution for the S288c lineage (Table 1). To test how this dN/dS value varied based upon small perturbations within the tree, a subtree of the URA3 gene family was pruned and examined. The 1-ratio analysis showed that the subtree had a dN/dS value of 0.80, which is highly more relaxed than the initial estimate, indicating this gene tree may be highly influenced by small changes within the tree topology. Additionally it was noted that a putative URA1 gene from *Pneumocystis jirovecii* grouped within the *S. cerevisiae* genes. This could potentially be an artifactual relationship and the result of phylogenetic error, as these two species are distantly related. Alternatively, this could be a signal associated with a lateral transfer event.

The gene tree/species tree reconciliation of URA3 showed three different duplications that might have occurred throughout the gene family and one possible HGT. Two of the duplications mapped to older lineages within the fungal species tree, Peziziomycetina and Sordariomycetes. The third duplication was along the Botryosphaeriaceae lineage. A potential xenolog/paralog was additionally found on the Leotiomycetes lineage but was consistent with both gene duplication and lateral transfer as hypotheses for the origin (Fig. 2; Additional file 1: Figure S14).

#### URA4

The kinetic parameters for the URA4 reaction were estimated and not based on a specific strain of yeast. Therefore we were unable to calculate a dN/dS ratio on a specific lineage to estimate the relative rate of evolution. We were able to estimate the dN/dS ratio for the 1-ratio test in which ω is constant throughout the tree. This resulted in a dN/dS estimate of 0.14 (Table 1). Interestingly, similar to the URA1 gene tree, two *S. cerevisiae* strains showed elevated dN/dS ratios compared to the rest of the tree in the free-ratios analysis (which was supported at below the 1 % level). The two strains were the FostersB strain with a dN/dS ratio of 1.04 and an unspecified *S. cerevisiae* strain (GI: 4765) with a dN/dS of 0.99 (Additional file 1: Figure S4). The FostersB strain is used in industry in the production of ale, and furthermore has been shown to contain the most heterozygous SNPs compared to all other *S. cerevisiae* strains [29]. This strain is known to contain at least 36 ORFs not present in the S288c laboratory strain, and it appears to be evolving differently than the laboratory and wine strains. Therefore, it is plausible that URA4 could be under different selective constraints in this strain than in other strains. The other *S. cerevisiae* strain was unknown, and therefore it is unclear why this strain would exhibit an elevated dN/dS ratio compared to the other *S. cerevisiae* strains within the gene family. The URA4 gene family did not show clear signs of gene duplication events (Additional file 1: Figure S15).

#### URA5/URA10

The URA5 and URA10 gene families were highly similar, with the URA10 gene family being the larger of the two (Additional file 1: Figure S16; Additional file 1: Figure S17). These two gene families originated from a gene duplication event and contained two distinct groups of *S. cerevisiae* genes, grouping into the URA5 genes and URA10 genes. The URA5 gene family was supported by the free-ratios model but the lineage for S288c was not possible to estimate accurately due to low dS along the branch. Therefore the 1-ratio estimate of 0.14 was used as the branch estimate of dN/dS for the *S. cerevisiae* S288c lineage (Table 1).

Furthermore, like the URA4 gene tree, the FostersB strain in the URA5 gene family under the free-ratios model showed elevated signals of dN/dS compared to the rest of the tree. The dN/dS for this branch was 0.71 (Additional file 1: Figure S5), indicating highly relaxed selective constraint along this lineage. As suggested above, this may indicate that the FostersB strain is evolving differently from the rest of the *S. cerevisiae* strains.

The URA10 gene family was not supported at the free-ratios level or at the 2-ratio level. Therefore a subtree was assessed for relative rates of evolution. From the 1-ratio model the dN/dS for each branch within the tree was estimated at 0.08, and the dN/dS ratio of the subtree was estimated at 0.06. The dN/dS ratio did not alter much with the perturbation of the tree (as described in the Methods section) and was therefore considered a reasonable indicator that the S288c strain is evolving under strong negative selection.

The URA5 and URA10 gene families showed several different duplication events to have occurred throughout the gene family, with no putative HGT events. Both of these gene families showed numerous duplications within the Filobasidiella lineage and the along *Cryptococcus neoformans* lineage. Additionally there was a lineage-specific duplication within *Blumeria graminis* which resulted in two different copies within the *B. graminis* hordei D14 strain. The duplication analysis also identified a duplication event on the Saccharomycetacea lineage which was putatively responsible for the divergence of the URA5 and URA10 gene families (Fig. 2; Additional file 1: Figure S16; Additional file 1: Figure S17).

#### URA6

The URA6 gene family encodes for a uridine monophosphate kinase [30] and is responsible for catalyzing the seventh step in the *de novo* pyrimidine biosynthesis pathway. The URA6 gene family was not supported for the free-ratio model; therefore the 1-ratio dN/dS estimate of 0.11 was used for the tree (Table 1). This gene family showed two distinct duplication events that occurred along the *S. cerevisiae* lineage, resulting in three different clades containing *S. cerevisiae* genes (Fig. 2; Additional file 1: Figure S18). The first of the three clades was the URA6 gene family and the other two clades encoded an adenylate kinase ADK1 and an adenylate kinase ADK2. Adenylate kinases are important for regulating energy levels within the cell and are responsible for catalyzing the reaction of ATP and AMP to 2 ADP. Only the YJM789 *S. cerevisiae* strain was found in all three of the clades, while the S288c strain showed evidence of a URA6 gene and a single adenylate kinase, ADK2. This suggests that some of the adenylate kinase activity might be lost within some of the *S. cerevisiae* strains.

The URA6 gene family duplication analysis was also able to identify one other potential paralog and three potential paralog/xenologs at the base of the fungal tree. These putative duplications and HGT events lead to several species being placed distantly within the tree from their locations in the fungal species tree (Fig. 2).

#### URA7

The free-ratios analysis for the URA7 gene family, which encodes for a CTP synthetase, was significant at the 0.01 level; however the dS ratio for the S288c lineage was too low to accurately estimate the dN/dS ratio for the branch (dS < 0.001). Therefore the 1-ratio estimate of 0.05 was used for the gene family (Table 1). Within the URA7 gene family there was also an additional clade of URA8 genes, which also putatively encode a CTP synthetase. These proteins were separated from the URA7 genes via a duplication event along the Saccharomyces lineage, suggesting that this duplication was specific to the Saccharomyces clade (Additional file 1: Figure S19).

The URA7 gene family was also the most expansive of the all of the URA gene families studied in this analysis, with 18 putative duplication events and two putative HGT events. A large number of putative duplications were located within the genus Fusarium, with seven duplications occurring within the Fusarium clade (Fig. 2; Additional file 1: Figure S19).

#### YNK1

The YNK1 gene family, which is a nucleoside diphosphate kinase, was the only family to show support for the 2-ratio vs 1-ratio likelihood ratio test. However the 1-ratio model dN/dS estimate of 0.05 (Table 1) was used for the family, because the dS estimate for the *S. cerevisiae* lineage of interest was too low to accurately estimate the dN/dS for the specific lineage (dS < 0.001).

The duplication analysis of the YNK1 gene family revealed one putative xenolog, one ambiguous paralog/xenolog and five additional high confidence gene duplications. The paralog/xenolog was mapped to the origin of the fungal species tree as well as one of the paralogs, while the putative xenolog was found along the Taphrinomycotina lineage. The other duplications were recent lineage specific duplications, spread out throughout the YNK1 gene tree (Fig. 2; Additional file 1: Figure S20).

### Kinetic modeling

Our kinetic model for the yeast pyrimidine biosynthesis pathway was inspired by the Rodriguez et al. [31] model for the pathway in *E. coli*. We optimized the 28 parameters in the kinetic model to reproduce the observed steady-state concentrations of UMP, UDP, and UTP, while minimizing deviation from experimentally measured parameter values (Table 2). We found the optimization to be well-constrained, with only a single global optimum. Over 100 optimizations runs, the coefficients of variation for inferred parameter values were all less than 10 %, with the exception of K_m8_ (~30 %) and g_pyr_ (~80 %). Moreover, these parameter sets all generated highly similar influences (standard deviations all less than 1e-3), and influence rankings (mean rank correlation rho = 0.998). Thus in Table 2 we report only the single parameter set that produced the lowest total cost. This parameter set closely reproduced the observed metabolite concentrations (Table 3), suggesting a good fit between the model and the data.

**Table 2:** Kinetic model parameter values and sensitivites

Parameter	Description	Experimental reference	Initial value	Optimized value	Sensitivity	Enzyme
vmax1	Vmax for carbamoyl synthetase	[48]	5.40 × 10^−1^	3.62	9.68 × 10^−1^	URA2
K_utp_	UTP binding constant	[48]	1.40	1.41	5.49 × 10^−3^	URA2
K_atp_	ATP binding constant	[48]	7.50	1.29	−8.68 × 10^−1^	URA2
K_q_	Km for glutamine	[48]	7.00 × 10^−2^	5.78 × 10^-2^	−9.32 × 10^−2^	URA2
K_bc_	Km for bicarbonate	[48]	8.00	2.37	−5.96 × 10^−1^	URA2
vmax2	Vmax for aspartate	[49]	1.10	2.45	2.00 × 10^−1^	URA2
K_asp_	Km for aspartate	[50]	2.80 × 10^−1^	1.68 × 10^−1^	−1.24 × 10^−1^	URA2
K_m2_	Km for aspartate	[49]	4.00	2.00	−1.77 × 10^−1^	URA2
vmax3	Vmax for dihydroorotase		2.47 × 10^1^	2.87 × 10^1^	1.21 × 10^−4^	URA4
K_m3_	Km for dihydroorotase		7.00 × 10^−1^	1.27	−1.20 × 10^−4^	URA4
vmax4	Vmax for dihydroorotate dehydrogenase	[21]	9.18 × 10^1^	9.18 × 10^1^	1.73 × 10^−5^	URA1
K_m4_	Km for dihydroorotate dehydrogenase	[21]	1.60 × 10^−2^	1.60 × 10^−2^	−1.73 × 10^−5^	URA1
vmax5	Vmax for orotate phosphoribosyl transferase	[51]	5.18 × 10^3^	5.23 × 10^3^	2.10 × 10^−5^	URA5
K_m5_	Km for orotate phosphoribosyl transferase	[51]	1.97 × 10^−2^	1.95 × 10^−2^	−2.10 × 10^−5^	URA5
vmax6	Vmax for OMP decarboxylase	[52]	3.03 × 10^1^	3.50 × 10^1^	4.63 × 10^−2^	URA3
K_m6_	Km for OMP decarboxylase		3.20 × 10^1^	2.03 × 10^1^	−4.62 × 10^−2^	URA3
vmax7	Vmax for nucleoside diphosphate kinase	[53]	6.48	5.83	−6.92 × 10^−5^	YNK1
K_m7_	Km for nucleoside diphosphate kinase	[53]	1.50 × 10^−1^	1.66 × 10^−1^	6.79 × 10^−5^	YNK1
vmax8	Vmax for CTP synthase	[54]	5.40	1.63 × 10^−1^	2.46 × 10^−2^	URA7
K_m8_	Km for CTP synthase	[54]	7.40 × 10^−4^	4.36 × 10^−3^	−5.22 × 10^−3^	URA7
vmax10	Vmax for UMP kinase	[55]	1.14	6.56	−1.02	URA6
K_m10_	Km for UMP kinase	[55]	1.50 × 10^−1^	2.68 × 10^−2^	1.00	URA6
g_pyr_	Pyrimidine utilization rate		4.00 × 10^−1^	1.98 × 10^−1^	1.16 × 10^−4^	
K_Mp_	Km for pyrimidine utilization		5.80	5.49	−1.16 × 10^−4^	
bc	Intracellular bicarbonate conc.	[39]	4.51 × 10^−1^	1.52	5.96 × 10^−1^	
glu	Intracellular glutamine conc.	[39]	4.51 × 10^−1^	5.46 × 10^−1^	9.32 × 10^−2^	
asp	Intracellular aspartate conc.	[39]	5.85 × 10^−2^	9.73 × 10^−2^	1.24 × 10^−1^	
atp	Initial ATP conc.	[39]	2.59 × 10^−2^	1.51 × 10^−1^	8.68 × 10^−1^	

vmax parameters in units of mM/hr. K_m_ parameters and concentrations in units of mM

**Table 3:** Steady-state model metabolite concentrations

Metabolite	Model concentration (mM)	Experimental concentration (mM; [39])
ump	4.2 × 10^−4^	3.7 × 10^−4^
udp	2.9 × 10^−3^	2.9 × 10^−3^
utp	6.7 × 10^−3^	6.7 × 10^−3^
ctp	7.5 × 10^−1^	
cp	2.7 × 10^−1^	
ca	4.7 × 10^−3^	
dho	1.8 × 10^−5^	
oro	2.2 × 10^−6^	
omp	5.8 × 10^−2^	

In general, optimized values were close to initial values, however six parameters (vmax1, vmax2, vmax10, K_m8_, atp, and bc) changed by a factor of two or greater. vmax1 and vmax2 are rate parameters for the first two reactions in the pathway, both of which take place on the combined enzyme URA2. atp and bc are substrate concentrations for the first reaction, and vmax10 is a rate parameter for UMP kinase URA6. These parameters are among the most sensitive in the model, and as such it is not surprising that the optimization procedure adjusts those parameters to best reproduce metabolite concentrations. K_m8_ is the Michealis constant for UTP for the CTP synthase URA7 that converts UTP to CTP. While this parameter is not particularly influential in terms of model behavior, UTP is one of the metabolites whose concentration we constrained in the optimization process, such that parameters sensitive to its concentration might need to be adjusted. This level of deviation is not unexpected when comparing biochemical inference from different experiments [32].

For each parameter we then calculated the sensitivity of the steady-state UMP concentration to changes in that parameter. These varied over many orders of magnitude (Table 2). The largest values were for parameters involved in the CPSase activity of URA2 and the UMP kinase URA6, suggesting that these two enzymes act as control points for flux through the pathway.

### Relation between pathway kinetics and enzyme evolution

**Fig. 3:**
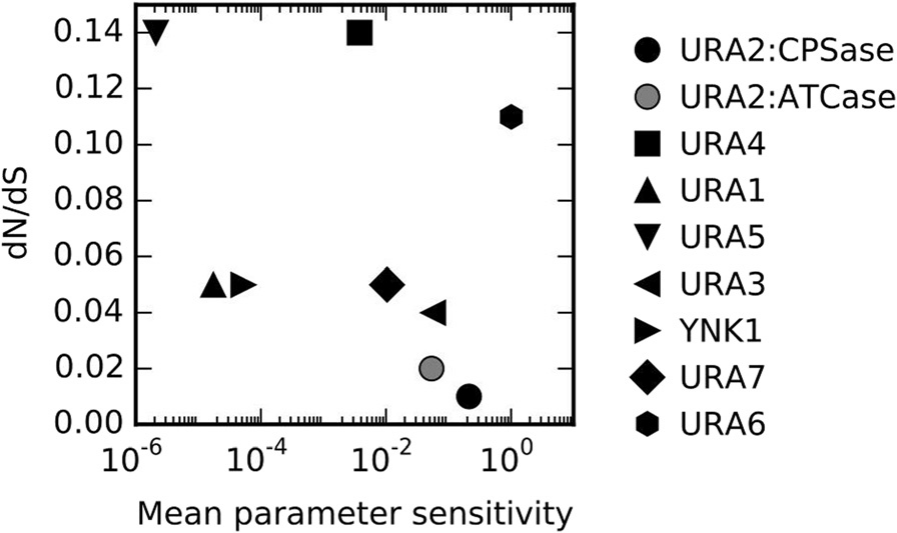
Negative correlation between protein evolutionary rate ratios and kinetic pathway sensitivity. With the exception of URA2, each point represents an enzyme in the pathway, for which we have calculated evolutionary rate ratio dN/dS and the geometric mean sensitivity of steady-state pathway flux to kinetic parameters of that enzyme. For URA2, we separately analyzed the domains corresponding to the two enzymatic activities it performs

To assess the relationship between the biochemical properties of the pathway and the evolution of the constituent enzymes, we assigned each enzymatic activity a single sensitivity score by taking the geometric mean of the sensitivities for the parameters it possesses, and we compared these sensitivities with evolutionary rate ratios. We find that enzymes for which the steady-state flux is sensitive tend to evolve more slowly (Fig. 3). The rank correlation is −0.485, with a suggestive although statistically insignificant p-value of 0.19. Interestingly, the two enzymatic activities of URA2 are predicted to have different effects on pathway flux, and the CPSase enzymatic activity with greater sensitivity indeed evolves more slowly. The most notable exception to the overall trend of decreasing evolutionary rate ratios with increasing pathway sensitivity is URA6. URA6 converts UMP to UDP, forming part of the negative allosteric feedback loop in the pathway. Our model includes only the most direct route from UMP to UTP, but other unmodeled metabolic pathways may be influential, perhaps leading our model to overestimate the influence of URA6. In particular, the substrate of URA6 is UMP, which can also be produced from uracil by the salvage pathway (Fig. 1), which we have not modeled. Including only the core reactions (URA2, URA4, URA1, URA5, URA3), we find a correlation of −0.841, *p* = 0.036, perhaps suggesting that our model more accurately captures the core linear pathway than the feedback loops.

Among the strongest known predictors of protein evolutionary rate are expression level and solvent accessibility. For the enzymes in the pyrimidine biosynthesis pathway, the rank correlation between dN/dS and expression level (r = +0.11, *p* = 0.79) is notably weaker than that between dN/dS and steady-state flux sensitivity. The correlation between dN/dS and absolute solvent accessibility (r = +0.46, *p* = 0.21) is similar to but slightly weaker than that between dN/dS and flux sensitivity. When expression level and solvent accessibility are controlled for, the overall correlation between dN/dS and flux sensitivity becomes somewhat weaker (r = −0.37, *p* = 0.36), but it remains strong for the core pathway genes (r = −0.94, *p* = 0.016). Together, these results suggest that flux sensitivity predicts short-term protein evolution as well as the strongest previously known predictors. Further, expression level contributes to selective constraint through both flux sensitivity mediated by the parameter V_max_ (that is dependent upon [E]) as well as through selection against negative (non-specific) effects when [E] becomes large.

## Conclusions

We found that enzymes with greater influence on flux through the yeast pyrimidine synthesis pathway tend to evolve under greater selective constraint, as measured by dN/dS over a short evolutionary time. This is consistent with a scenario in which deleterious changes are strongly purged from rate-limiting enzymes, because they alter pathway flux, but such changes may accumulate in enzymes with less control over flux (Mannakee and Gutenkunst, manuscript submitted). Genetic changes may also accumulate that alter the expression levels of the various enzymes. Over longer evolutionary periods, we expect that mutation-selection balance dominates this process (Orlenko, Teufel, and Liberles, manuscript submitted), and rate-limiting steps in the pathway may change. Selection may also act on more aspects of pathway function than steady-state flux, such as temporal dynamics, potentially creating a more rugged fitness landscape. However, even under more complex selective regimes, we still expect mutation-selection balance over longer evolutionary periods and stronger selection on enzymes temporarily in control points over shorter evolutionary periods. There are of course the caveats that the activity level of an enzyme will likely influence the relative proportion of mutations that improve or decrease function and that the expression level of an enzyme will increase selective constraint on that enzyme for reasons independent of the positive function of the enzyme.

In the study here, a link in short-term evolution between dN/dS and flux control was found. The phylogenetic analysis presented covers much longer evolutionary periods than the obvserved correlation between dN/dS and flux. The reasons for this are two-fold. First, dN/dS (in addition to other limitations) is limited in where it can be applied due to problems with rapid saturation of dS. Second, kinetic data is limited for most pathways over most of the tree of life. A discussion of differences in expectations of short and long evolutionary periods has been undertaken, and together these reflect gaps in data, methodology, and theory to address key problems in the functional synthesis of molecular evolution.

Ultimately, as the functional synthesis progresses in molecular evolution, questions about using functional evolution to explain dN/dS and observed substitution patterns more generally [33] will be flipped on their head. The question will then be, “Can the field develop good evolutionary metrics that are predictors of lineage-specific directional functional change between homologous proteins from closely related genomes?”.

## Methods

### Phylogenetic analysis

Sequences for each step in the *de novo* pyrimidine biosynthesis pathway of *S. cerevisiae* were downloaded from NCBI. Sequences downloaded from NCBI were as follows: URA1 (GI: 6322633), URA2 (GI: 330443609), URA3 (GI: 398364267), URA4 (GI: 6323452), URA5 (GI: 6323530), URA10 (GI: 6323927), URA6 (GI: 398364671), URA7 (GI: 6319432), and YNK1 (GI: 6322783). All sequences were from the laboratory strain *S. cerevisiae* S288c. A BLAST search against all fungal species was performed for each of the sequences to determine homologs from the non-redundant database. BLAST e-value thresholds were varied to include the largest amount of diversity possible. The e-value threshold used for URA gene families 2, 3, 4, 5, 6, 7, 10, and YNK1 was 1e^−10^ while the e-value cutoff for URA1 was 1. Where possible, families were extended such that each family would contain a sequence from 4 different fungal species. The related species that were attempted to be incorporated into each gene family were: *S. arboricola*, *Blumeria graminis*, *Schizosaccharomyces pombe*, and *Kluyveromyces polysporus,* selected as species with whole genomes designed to give a broad picture of fungal protein evolution*.* The initial gene families were then reduced based on size thresholds to limit the number of partial sequences within the datasets. The thresholds for size discrimination varied for each gene family, to include the four related species. Size cutoffs of 45 % were used for families URA1 and URA3, while a cutoff value of 10 % was used for families URA2, 4, 5, 7, and 10. Gene families for URA6 and YNK1 used a size threshold of 20 %. These resulted in gene family sizes of 188 proteins (representing 161 unique species, URA1), 199 proteins (representing 189 unique species, URA2), 297 proteins (representing 226 unique species, URA3), 233 proteins (representing 208 unique species, URA4), 244 proteins (representing 208 unique species, URA5), 246 proteins (representing 211 unique species, URA10), 260 proteins (representing 220 unique species, URA6), 358 proteins (representing 311 unique species, URA7) and 277 proteins (representing 264 unique species, YNK1). Multiple sequence alignments for each family were generated using MAFFT (L-ins-i) method [34], and optimal substitution models were calculated with Prottest 3.4 [35]. The best substitution model as calculated from Prottest was: LG + I + G + F (URA1), LG + I + G + F (URA2), LG + G (URA3), LG + I + G (URA4), LG + I + G (URA5), LG + I + G (URA10), LG + I + G (URA6), LG + I + G (URA7), LG + I + G (YNK1), LG + I + G + F (CPSase), LG + I + G (ATCase). These models were then used to calculate a phylogenetic tree for each URA protein family using PhyML 3.4 [36]. PhyML was run with 100 bootstraps and implemented differently depending on the substitution model and parameters found using Prottest. Total tree lengths varied for each of the gene families with total lengths as follows: 86.69 (URA1), 46.12 (URA2) 66.38 (URA3),61.51 (URA4), 47.98 (URA5), 48.76 (URA10), 127.61 (URA6), 75.61 (URA7), YNK1 (53.74). The URA1 gene family showed the second longest total tree length while also having the fewest number of species, suggesting higher levels of divergence than other URA gene families.

Upon reconstruction of the phylogenetic tree, each gene tree was then reconciled against the fungal species tree as found on NCBI using Softparsmap [37] to infer the root of each tree. The Softparsmap parameters that were used in the analysis were “did = root” to minimize the number of duplications and loss and to root the tree. Also the parameter allowing for weak nodes to be collapsed was set to 0.7. The removal of in-paralogous sequences was set to “no” so that paralogous sequences would still be present in the duplication analysis. The events that were identified as gene duplication events were meant as a conservative estimate of high confidence events, so evidence for multiple copies in at least one species rather than purely topological differences was required. The counts given are not meant to be reflective of underlying duplication or lateral transfer rates.

To determine dN/dS ratios (the ratio of nonsynonymous to synonymous substitution rates) for the *S. cerevisiae* 288c lineage within the *de novo* pyrimidine biosynthesis pathway in yeast, subtrees were selected from each of the larger URA gene family trees. Subtrees were selected such that the overall dS tree length was below 3, to control for potential dS saturation throughout the tree. All subtrees selected had an overall dS tree length of approximately 3 (or lower). Ratios for dN/dS were calculated for each subtree with PAML 4.5 [38], using the free-ratios branch model and the 2-ratio branch model (for URA1, URA3, URA5, and URA10). The 2-ratio branch model was configured such that the branch leading to *S. cerevisiae* S288c was estimated independently from the rest of the tree branches for genes where this species was used for kinetic data. To test the robustness of dN/dS ratios dependent on initial starting values of dN/dS in PAML, three different starting values of dN/dS were used: 0.5, 1, and 2. Only the URA6 and URA7 gene families showed fluctuations in dN/dS values for the free-ratios model due to different initial starting values of dN/dS. However each of the fluctuating branches also showed an extremely low value for dS, were excluded from the analysis, and did not affect the dN/dS ratio estimate for the family. The models formed a nested hierarchy of complexity for model testing, comparing the free ratio to two ratio and both to the one ratio (for cases where the two ratio was not supported but the free ratio was), with p-values calculated from a χ^2^ distribution. For lineages where dS < 0.01, the one ratio value was used in place of the free-ratios value. No correction for multiple testing was applied, as the aim of this analysis is to identify the best supported dN/dS ratio without over-fitting the data.

To further understand how perturbations to the phylogenetic tree could impact the relative rates of dN/dS, for each tree that did not show support for the free-ratios branch model, a subtree was built one node below the original subtree and evaluated for dN/dS using the same models as described above. For URA2 and URA4, although both gene trees supported the free-ratios branch model, the dN/dS ratio for the 1-ratio model was used in subsequent analyses, since the *S. cerevisiae* S288c strain was not used as the experimental strain in the kinetic parameter estimates. Thus to attain a more general approximation of the relative rate of evolution along the *S. cerevisiae* S288c lineage, only dN/dS values for the 1-ratio branch model were used for these trees.

The URA2 gene family was explored further based on domain boundaries within the protein to determine if different domains within the protein influenced the overall dN/dS of the protein family. The URA2 gene is composed of four subdomains, from which the CPSase (carbamoyl-phosphate synthase) and the ATCase (aspartate transcarbamylase) domains were examined independently to determine the dN/dS ratio within the domain. The CPSase domain region (441–1482) and the ATCase (1910 – 2214) regions (see UniProtKB – P07259) were extracted from the URA2 protein and analyzed phylogenetically using the same methods described above to determine if an elevated dN/dS ratio was detectable within either of the domains compared to the overall dN/dS of the protein.

### Kinetic modeling

The structures of the pathway in *E. coli* and *S. cerevisiae* are similar, with two exceptions (Fig. 1b). First, the first two reactions in the pathway, carbamoyl phosphate synthetase (CPSase) and aspartate carbamoyltransferase (ATCase), occur on separate enzymes in *E. coli* and on the single enzyme URA2 in *S. cerevisiae*. Second, allosteric regulation of CPSase and ATCase is simpler in *S. cerevisiae* than in *E coli*. In *S. cerevisiae*, both activities are allosterically inhibited by UTP, while in *E. coli* there are multiple allosteric regulators. All reactions were modeled with Michaelis-Menten kinetics. We also included terms in our equations accounting for dilution of all reactants due to cell growth. We set the dilution rate to d = 0.11/hr, to match the conditions of the chemostat experiment with which we compare metabolite concentrations [39]. The complete set of equations is reproduced in Additional file 1. All computations with the kinetic model were performed in SloppyCell [40].

The model contains 22 parameters for the biochemistry of the enzymes, 4 parameters for input metabolite concentrations, and 2 parameters for cellular utilization of pyrimidines. To assign values to these parameters, we first gathered published in vitro biochemical data on the enzymes and mass spectrometry data on metabolite concentrations (“experimental” reference values in Table 1). We found experimental data for all but 5 of the model parameters. To assign values to these parameters, we initially sought to optimize their values to reproduce experimentally measured steady-state concentrations of UMP, UDP, and UTP, by minimizing the least-squares cost function

1 $$ {\left(\frac{\left[\mathrm{u}\mathrm{m}\mathrm{p}\right]\ \hbox{--}\ 0.37\ \upmu \mathrm{M}}{2.5\ \upmu \mathrm{M}}\right)}^2+{\left(\frac{\left[\mathrm{u}\mathrm{d}\mathrm{p}\right]\ \hbox{--}\ 2.9\ \upmu \mathrm{M}}{2.5\ \upmu \mathrm{M}}\right)}^2+{\left(\frac{\left[\mathrm{u}\mathrm{t}\mathrm{p}\right]\ \hbox{--}\ 6.7\ \upmu \mathrm{M}}{2.5\ \upmu \mathrm{M}}\right)}^2 $$

We were, however, unable to find a suitable parameter set. This is not surprising, because these data come from multiple sources, so they are not consistent with respect to measurement conditions, which can cause models predictions to be inaccurate [41]. We thus undertook another series of parameter optimizations, in which we allowed all 28 parameters to vary. To incorporate the experimental parameter measurements into our optimization, for the 23 parameters for which we had experimental measurements, we added terms to the cost function of the form

2 $$ \frac{{\left( \ln\ p\hbox{--}\ \ln\ {p}_0\right)}^2}{ \ln\ 1000} $$

Here *p* denotes the value of the parameter in the set being evaluated, and *p*_*0*_ denotes the experimentally measured value of the parameter. In a Bayesian likelihood framework, these terms correspond to priors that put 95 % of the prior density within three orders of magnitude larger or smaller than the experimental value *p*_*0*_. For the 5 parameters for which we did not have yeast experimental data, we constrained the parameter more loosely to be near the *E. coli* value reported by Rodriguez et al. [30], via terms of the form

3 $$ \frac{{\left( \ln\ p\hbox{--}\ \ln\ {p}_0\right)}^2}{ \ln\ {10}^7} $$

To ensure convergence of our optimizations, we ran 100 different local optimizations from randomly assigned starting parameter sets.

For each parameter set, we calculated the sensitivity of the model to changes in each parameter as the normalized first derivative of the steady state concentration of UMP with respect to that parameter, i.e. the magnitude of the change in steady state UMP concentration resulting from a small change in the value of the parameter: $$ \raisebox{1ex}{$d\left[\mathrm{u}\mathrm{m}\mathrm{p}\right]$}\!\left/ \!\raisebox{-1ex}{$dp$}\right.\times \raisebox{1ex}{$p$}\!\left/ \!\raisebox{-1ex}{$\left[\mathrm{u}\mathrm{m}\mathrm{p}\right]$}\right. $$. This derivative was calculated using a central finite difference method with a step size of 1 % of the parameter value. The mean spearman rank correlation of between sensitivities calculated from different parameter sets was 0.998, so we report only results from the parameter set with the lowest total cost (Equations 1–3). We calculated the overall sensitivity of the model to changes in each modeled enzyme as the geometric mean of the sensitivities of the reaction parameters associated with the enzyme.

In *S. cerevisiae,* the fifth step in the pathway can be catalyzed by both URA5 and URA10. However, in wild-type cells 80 % of the OPRtase activity is due to URA5 [42], and URA5 has been much more extensively kinetically characterized than URA10 [43]. We thus based our model parameters on URA5 and compared kinetic sensitivity of the OPRtase step of the pathway with the evolutionary rate ratio of URA5. Similarly, URA7 and URA8 have overlapping CTP synthetase activity. URA7 is, however, responsible for the majority of CTP synthesis [44], so we consider only URA7 in our analysis.

### Expression and solvent accessibility

Expression data at mid-log phase in units of molecules/cell was obtained from Holstege et al. [45]. Per-residue solvent accessibility as predicted by SPINEX [46] was obtained from [47]. For correlation analysis, we used the mean solvent accessibility of the residues in each enzyme.

### Availability of supporting data

The data sets and analysis supporting the results of this article are included within the article and associated supplemental materials.

## Additional file

Additional file 1: **Supplementary materials.** Supplementary Figures contain the equations that were used in the kinetic model and all phylogenetic results that support the summary in Fig. 2 and results described in the paper. (PDF 13746 kb)
