# Supplementary material for: Characterizing selective pressures on the pathway for de novo biosynthesis of pyrimidines in yeast
Source: BMC Evol Biol. 2015 Oct 28;15:232. doi: 10.1186/s12862-015-0515-x (PMC4625875; doi:10.1186/s12862-015-0515-x)
Supplement: Supplementary file 1 — Supplementary materials. Supplementary Figures contain the equations that were used in the kinetic model and all phylogenetic results that support the summary in Fig. 2 and results described in the paper. (PDF 13746 kb) [file 12862_2015_515_MOESM1_ESM.pdf]

Model Equations

$$\frac{d[\mathrm{cp}]}{dt}=\frac{\mathrm{vmax1}\ [\mathrm{bc}][\mathrm{glu}][\mathrm{atp}]}{\left(1+\frac{[\mathrm{utp}]}{\mathrm{Kutp}}\right)(\mathrm{K_{atp}}+[\mathrm{atp}])(\mathrm{K_{bc}}+[\mathrm{bc}])(\mathrm{K_q}+[\mathrm{glu}])}-\frac{\mathrm{vmax2}\ [\mathrm{cp}][\mathrm{asp}]}{\left(1+\frac{[\mathrm{utp}]}{\mathrm{Kutp}}\right)(\mathrm{K_{m2}}+[\mathrm{cp}])(\mathrm{K_{asp}}+[\mathrm{asp}])}-\mathrm{d}[\mathrm{cp}]$$

$$\frac{d[\mathrm{ca}]}{dt}=\frac{\mathrm{vmax2}\ [\mathrm{cp}][\mathrm{asp}]}{\left(1+\frac{[\mathrm{utp}]}{\mathrm{Kutp}}\right)(\mathrm{K_{m2}}+[\mathrm{cp}])(\mathrm{K_{asp}}+[\mathrm{asp}])}-\frac{\mathrm{vmax3}\ [\mathrm{ca}]}{\mathrm{K_{m3}}+[\mathrm{ca}]}-\mathrm{d}[\mathrm{ca}]$$

$$\frac{d[\mathrm{dho}]}{dt}=\frac{\mathrm{vmax3}\ [\mathrm{ca}]}{\mathrm{K_{m3}}+[\mathrm{ca}]}-\frac{\mathrm{vmax4}\ [\mathrm{dho}]}{\mathrm{K_{m4}}+[\mathrm{dho}]}-\mathrm{d}[\mathrm{dho}]$$

$$\frac{d[\mathrm{oro}]}{dt}=\frac{\mathrm{vmax4}\ [\mathrm{dho}]}{\mathrm{K_{m4}}+[\mathrm{dho}]}-\frac{\mathrm{vmax5}\ [\mathrm{oro}][\mathrm{prpp}]}{\mathrm{K_{m5}}+[\mathrm{oro}][\mathrm{prpp}]}-\mathrm{d}[\mathrm{oro}]$$

$$\frac{d[\mathrm{omp}]}{dt}=\frac{\mathrm{vmax5}\ [\mathrm{oro}][\mathrm{prpp}]}{\mathrm{K_{m5}}+[\mathrm{oro}][\mathrm{prpp}]}-\frac{\mathrm{vmax6}\ [\mathrm{omp}]}{\mathrm{K_{m6}}+[\mathrm{omp}]}-\mathrm{d}[\mathrm{omp}]$$

$$\frac{d[\mathrm{ump}]}{dt}=\frac{\mathrm{vmax6}\ [\mathrm{omp}]}{\mathrm{K_{m6}}+[\mathrm{omp}]}-\frac{\mathrm{vmax10}\ [\mathrm{ump}]}{\mathrm{K_{m10}}+[\mathrm{ump}]}-\mathrm{d}[\mathrm{ump}]$$

$$\frac{d[\mathrm{udp}]}{dt}=\frac{\mathrm{vmax10}\ [\mathrm{ump}]}{\mathrm{K_{m10}}+[\mathrm{ump}]}-\frac{\mathrm{vmax7}\ [\mathrm{udp}]}{\mathrm{K_{m7}}+[\mathrm{udp}]}-\mathrm{d}[\mathrm{udp}]$$

$$\frac{d[\mathrm{utp}]}{dt}=\frac{\mathrm{vmax7}\ [\mathrm{udp}]}{\mathrm{K_{m7}}+[\mathrm{udp}]}-\frac{\mathrm{vmax8}\ [\mathrm{utp}]}{\mathrm{K_{m8}}+[\mathrm{utp}]}-\frac{\mathrm{g_{pyr}}\ [\mathrm{utp}]}{\mathrm{K_{Mp}}+[\mathrm{utp}]}-\mathrm{d}[\mathrm{utp}]$$

$$\frac{d[\mathrm{ctp}]}{dt}=\frac{\mathrm{vmax8}\ [\mathrm{utp}]}{\mathrm{K_{m8}}+[\mathrm{utp}]}-\frac{\mathrm{g_{pyr}}\ [\mathrm{ctp}]}{\mathrm{K_{Mp}}+[\mathrm{ctp}]}-\mathrm{d}[\mathrm{ctp}]$$

Supplemental Figure 1. Lineage-specific dN/dS estimates for the local URA1 tree around *Saccharomyces cerevisiae*. Numbers in bold are the dN/dS values for the given branch.

Supplemental Figure 2 Lineage-specific dN/dS estimates for the local URA2 tree around *Saccharomyces cerevisiae*. Numbers in bold are the dN/dS values for the given branch.

Supplemental Figure 3. Lineage-specific dN/dS estimates for the local URA3 tree around *Saccharomyces cerevisiae*. Numbers in bold are the dN/dS values for the given branch.

Supplemental Figure 4. Lineage-specific dN/dS estimates for the local URA4 tree around *Saccharomyces cerevisiae*. Numbers in bold are the dN/dS values for the given branch.

Supplemental Figure 5. Lineage-specific dN/dS estimates for the local URA5 tree around *Saccharomyces cerevisiae*. Numbers in bold are the dN/dS values for the given branch.

Supplemental Figure 6. Lineage-specific dN/dS estimates for the local URA10 tree around *Saccharomyces cerevisiae*. Numbers in bold are the dN/dS values for the given branch.

Supplemental Figure 7. Lineage-specific dN/dS estimates for the local URA6 tree around *Saccharomyces cerevisiae*. Numbers in bold are the dN/dS values for the given branch.

Supplemental Figure 8. Lineage-specific dN/dS estimates for the local URA7 tree around *Saccharomyces cerevisiae*. Numbers in bold are the dN/dS values for the given branch.

Supplemental Figure 9. Lineage-specific dN/dS estimates for the local YNK1 tree around *Saccharomyces cerevisiae*. Numbers in bold are the dN/dS values for the given branch.

Supplemental Figure 10. Lineage-specific dN/dS estimates for the local ATCase tree around *Saccharomyces cerevisiae*. Numbers in bold are the dN/dS values for the given branch.

Supplemental Figure 11. Lineage-specific dN/dS estimates for the local CPSase tree around *Saccharomyces cerevisiae*. Numbers in bold are the dN/dS values for the given branch.

Supplemental Figure 12. Phylogeny of the URA1 gene family. **A.** Complete phylogeny of URA1 gene family. Taxa used in dN/dS analysis are colored in dark gray. **B.** Collapsed version of the URA1 gene family. Subtree used in the dN/dS analysis is marked in gray.

Supplemental Figure 13. Phylogeny of the URA2 gene family. **A.** Complete phylogeny of URA2 gene family. Taxa used in dN/dS analysis are colored in dark gray. **B.** Collapsed version of the URA2 gene family. Subtree used in the dN/dS analysis is marked in gray.

Supplemental Figure 14. Phylogeny of the URA3 gene family. **A.** Complete phylogeny of URA3 gene family. Taxa used in dN/dS analysis are colored in dark gray. **B.** Collapsed version of the URA3 gene family. Subtree used in the dN/dS analysis is marked in gray.

Supplemental Figure 15. Phylogeny of the URA4 gene family. **A.** Complete phylogeny of URA4 gene family. Taxa used in dN/dS analysis are colored in dark gray. **B.** Collapsed version of the URA4 gene family. Subtree used in the dN/dS analysis is marked in gray.

Supplemental Figure 16. Phylogeny of the URA5 gene family. **A.** Complete phylogeny of URA5 gene family. Taxa used in dN/dS analysis are colored in dark gray. **B.** Collapsed version of the URA5 gene family. Subtree used in the dN/dS analysis is marked in gray.

Supplemental Figure 17. Phylogeny of the URA10 gene family. **A.** Complete phylogeny of URA10 gene family. Taxa used in dN/dS analysis are colored in dark gray. **B.** Collapsed version of the URA10 gene family. Subtree used in the dN/dS analysis is marked in gray.

Supplemental Figure 18. Phylogeny of the URA6 gene family. **A.** Complete phylogeny of the URA6 gene family. Taxa used in dN/dS analysis are colored in dark gray. **B.** Collapsed version of the URA6 gene family. Subtree used in the dN/dS analysis is marked in gray.

Supplemental Figure 19. Phylogeny of the URA7 gene family. **A.** Complete phylogeny of the URA7 gene family. Taxa used in dN/dS analysis are colored in dark gray. **B.** Collapsed version of the URA7 gene family. Subtree used in the dN/dS analysis is marked in gray.

Supplemental Figure 20. Phylogeny of the YNK1 gene family. **A.** Complete phylogeny of the YNK1 gene family. Taxa used in dN/dS analysis are colored in dark gray. **B.** Collapsed version of the YNK1 gene family. Subtree used in the dN/dS analysis is marked in gray.

Supplemental Figure 21. Phylogeny of the ATCase domain of the URA2 gene family . **A.** Complete phylogeny of the ATCase domain family. Taxa used in dN/dS analysis are colored in dark gray. **B.** Collapsed version of the ATCase domain family. Subtree used in the dN/dS analysis is marked in gray.

Supplemental Figure 22. Phylogeny of the CPSase domain of the URA2 gene family . **A.** Complete phylogeny of the CPSase domain family. Taxa used in dN/dS analysis are colored in dark gray. **B.** Collapsed version of the CPSase domain family. Subtree used in the dN/dS analysis is marked in gray.

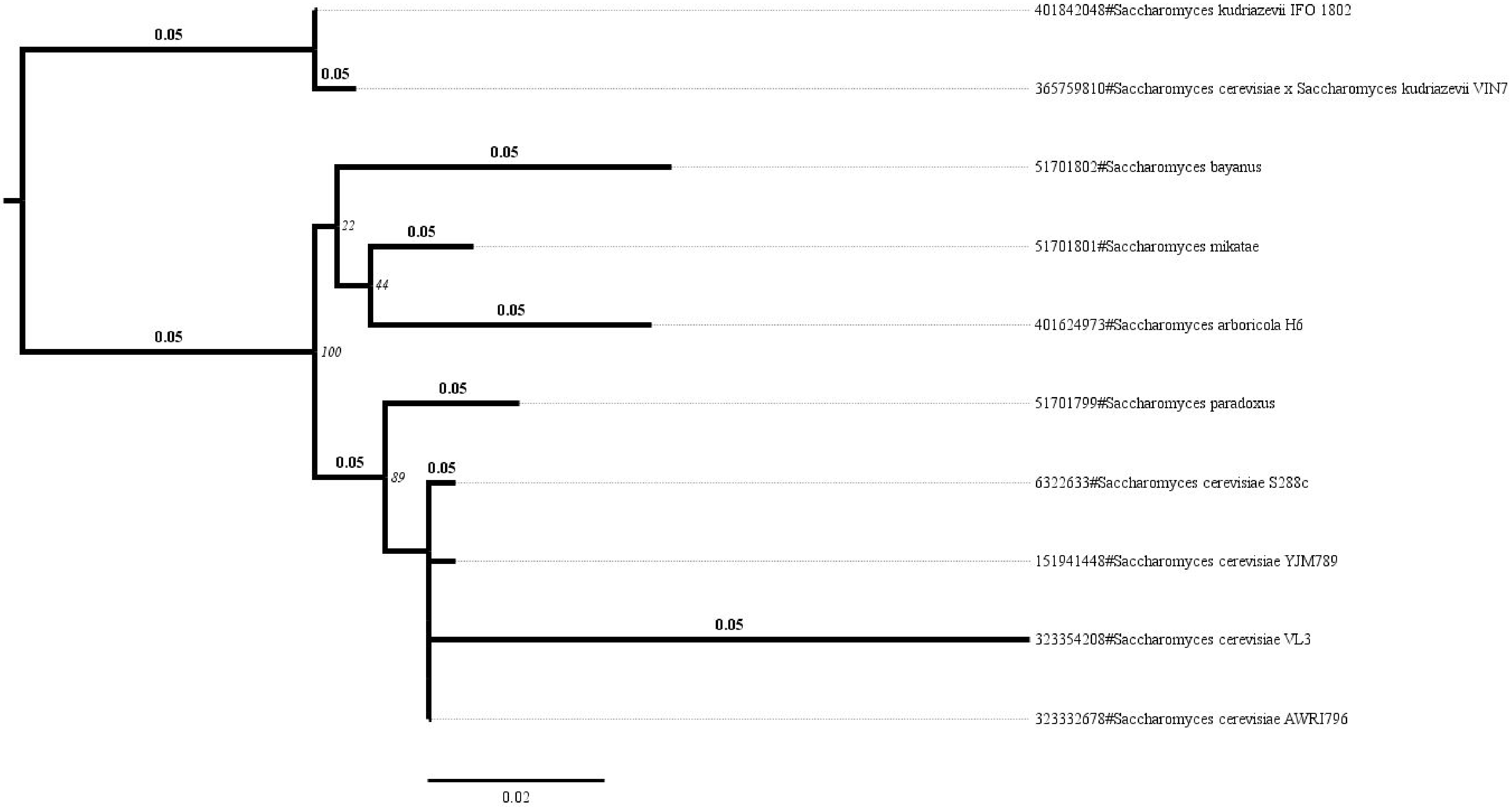
0.05
401842048#Saccharomyces kudriazevii IFO 1802
0.05
365759810#Saccharomyces cerevisiae x Saccharomyces kudriazevii VIN7
0.05
51701802#Saccharomyces bayanus
22
0.05
51701801#Saccharomyces mikatae
44
0.05
401624973#Saccharomyces arboricola H6
0.05
100
0.05
51701799#Saccharomyces paradoxus
0.05
89
0.05
6322633#Saccharomyces cerevisiae S288c
151941448#Saccharomyces cerevisiae YJM789
0.05
323354208#Saccharomyces cerevisiae VL3
323332678#Saccharomyces cerevisiae AWRI796
0.02

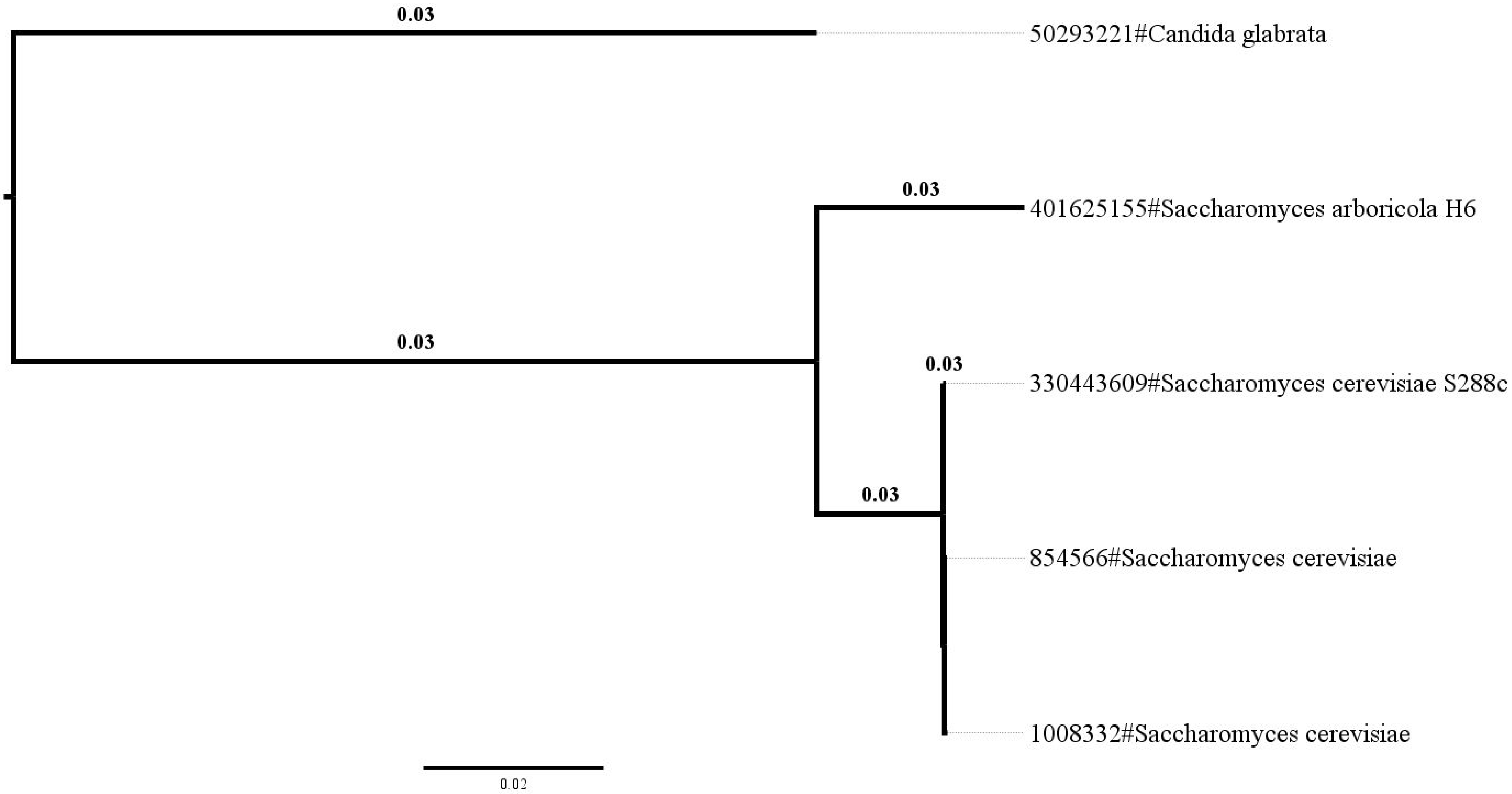
0.03
50293221#Candida glabrata
0.03
401625155#Saccharomyces arboricola H6
0.03
0.03
330443609#Saccharomyces cerevisiae S288c
0.03
854566#Saccharomyces cerevisiae
1008332#Saccharomyces cerevisiae
0.02

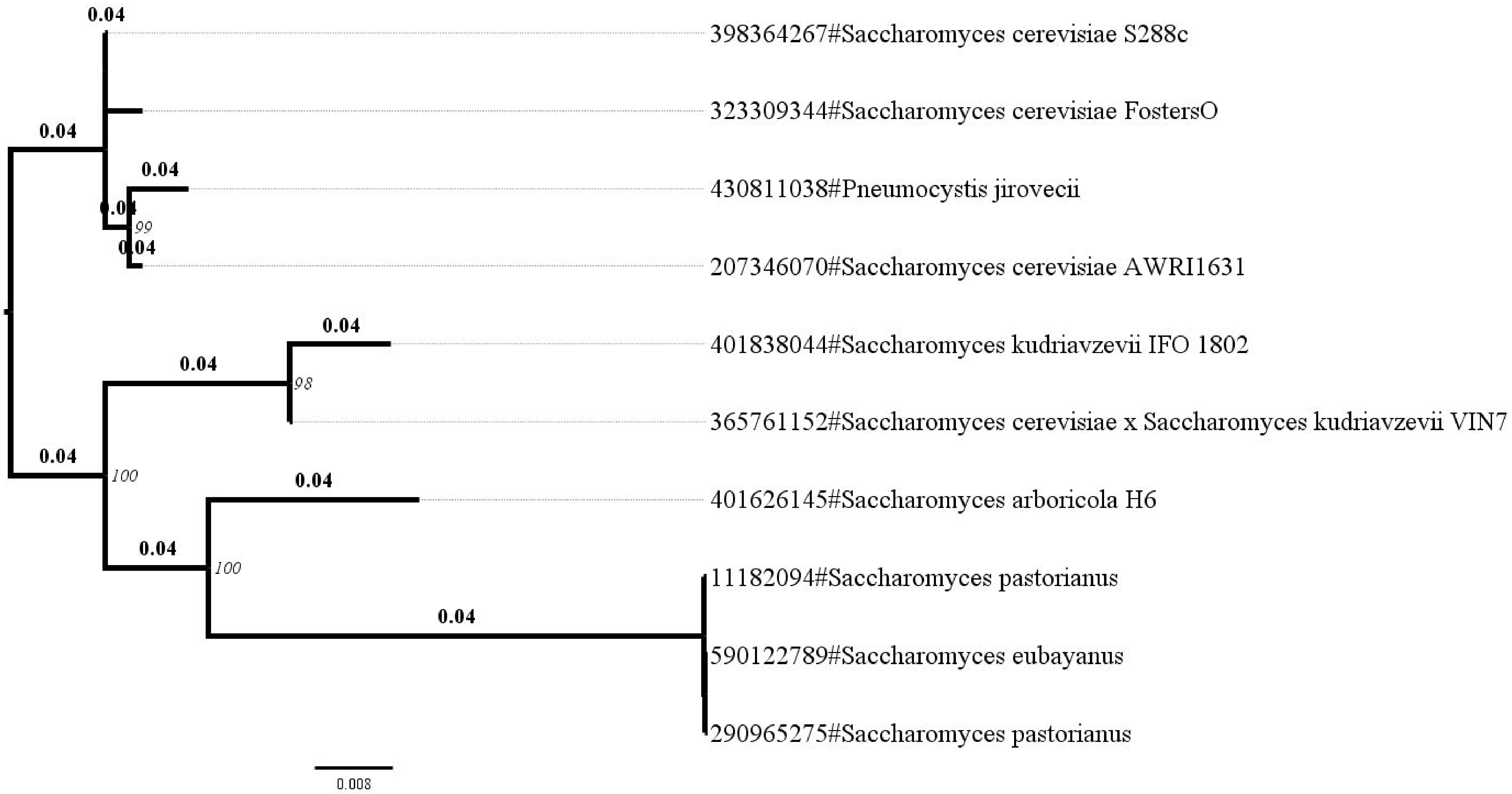
0.04
398364267#Saccharomyces cerevisiae S288c
323309344#Saccharomyces cerevisiae FostersO
0.04
0.04
430811038#Pneumocystis jirovecii
0.04
99
0.04
207346070#Saccharomyces cerevisiae AWRI1631
0.04
401838044#Saccharomyces kudriavzevii IFO 1802
0.04
98
365761152#Saccharomyces cerevisiae x Saccharomyces kudriavzevii VIN7
0.04
100
0.04
401626145#Saccharomyces arboricola H6
0.04
100
11182094#Saccharomyces pastorianus
0.04
590122789#Saccharomyces eubayanus
290965275#Saccharomyces pastorianus
0.008

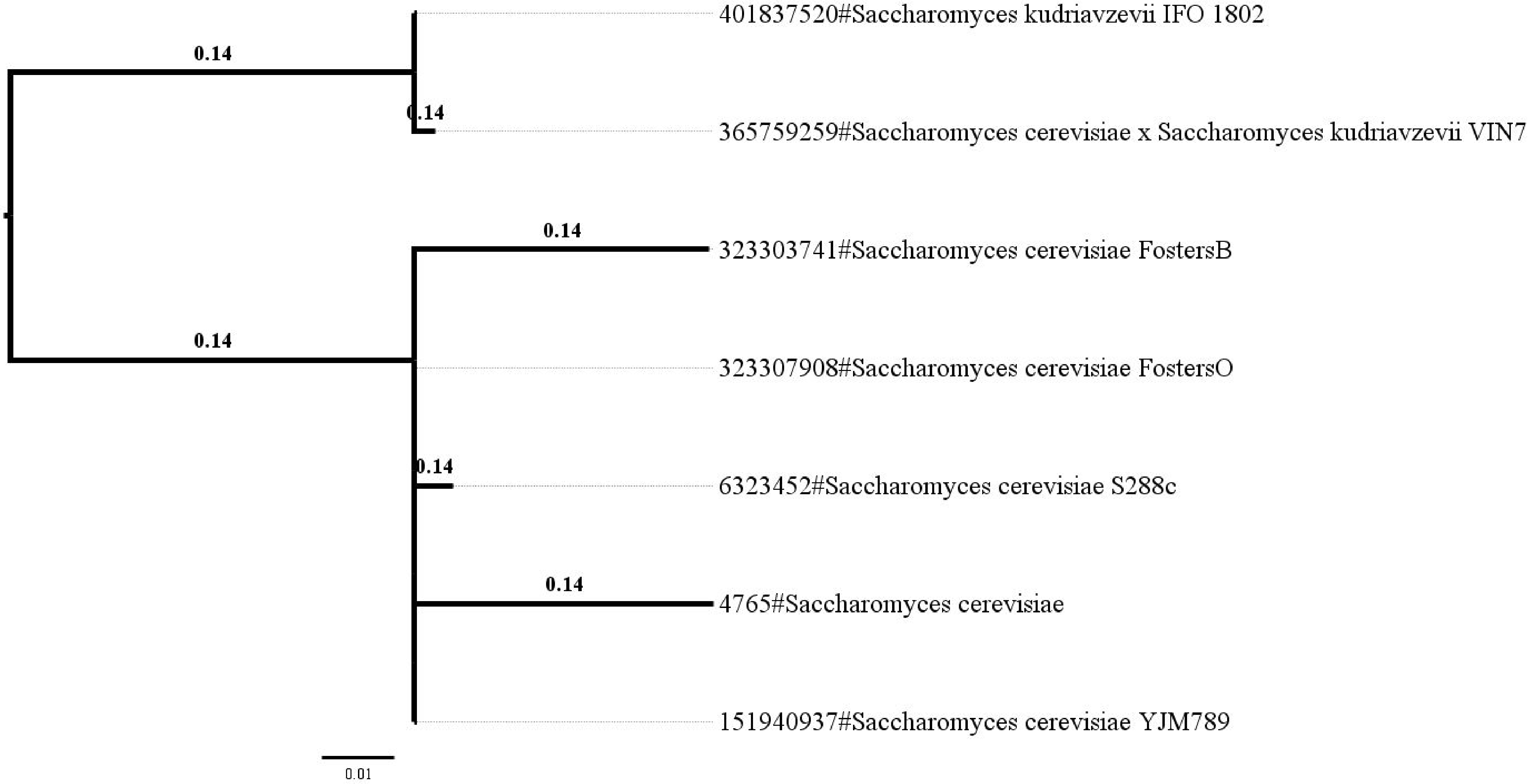
0.14
0.14
401837520#Saccharomyces kudriavzevii IFO 1802
365759259#Saccharomyces cerevisiae x Saccharomyces kudriavzevii VIN7
0.14
323303741#Saccharomyces cerevisiae FostersB
0.14
323307908#Saccharomyces cerevisiae FostersO
0.14
6323452#Saccharomyces cerevisiae S288c
0.14
4765#Saccharomyces cerevisiae
151940937#Saccharomyces cerevisiae YJM789
0.01

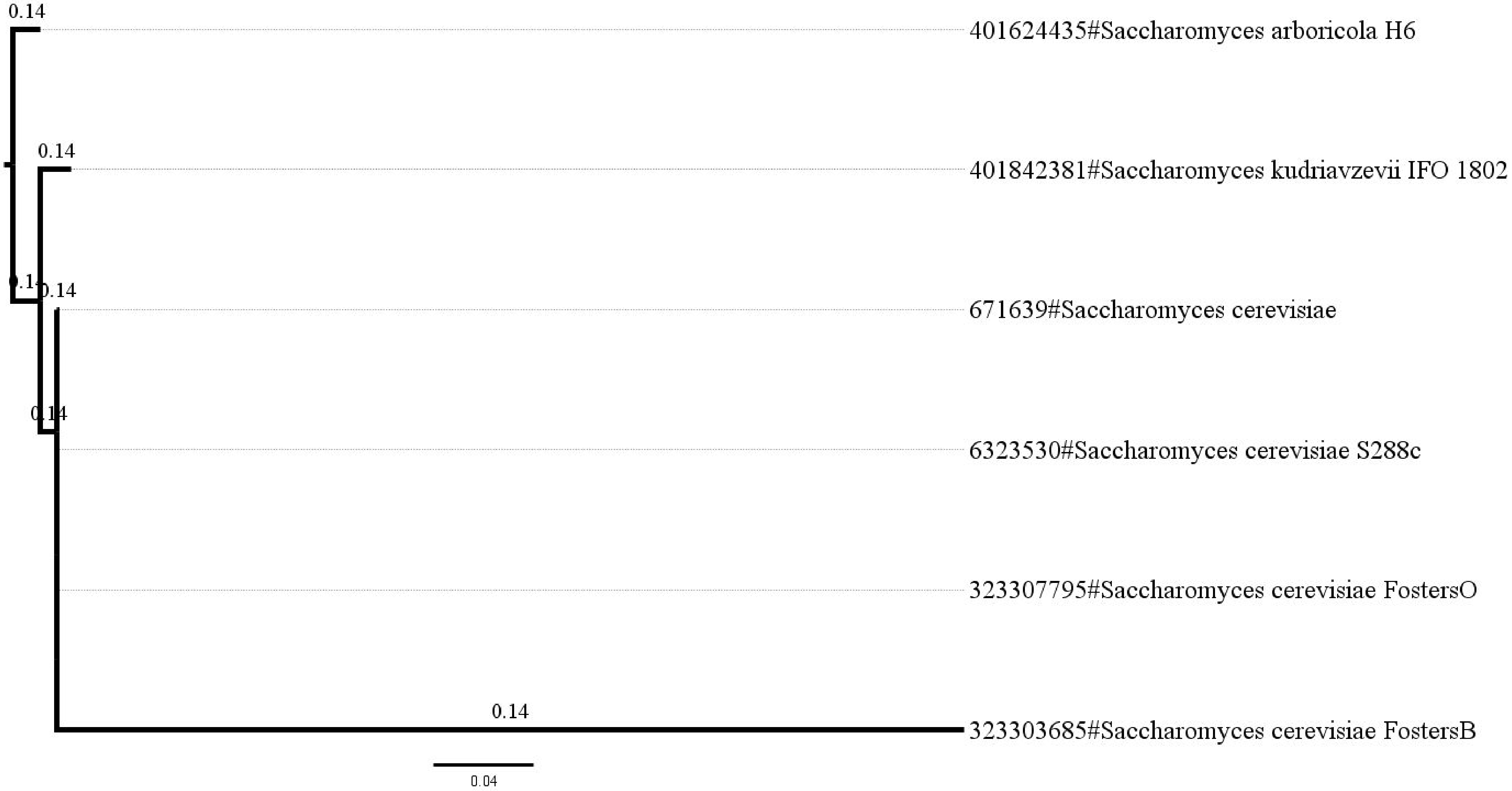
0.14
401624435#Saccharomyces arboricola H6
0.14
401842381#Saccharomyces kudriavzevii IFO 1802
0.14
671639#Saccharomyces cerevisiae
0.14
6323530#Saccharomyces cerevisiae S288c
323307795#Saccharomyces cerevisiae FostersO
0.14
323303685#Saccharomyces cerevisiae FostersB
0.04

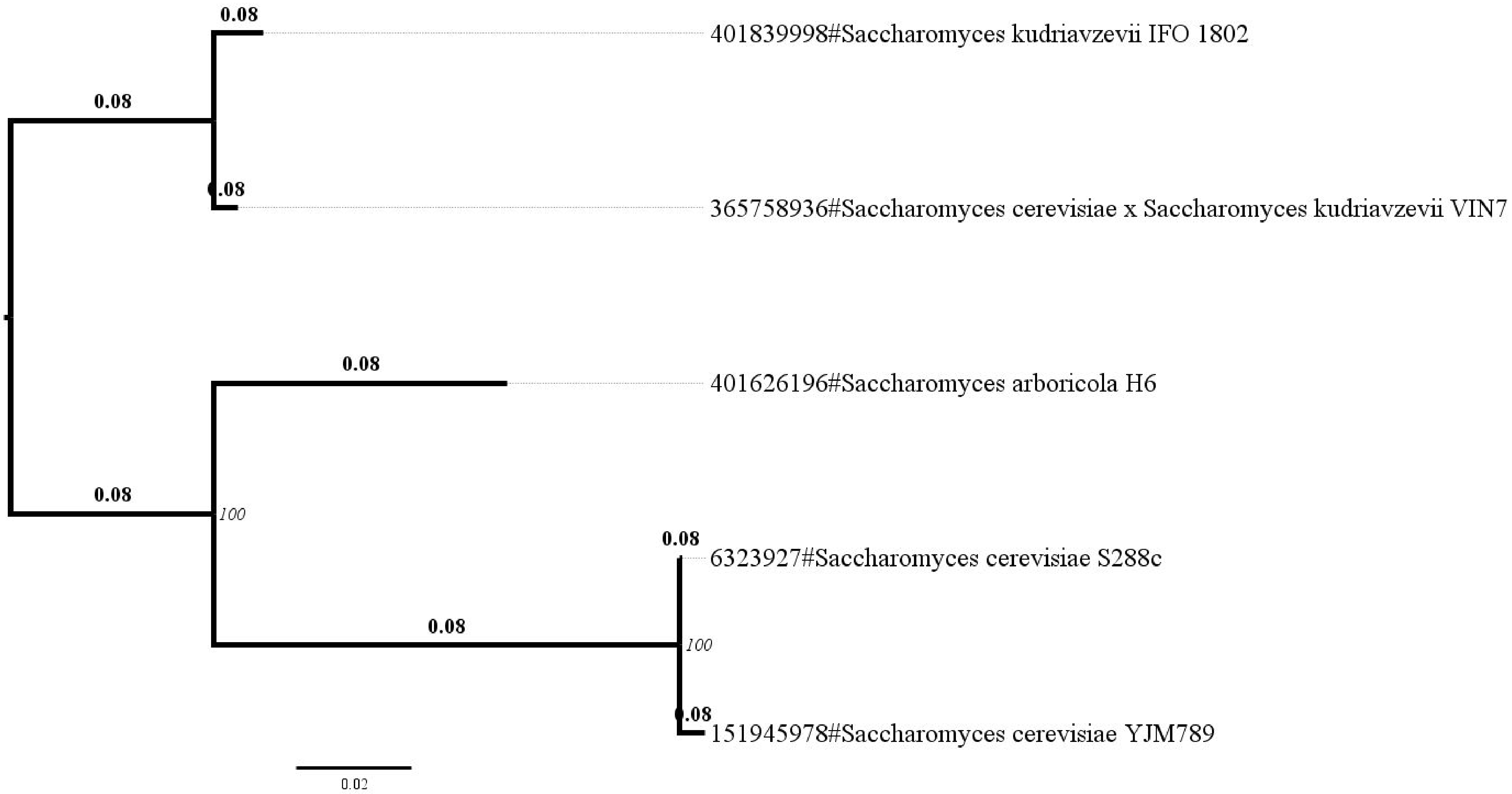
0.08
401839998#Saccharomyces kudriavzevii IFO 1802
0.08
0.08
365758936#Saccharomyces cerevisiae x Saccharomyces kudriavzevii VIN7
0.08
401626196#Saccharomyces arboricola H6
0.08
100
0.08
6323927#Saccharomyces cerevisiae S288c
0.08
100
0.08
151945978#Saccharomyces cerevisiae YJM789
0.02

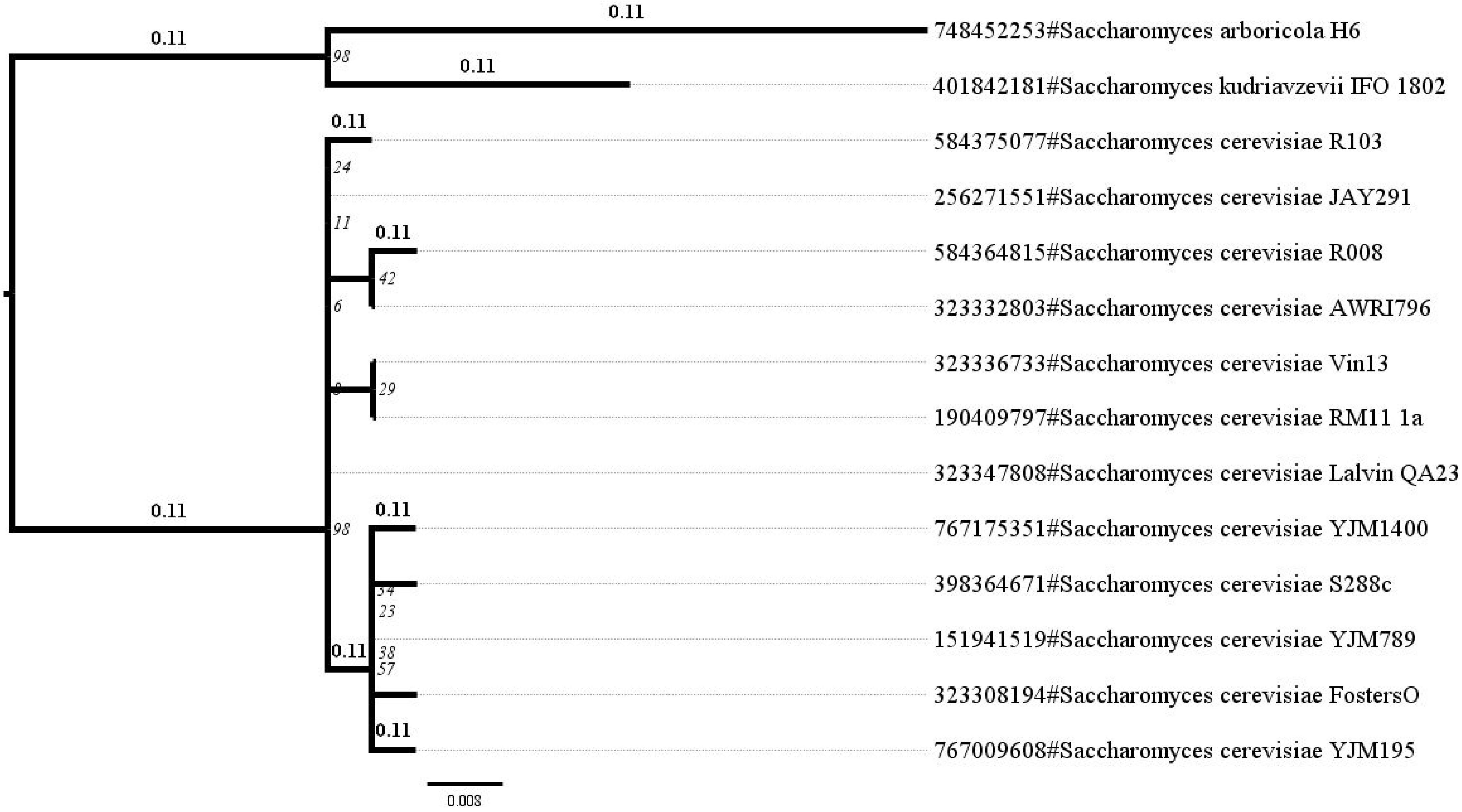

0.11
0.11
0.11
0.11
0.11
0.11
0.11
0.11
98
24
11
42
6
29
98
34
23
38
57
748452253#Saccharomyces arboricola H6
401842181#Saccharomyces kudriavzevii IFO 1802
584375077#Saccharomyces cerevisiae R103
256271551#Saccharomyces cerevisiae JAY291
584364815#Saccharomyces cerevisiae R008
323332803#Saccharomyces cerevisiae AWRI796
323336733#Saccharomyces cerevisiae Vin13
190409797#Saccharomyces cerevisiae RM11 1a
323347808#Saccharomyces cerevisiae Lalvin QA23
767175351#Saccharomyces cerevisiae YJM1400
398364671#Saccharomyces cerevisiae S288c
151941519#Saccharomyces cerevisiae YJM789
323308194#Saccharomyces cerevisiae FostersO
767009608#Saccharomyces cerevisiae YJM195
0.008

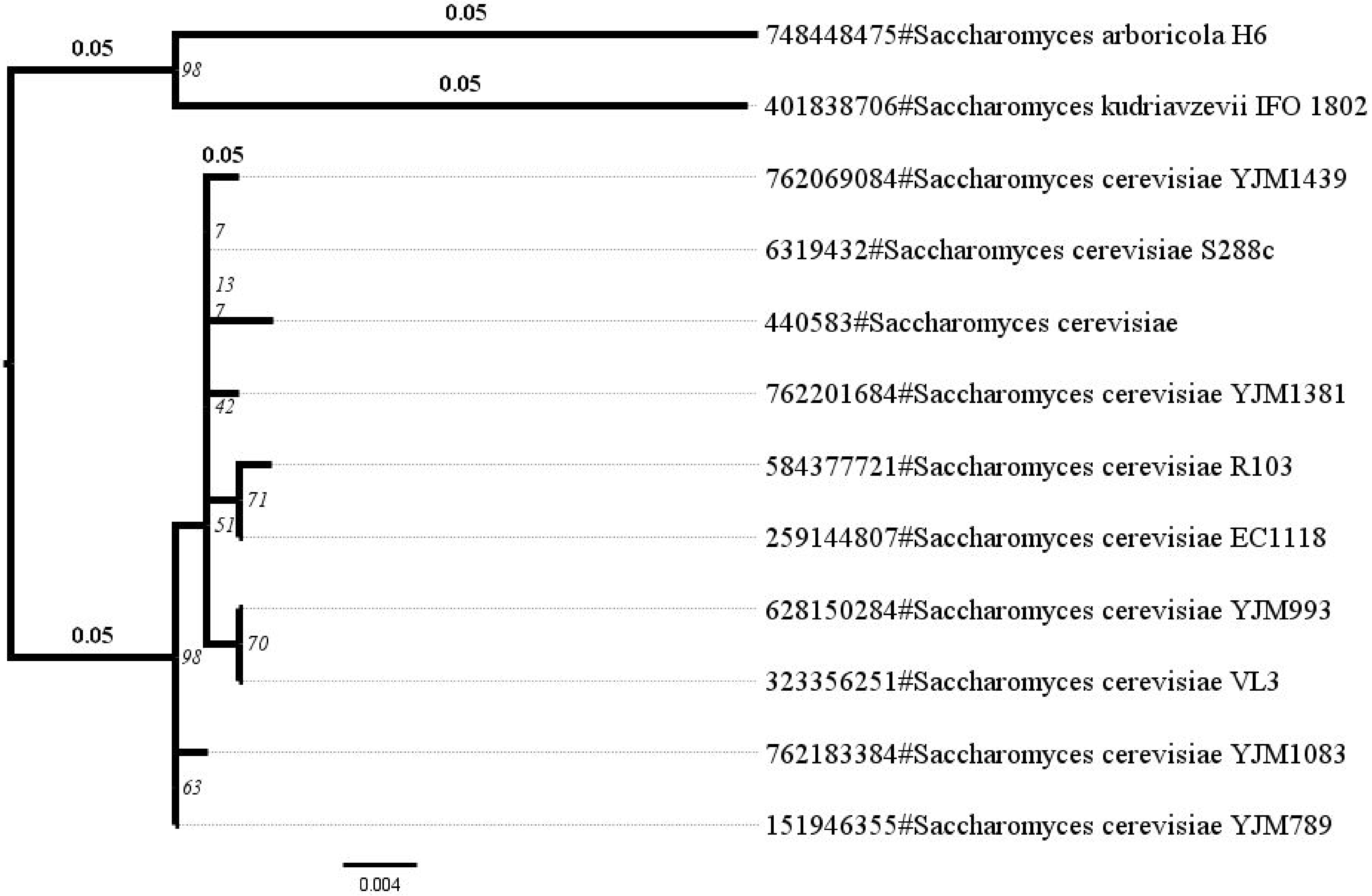
0.05
0.05
748448475#Saccharomyces arboricola H6
98
0.05
401838706#Saccharomyces kudriavzevii IFO 1802
0.05
762069084#Saccharomyces cerevisiae YJM1439
7
6319432#Saccharomyces cerevisiae S288c
13
7
440583#Saccharomyces cerevisiae
762201684#Saccharomyces cerevisiae YJM1381
42
584377721#Saccharomyces cerevisiae R103
71
51
259144807#Saccharomyces cerevisiae EC1118
628150284#Saccharomyces cerevisiae YJM993
0.05
70
98
323356251#Saccharomyces cerevisiae VL3
762183384#Saccharomyces cerevisiae YJM1083
63
151946355#Saccharomyces cerevisiae YJM789
0.004

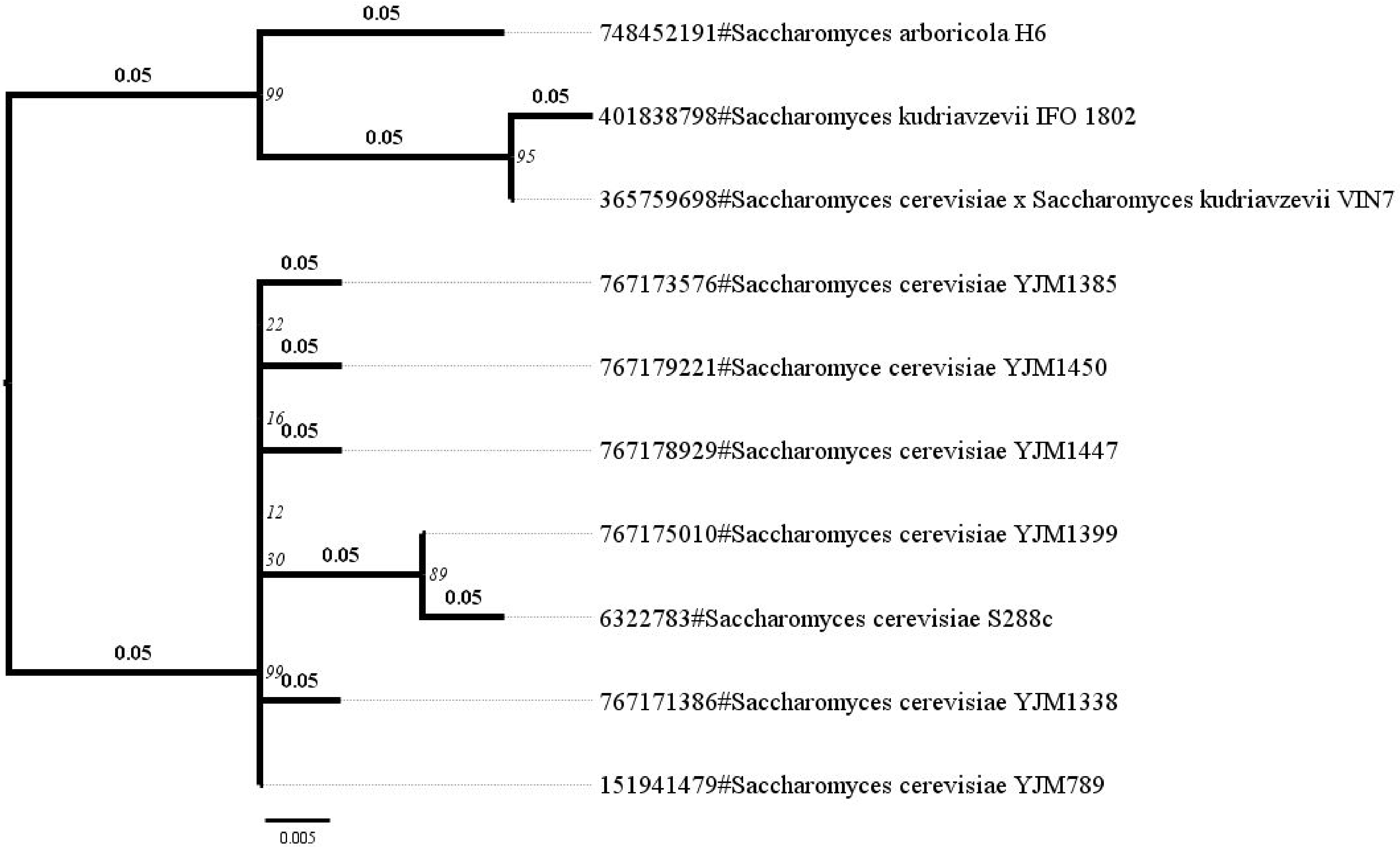
0.05
748452191#Saccharomyces arboricola H6
0.05
99
0.05
401838798#Saccharomyces kudriavzevii IFO 1802
0.05
95
365759698#Saccharomyces cerevisiae x Saccharomyces kudriavzevii VIN7
0.05
767173576#Saccharomyces cerevisiae YJM1385
22
0.05
767179221#Saccharomyce cerevisiae YJM1450
16
0.05
767178929#Saccharomyces cerevisiae YJM1447
12
767175010#Saccharomyces cerevisiae YJM1399
30
0.05
89
0.05
6322783#Saccharomyces cerevisiae S288c
0.05
99
0.05
767171386#Saccharomyces cerevisiae YJM1338
151941479#Saccharomyces cerevisiae YJM789
0.005

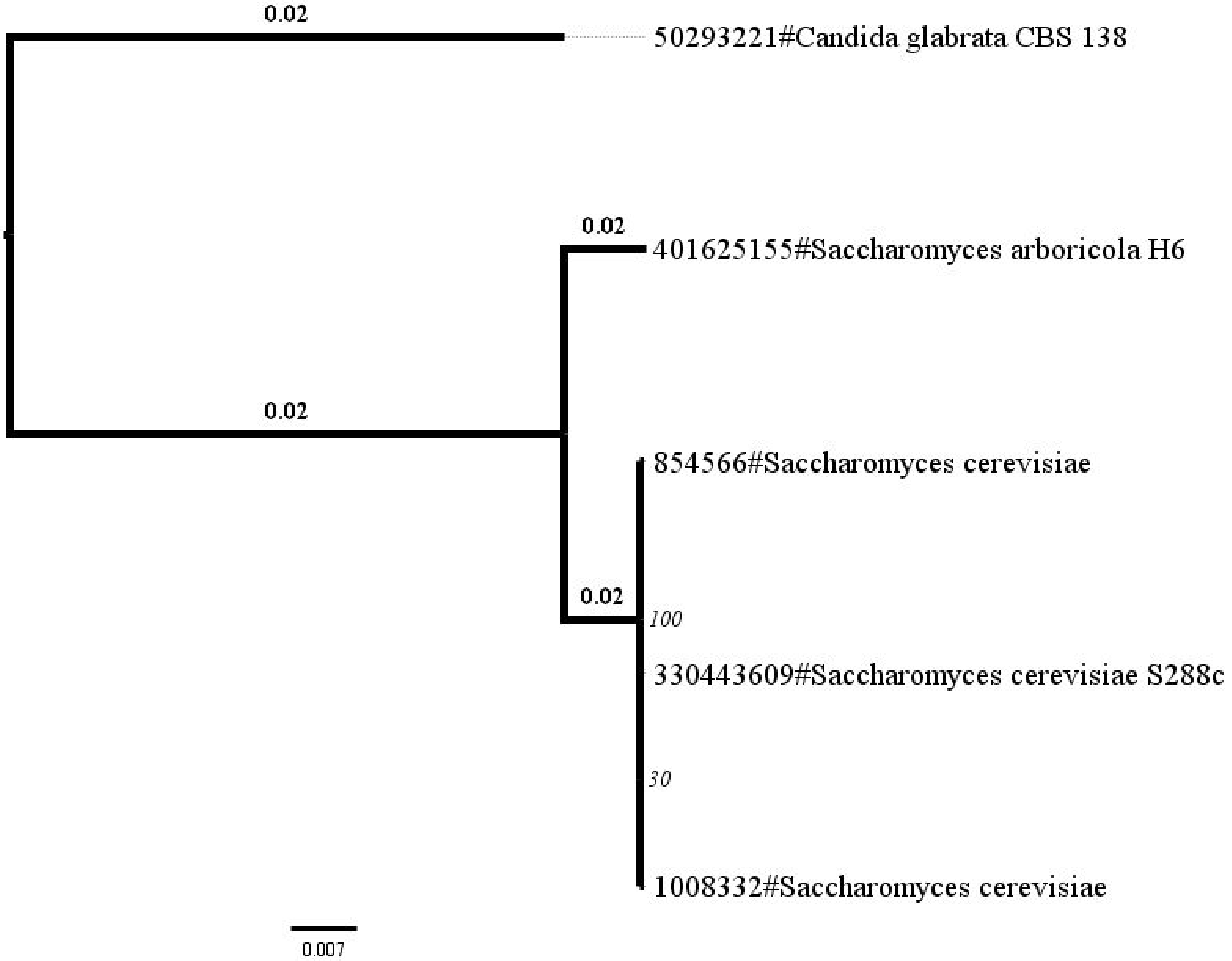
0.02
50293221#Candida glabrata CBS 138
0.02
401625155#Saccharomyces arboricola H6
0.02
854566#Saccharomyces cerevisiae
0.02
100
330443609#Saccharomyces cerevisiae S288c
30
1008332#Saccharomyces cerevisiae
0.007

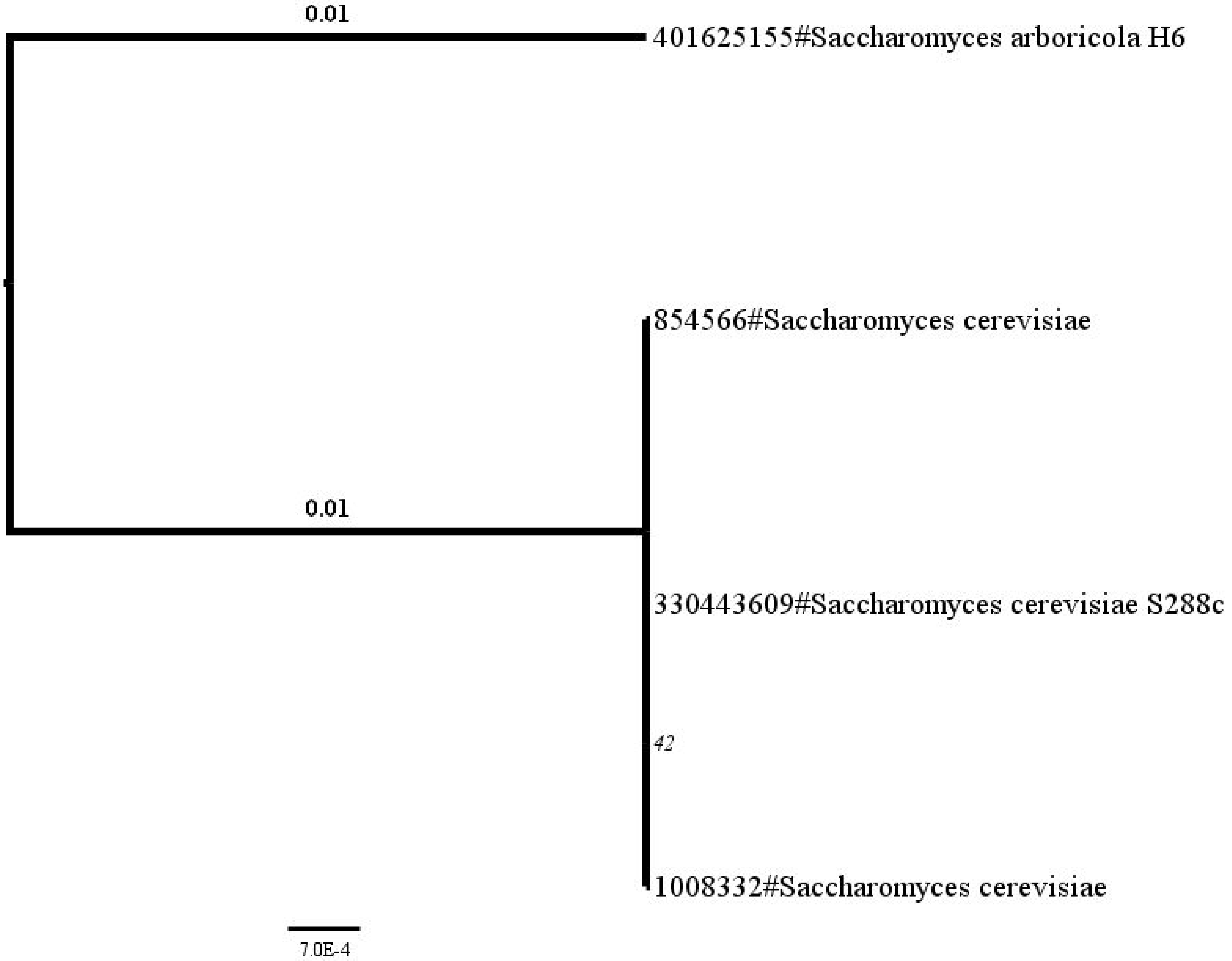
0.01
401625155#Saccharomyces arboricola H6
854566#Saccharomyces cerevisiae
0.01
330443609#Saccharomyces cerevisiae S288c
42
1008332#Saccharomyces cerevisiae
7.0E-4

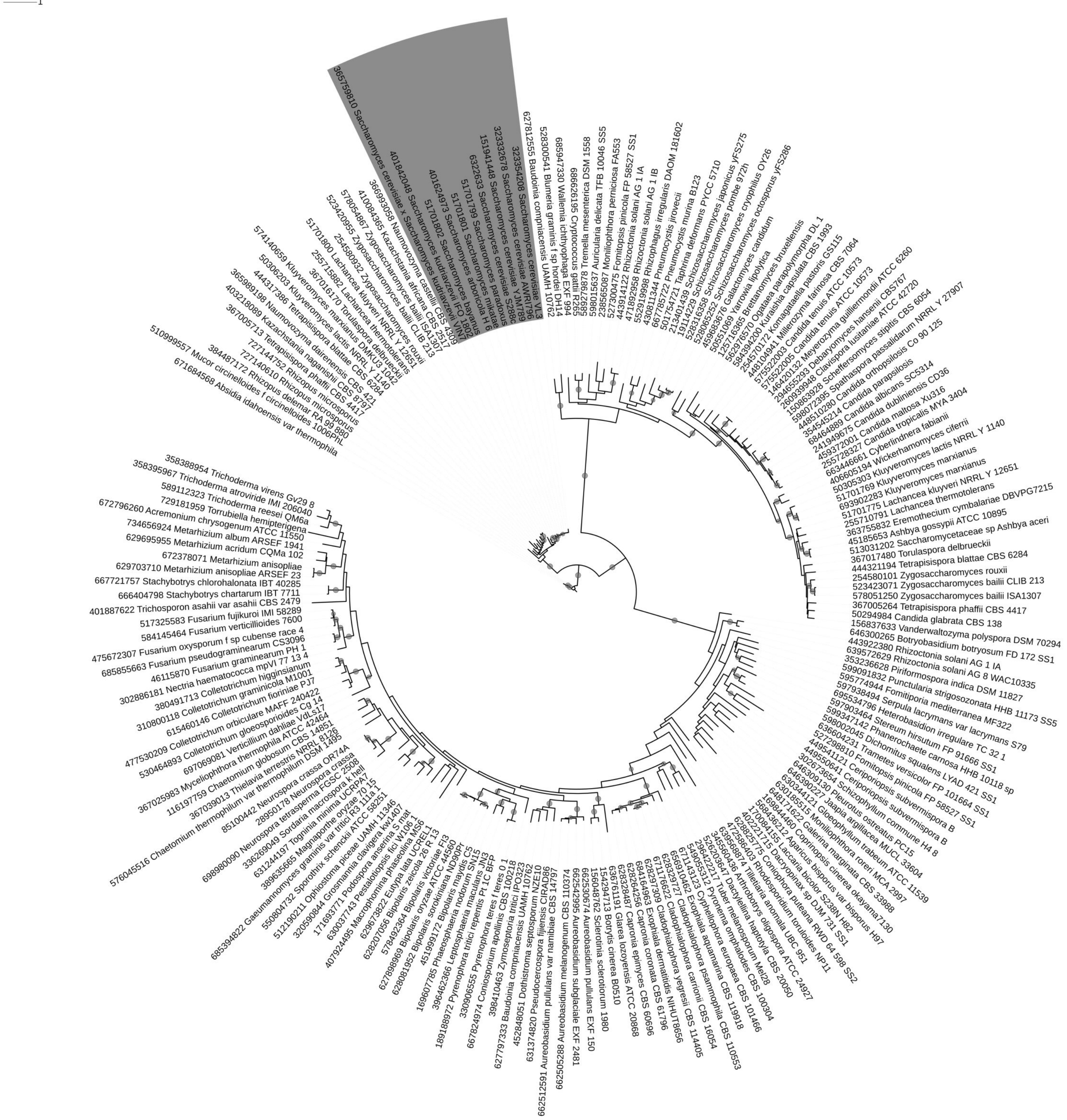
1
365759810 Saccharomyces cerevisiae x Saccharomyces kudriavzevii VIN7
401842048 Saccharomyces arboricola H 6
401624973 Saccharomyces kudriavzevii IFO 1802
51701802 Saccharomyces bayanus
51701801 Saccharomyces mikatae
51701799 Saccharomyces paradoxus
6322633 Saccharomyces cerevisiae S288c
151941448 Saccharomyces cerevisiae YJM789
323332678 Saccharomyces cerevisiae AWRI796
323354208 Saccharomyces cerevisiae VL3
627812555 Baudoinia compniacensis UAMH 10762
528300541 Blumeria graminis f sp hordei DH14
685947330 Wallemia ichthyophaga EXF 994
686626195 Cryptococcus gattii R265
589279878 Tremella mesenterica DSM 1558
598015637 Auricularia delicata TFB 10046 SS5
238565087 Moniliophthora perniciosa FA553
527300475 Fomitopsis pinicola FP 58527 SS1
443914122 Rhizoctonia solani AG 1 IA
471892958 Rhizoctonia solani AG 1 IB
552919998 Rhizophagus irregularis DAOM 181602
430811344 Pneumocystis murina B123
667785722 Pneumocystis jirovecii
501754721 Taphrina deformans PYCC 5710
213401439 Schizosaccharomyces japonicus yFS275
19114229 Schizosaccharomyces pombe 972h
528316358 Schizosaccharomyces cryophilus OY26
528065252 Schizosaccharomyces octosporus yFS286
459683676 Galactomyces candidum
505551069 Yarrowia lipolytica
12276365 Brettanomyces bruxellensis
562976570 Ogataea parapolymorpha DL 1
584394200 Kuraishia capsulata CBS 1993
254570172 Komagataella pastoris GS115
448104941 Millerozyma farinosa CBS 7064
575522003 Candida tenuis ATCC 10573
146420132 Meyerozyma guilliermondii ATCC 6260
294655293 Debaryomyces hansenii CBS767
260939948 Clavispora lusitaniae ATCC 42720
150863928 Scheffersomyces stipitis CBS 6054
598072395 Spathaspora passalidarum NRRL Y 27907
448510280 Candida orthopsilosis Co 90 125
354545214 Candida parapsilosis
68464889 Candida albicans SC5314
241949675 Candida dubliniensis CD36
255728327 Candida maltosa Xu316
459372001 Candida tropicalis MYA 3404
663446661 Cyberlindnera fabianii
406605194 Wickerhamomyces ciferrii
50305303 Kluyveromyces lactis NRRL Y 1140
51701769 Kluyveromyces marxianus
693902283 Kluyveromyces marxianus
51701775 Lachancea kluyveri NRRL Y 12651
255710791 Lachancea thermotolerans
363755832 Eremothecium cymbalariae DBVPG7215
45185653 Ashbya gossypii ATCC 10895
513031202 Saccharomycetaceae sp Ashbya aceri
367017480 Torulaspora delbrueckii
443321194 Tetrapisispora blattae CBS 6284
254580101 Zygosaccharomyces rouxii
523423071 Zygosaccharomyces bailii CLIB 213
578051250 Zygosaccharomyces bailii ISA1307
367005264 Tetrapisispora phaffii CBS 4417
50294984 Candida glabrata CBS 138
156837633 Vanderwaltozyma polyspora DSM 70294
646300265 Botryobasidium botryosum FD 172 SS1
443922380 Rhizoctonia solani AG 1 IA
639572629 Rhizoctonia solani AG 8 WAC10335
353236628 Piriformospora indica DSM 11827
599091832 Punctularia strigosozonata HHB 11173 SS5
595774944 Fomitiporia mediterranea MF322
597938494 Serpula lacrymans var lacrymans S79
695534796 Heterobasidion irregulare TC 32 1
597903464 Stereum hirsutum FP 91666 SS1
599347142 Phanerochaete carnosa HHB 10118 sp
598002045 Dichomitus squalens LYAD 421 SS1
636604231 Trametes versicolor FP 101664 SS1
527298810 Fomitopsis pinicola FP 58527 SS1
449541121 Ceriporiopsis subvermispora B
449550641 Ceriporiopsis subvermispora B
302673654 Schizophyllum commune H4 8
646309130 Pleurotus ostreatus PC15
646390227 Jaapia argillacea MUCL 33604
630308515 Gloeophyllum trabeum ATCC 11539
630344121 Moniliophthora roreri MCA 2997
648171622 Galerina marginata CBS 33988
169844460 Coprinopsis cinerea okayama7130
588436212 Agaricus bisporus var bisporus H97
170084155 Laccaria bicolor S238N H82
402216715 Dacryopinax sp DJM 731 SS1
628825737 Coniophora puteana RWD 64 598 SS2
472586403 Rhodosporidium toruloides NP11
636585674 Tilletiaria anomala UBC 951
345560174 Arthrobotrys oligospora ATCC 24927
295620367 Dactylellina haptotyla CBS 20050
564522181 Tuber melanosporum Mel28
549055312 Pyronema omphalodes CBS 100304
671113123 Exophiala aquamarina CBS 119918
656910462 Cladophialophora psammophila CBS 110553
626329727 Cyphellophora europaea CBS 101466
671176622 Cladophialophora yegresii CBS 114405
628297309 Cladophialophora carrionii CBS 16054
684164963 Exophiala dermatitidis NIHUT8656
628264229 Capronia coronata CBS 61796
628328487 Capronia epimyces CBS 60696
636761191 Glarea lozoyensis ATCC 20868
154294713 Botrytis cinerea B0510
156048762 Sclerotinia sclerotiorum 1980
662530674 Aureobasidium pullulans EXF 150
662542995 Aureobasidium subglaciale EXF 2481
662505288 Aureobasidium melanogenum CBS 110374
662512591 Aureobasidium pullulans var namibiae CBS 14797
631374820 Pseudocercospora fijiensis CIRAD86
452848051 Dothistroma septosporum NZE10
627797333 Baudoinia compniacensis UAMH 10762
398410463 Zymoseptoria tritici IPO323
667824974 Coniosporium apollinis CBS 100218
330906555 Pyrenophora teres f teres 0 1
189188972 Pyrenophora tritici repentis Pt 1C BFP
396462366 Leptosphaeria maculans JN3
169607785 Phaeosphaeria nodorum SN15
451999172 Bipolaris maydis C5
628081952 Bipolaris sorokiniana ND90Pr
627898969 Bipolaris oryzae ATCC 44560
578492364 Bipolaris victoriae FI3
628207056 Bipolaris zeicola 26 R 13
629673822 Eutypa lata UCREL1
407924495 Macrophomina phaseolina MS6
630037743 Pestalotiopsis fici W106 1
171693771 Podospora anserina S mat
320590844 Grosmannia clavigera kw1407
512190211 Ophiostoma piceae UAMH 11346
550804732 Sporothrix schenckii ATCC 58251
685394822 Gaeumannomyces graminis var tritici R3 111a 1
389635665 Magnaporthe oryzae 70 15
631244197 Togninia minima UCRPA7
336269049 Sordaria macrospora k hell
698980090 Neurospora tetrasperma FGSC 2508
85100442 Neurospora crassa OR74A
28950178 Neurospora crassa
576045516 Chaetomium thermophilum var thermophilum DSM 1495
367039013 Thielavia terrestris NRRL 8126
116197759 Chaetomium globosum CBS 14851
367025983 Myceliophthora thermophila ATCC 42464
530464893 Verticillium dahliae VdLs17
697069081 Colletotrichum gloeosporioides Cg 14
477530209 Colletotrichum orbiculare MAFF 240422
615460146 Colletotrichum fioriniae PJ7
310800118 Colletotrichum graminicola M1001
380491713 Colletotrichum higginsianum
302886181 Nectria haematococca mpVI 77 13 4
46115870 Fusarium graminearum PH 1
685855663 Fusarium pseudograminearum CS3096
475672307 Fusarium oxysporum f sp cubense race 4
584145464 Fusarium verticillioides 7600
517325583 Fusarium fujikuroi IMI 58289
401887622 Trichosporon asahii var asahii CBS 2479
666404798 Stachybotrys chartarum IBT 7711
667721757 Stachybotrys chlorohalonata IBT 40285
629703710 Metarhizium anisopliae ARSEF 23
672378071 Metarhizium anisopliae
629695955 Metarhizium acridum CQMa 102
734656924 Metarhizium album ARSEF 1941
672796260 Acremonium chrysogenum ATCC 11550
729181959 Torrubiella hemipterigena
589112323 Trichoderma reesei QM6a
358395967 Trichoderma atroviride IMI 206040
358388954 Trichoderma virens Gv29 8
671684568 Absidia idahoensis var thermophila
510999557 Mucor circinelloides f circinelloides 1006PhL
384487172 Rhizopus delemar RA 99 880
727140610 Rhizopus microsporus
727144752 Rhizopus microsporus
367005713 Tetrapisispora phaffii CBS 4417
403218089 Kazachstania naganishii CBS 8797
365989198 Naumovozyma dairenensis CBS 421
444317386 Tetrapisispora blattae CBS 6284
50306203 Kluyveromyces lactis NRRL Y 1140
574140659 Kluyveromyces marxianus DMKU3 1042
51701800 Lachancea kluyveri NRRL Y 12651
255715882 Lachancea thermotolerans
367016170 Torulaspora delbrueckii
254580932 Zygosaccharomyces rouxii
523420955 Zygosaccharomyces bailii CLIB 213
578054867 Zygosaccharomyces bailii ISA1307
410083365 Kazachstania africana CBS 2517
366993058 Naumovozyma castellii CBS 4309

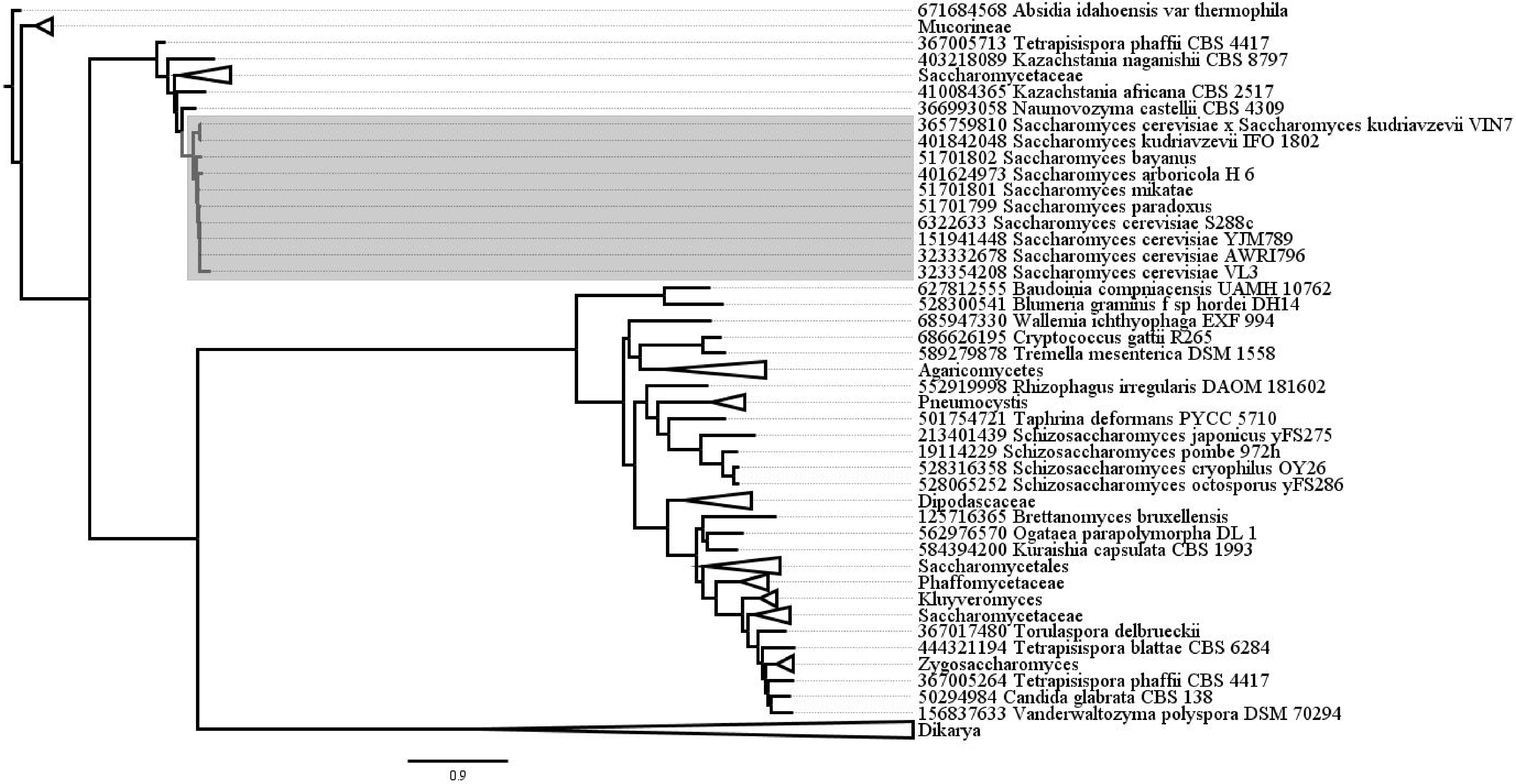
671684568 Absidia idahoensis var thermophila
Mucorineae
367005713 Tetrapisispora phaffii CBS 4417
403218089 Kazachstania naganishii CBS 8797
Saccharomycetaceae
410084365 Kazachstania africana CBS 2517
366993058 Naumovozyma castellii CBS 4309
365759810 Saccharomyces cerevisiae x Saccharomyces kudriavzevii VIN7
401842048 Saccharomyces kudriavzevii IFO 1802
51701802 Saccharomyces bayanus
401624973 Saccharomyces arboricola H 6
51701801 Saccharomyces mikatae
51701799 Saccharomyces paradoxus
6322633 Saccharomyces cerevisiae S288c
151941448 Saccharomyces cerevisiae YJM789
323332678 Saccharomyces cerevisiae AWRI796
323354208 Saccharomyces cerevisiae VL3
627812555 Baudoinia compniacensis UAMH 10762
528300541 Blumeria graminis f sp hordei DH14
685947330 Wallemia ichthyophaga EXF 994
686626195 Cryptococcus gattii R265
589279878 Tremella mesenterica DSM 1558
Agaricomycetes
552919998 Rhizophagus irregularis DAOM 181602
Pneumocystis
501754721 Taphrina deformans PYCC 5710
213401439 Schizosaccharomyces japonicus yFS275
19114229 Schizosaccharomyces pombe 972h
528316358 Schizosaccharomyces cryophilus OY26
528065252 Schizosaccharomyces octosporus yFS286
Dipodascaceae
125716365 Brettanomyces bruxellensis
562976570 Ogataea parapolymorpha DL 1
584394200 Kuraishia capsulata CBS 1993
Saccharomycetales
Phaffomycetaceae
Kluyveromyces
Saccharomycetaceae
367017480 Torulaspora delbrueckii
444321194 Tetrapisispora blattae CBS 6284
Zygosaccharomyces
367005264 Tetrapisispora phaffii CBS 4417
50294984 Candida glabrata CBS 138
156837633 Vanderwaltozyma polyspora DSM 70294
Dikarya
0.9

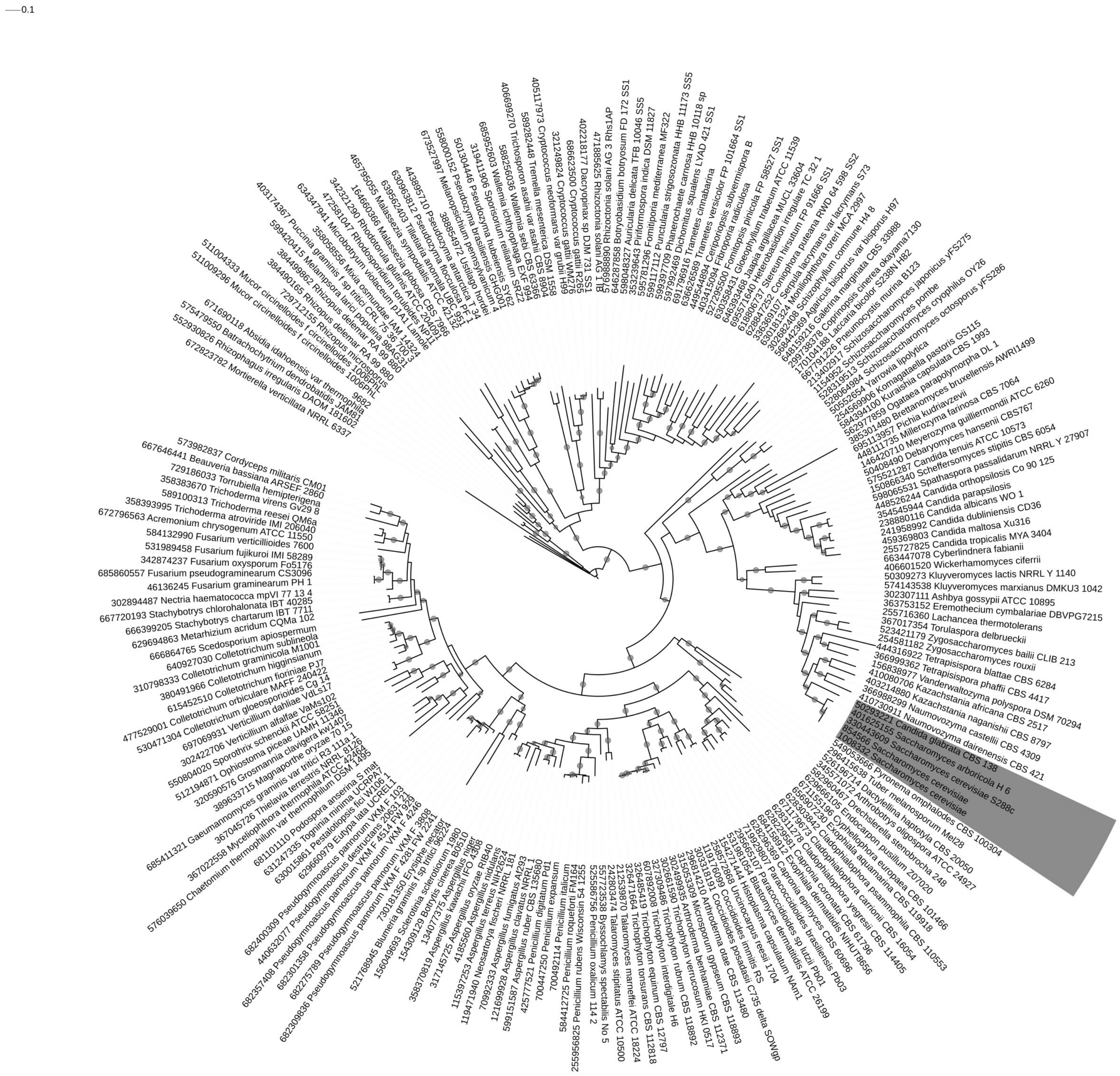
0.1

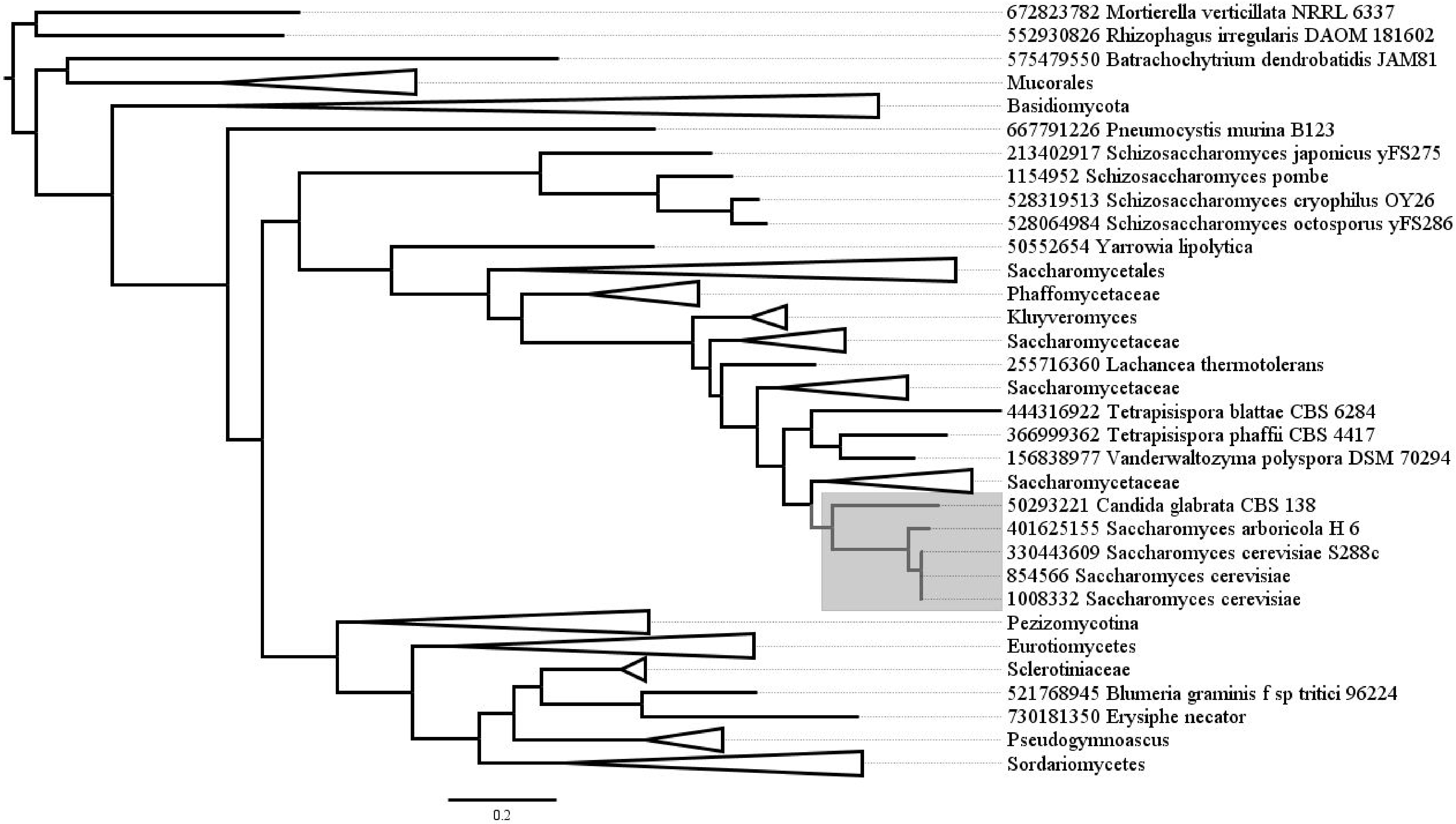
672823782 Mortierella verticillata NRRL 6337
552930826 Rhizophagus irregularis DAOM 181602
575479550 Batrachochytrium dendrobatidis JAM81
Mucorales
Basidiomycota
667791226 Pneumocystis murina B123
213402917 Schizosaccharomyces japonicus yFS275
1154952 Schizosaccharomyces pombe
528319513 Schizosaccharomyces cryophilus OY26
528064984 Schizosaccharomyces octosporus yFS286
50552654 Yarrowia lipolytica
Saccharomycetales
Phaffomycetaceae
Kluyveromyces
Saccharomycetaceae
255716360 Lachancea thermotolerans
Saccharomycetaceae
444316922 Tetrapisispora blattae CBS 6284
366999362 Tetrapisispora phaffii CBS 4417
156838977 Vanderwaltozyma polyspora DSM 70294
Saccharomycetaceae
50293221 Candida glabrata CBS 138
401625155 Saccharomyces arboricola H 6
330443609 Saccharomyces cerevisiae S288c
854566 Saccharomyces cerevisiae
1008332 Saccharomyces cerevisiae
Pezizomycotina
Eurotiomycetes
Sclerotiniaceae
521768945 Blumeria graminis f sp tritici 96224
730181350 Erysiphe necator
Pseudogymnoascus
Sordariomycetes
0.2

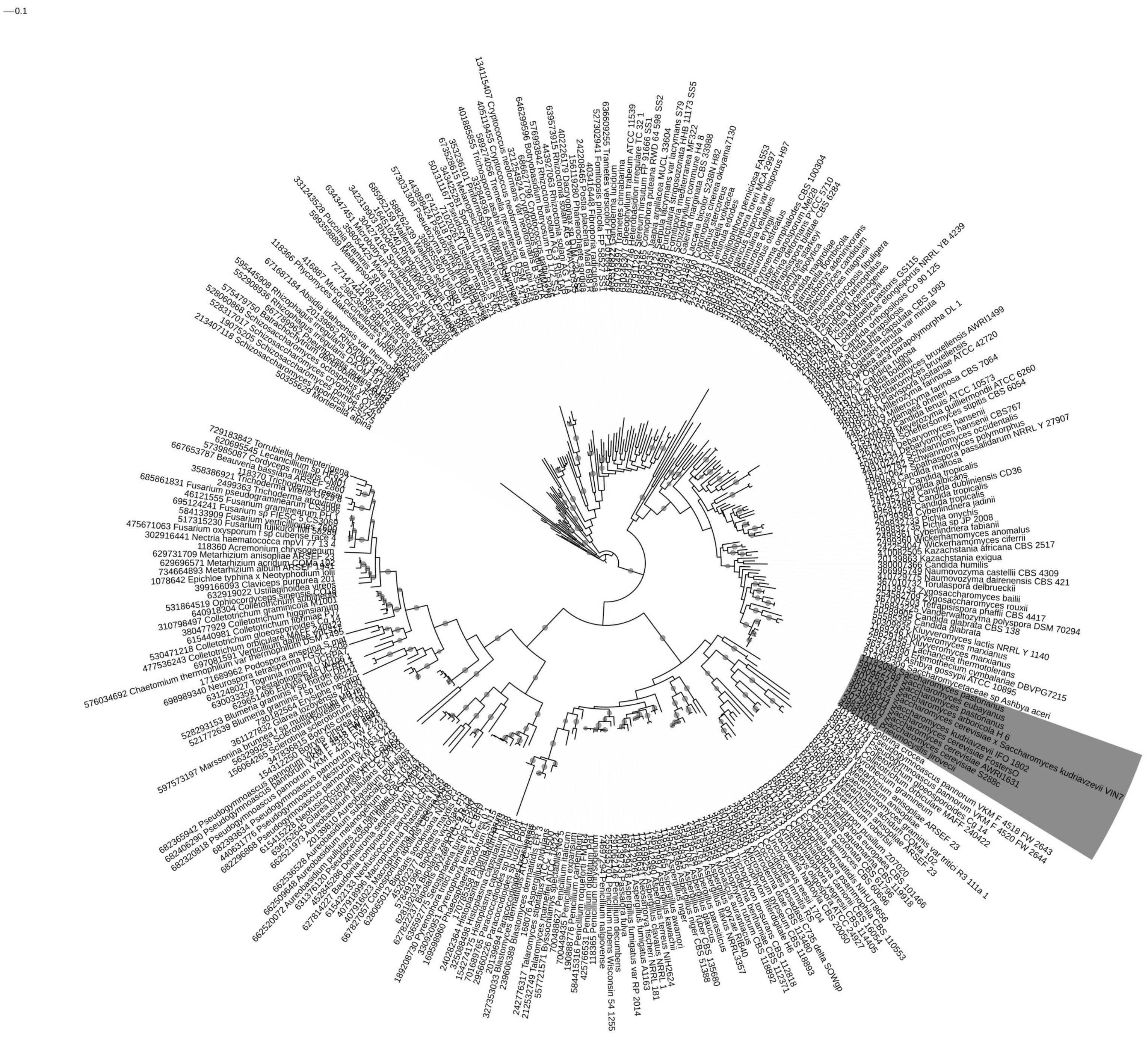
0.1

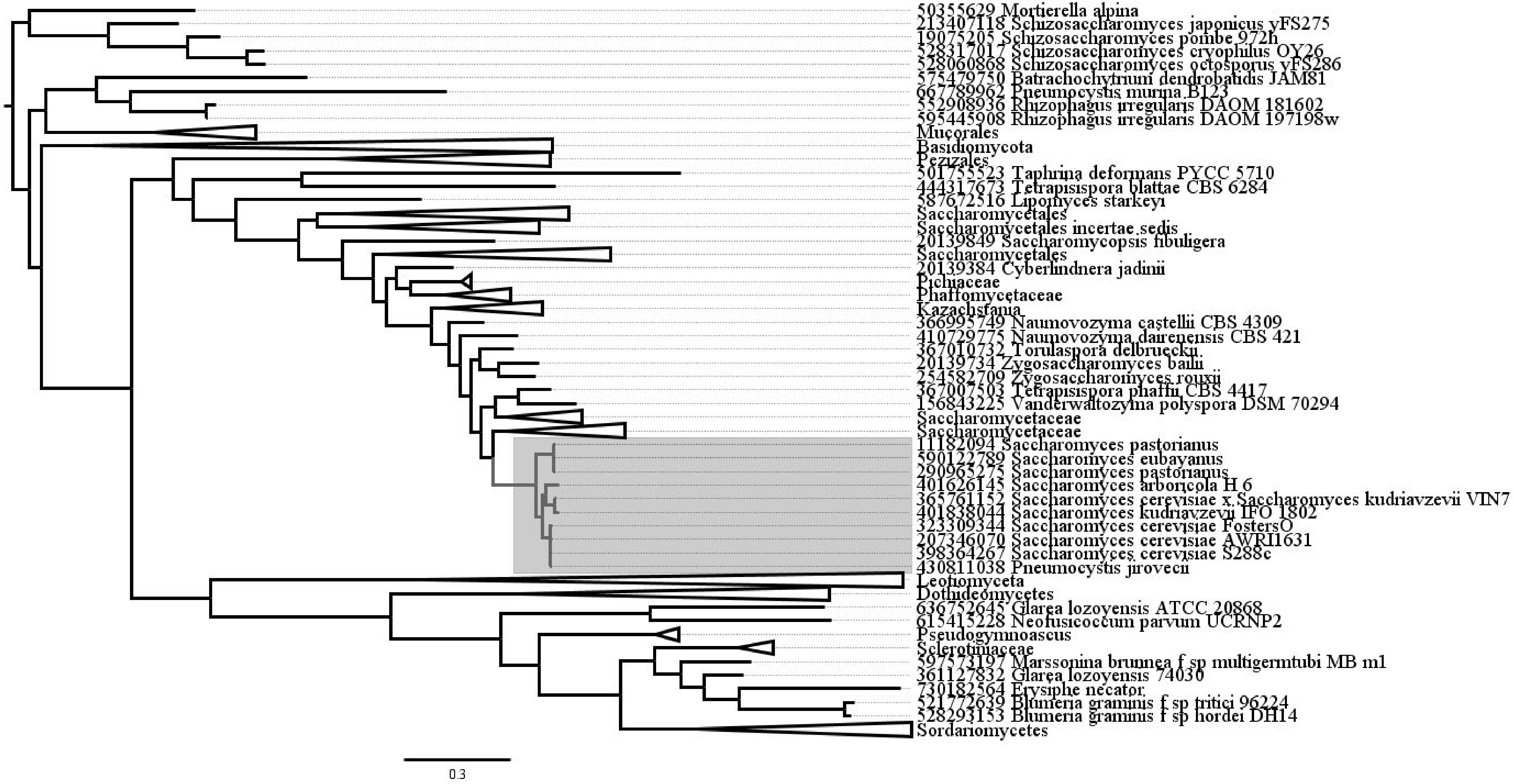
50355629 Mortierella alpina
213407118 Schizosaccharomyces japonicus yFS275
19075205 Schizosaccharomyces pombe 972h
528317017 Schizosaccharomyces cryophilus OY26
528060868 Schizosaccharomyces octosporus yFS286
575479750 Batrachochytrium dendrobatidis JAM81
667789962 Pneumocystis murina B123
552908936 Rhizophagus irregularis DAOM 181602
595445908 Rhizophagus irregularis DAOM 197198w
Mucorales
Basidiomycota
Pezizales
501755523 Taphrina deformans PYCC 5710
444317673 Tetrapisispora blattae CBS 6284
587672516 Lipomyces starkeyi
Saccharomycetales
Saccharomycetales incertae sedis
20139849 Saccharomycopsis fibuligera
Saccharomycetales
20139384 Cyberlindnera jadinii
Pichiaceae
Phaffomycetaceae
Kazachstania
366995749 Naumovozyma castellii CBS 4309
410729775 Naumovozyma dairenensis CBS 421
367010732 Torulaspora delbrueckii
20139734 Zygosaccharomyces bailii
254582709 Zygosaccharomyces rouxii
367007503 Tetrapisispora phaffii CBS 4417
156843225 Vanderwaltozyma polyspora DSM 70294
Saccharomycetaceae
Saccharomycetaceae
11182094 Saccharomyces pastorianus
590122789 Saccharomyces eubayanus
290965275 Saccharomyces pastorianus
401626145 Saccharomyces arboricola H 6
365761152 Saccharomyces cerevisiae x Saccharomyces kudriavzevii VIN7
401838044 Saccharomyces kudriavzevii IFO 1802
323309344 Saccharomyces cerevisiae FostersO
207346070 Saccharomyces cerevisiae AWRI1631
398364267 Saccharomyces cerevisiae S288c
430811038 Pneumocystis jirovecii
Leotiomyceta
Dothideomycetes
636752645 Glarea lozoyensis ATCC 20868
615415228 Neofusicoccum parvum UCRNP2
Pseudogymnoascus
Sclerotiniaceae
597573197 Marssonina brunnea f sp multigermtubi MB m1
361127832 Glarea lozoyensis 74030
730182564 Erysiphe necator
521772639 Blumeria graminis f sp tritici 96224
528293153 Blumeria graminis f sp hordei DH14
Sordariomycetes
0.3

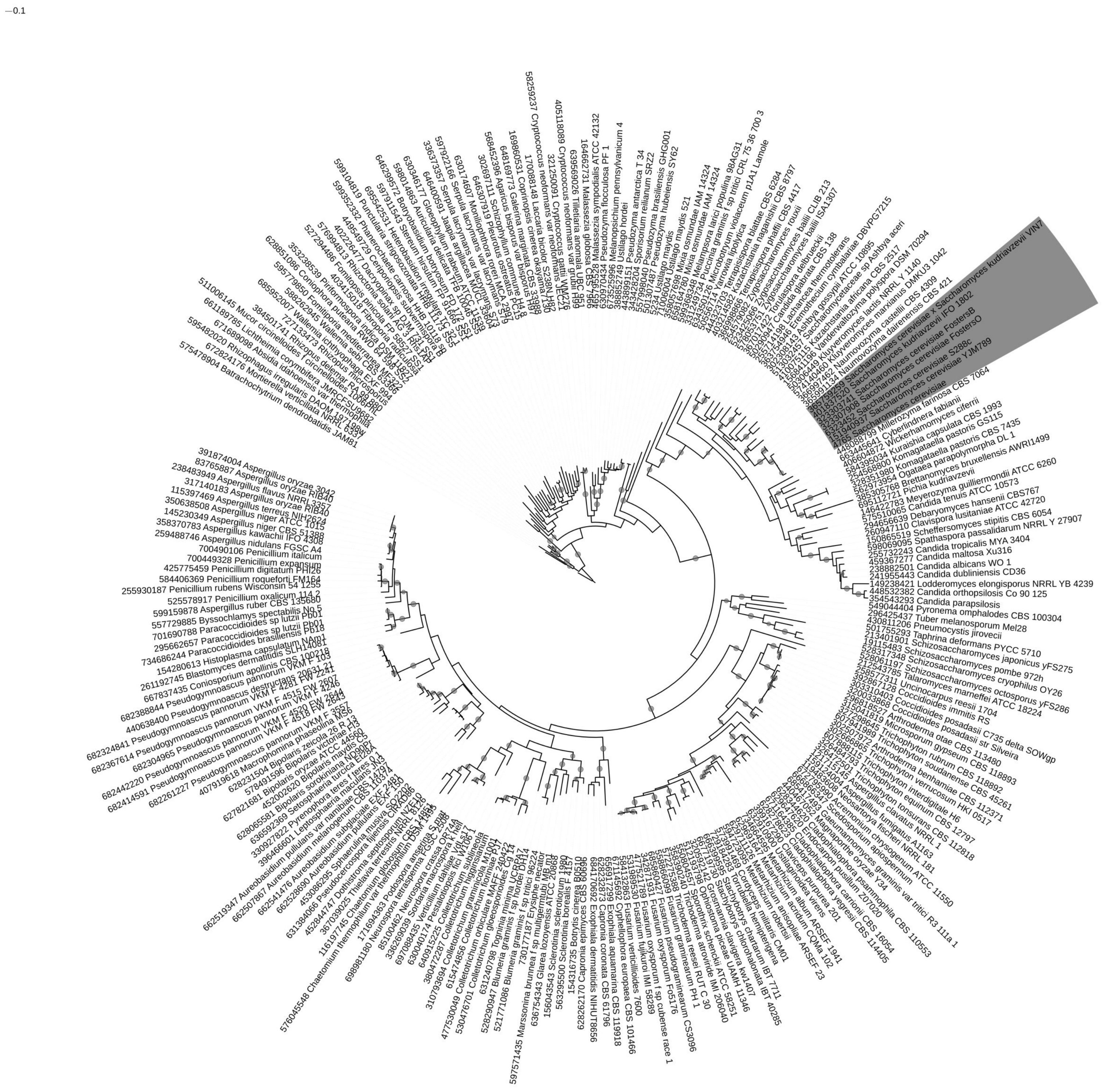
0.1
149238421 Lodderomyces elongisporus NRRL YB 4239
448532382 Candida orthopsilosis Co 90 125
354543293 Candida parapsilosis
549044404 Pyronema omphalodes CBS 100304
296425437 Tuber melanosporum Mel28
430811206 Pneumocystis jirovecii
501755293 Taphrina deformans PYCC 5710
238882501 Candida albicans WO 1
241955443 Candida dubliniensis CD36
255732243 Candida tropicalis MYA 3404
598069095 Spathaspora passalidarum NRRL Y 27907
391874004 Aspergillus oryzae 3042
83765887 Aspergillus oryzae RIB40
238483949 Aspergillus flavus NRRL3357
259488746 Aspergillus nidulans FGSC A4
700490106 Penicillium italicum
700449328 Penicillium expansum
425775459 Penicillium digitatum PHI26
584406369 Penicillium roqueforti FM164
255930187 Penicillium rubens Wisconsin 54 1255
525578917 Penicillium oxalicum 114 2
599159878 Aspergillus ruber CBS 135680
557729885 Byssochlamys spectabilis No 5
701690788 Paracoccidioides sp lutzii Pb01
295662657 Paracoccidioides brasiliensis Pb18
734686244 Paracoccidioides brasiliensis PbAm1
154280613 Histoplasma capsulatum NAm1
261192745 Blastomyces dermatitidis SLH14081
575478904 Batrachochytrium dendrobatidis JAM81
672824176 Mortierella verticillata NRRL 6337

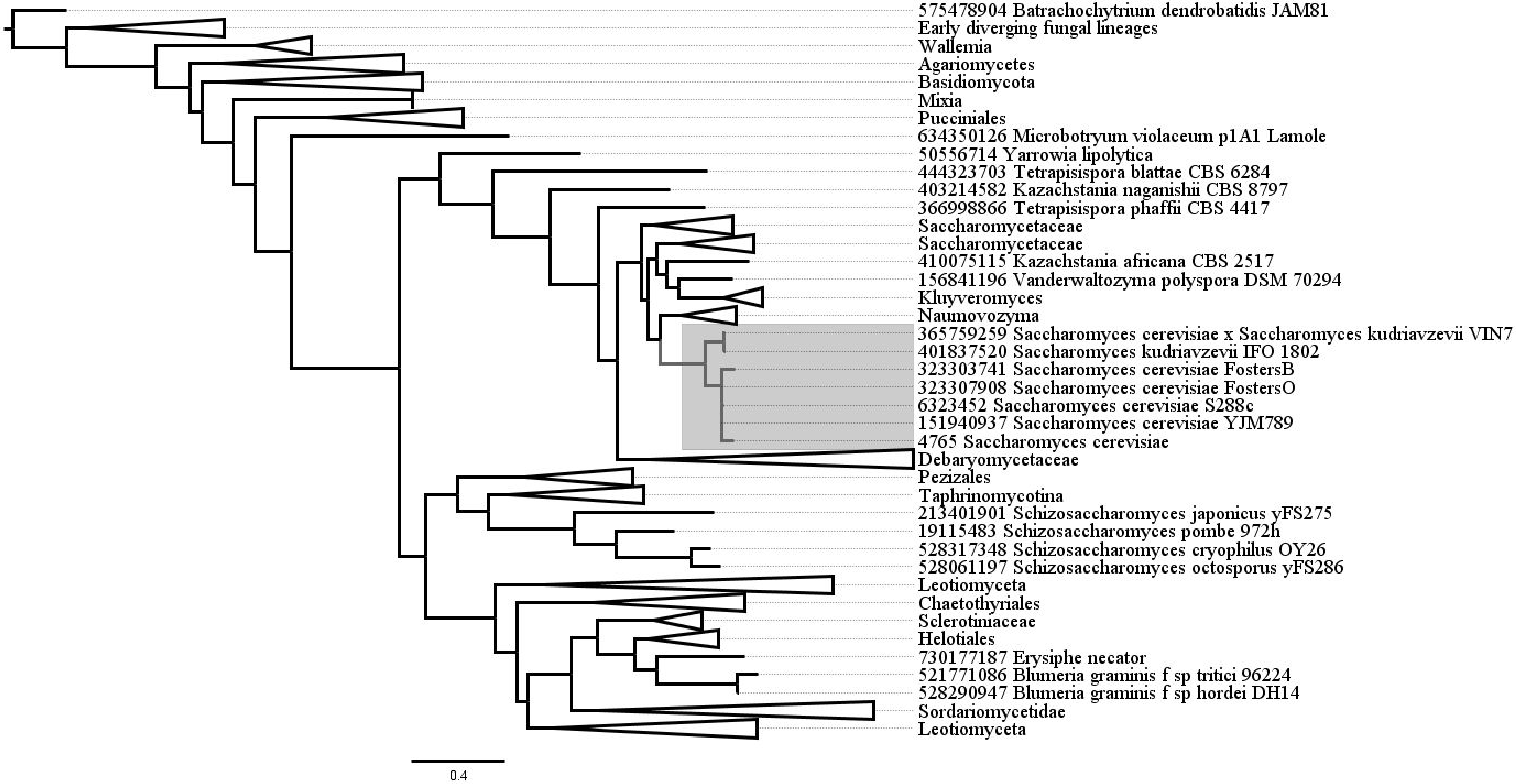
575478904 Batrachochytrium dendrobatidis JAM81
Early diverging fungal lineages
Wallemia
Agariomycetes
Basidiomycota
Mixia
Pucciniales
634350126 Microbotryum violaceum p1A1 Lamole
50556714 Yarrowia lipolytica
444323703 Tetrapisispora blattae CBS 6284
403214582 Kazachstania naganishii CBS 8797
366998866 Tetrapisispora phaffii CBS 4417
Saccharomycetaceae
Saccharomycetaceae
410075115 Kazachstania africana CBS 2517
156841196 Vanderwaltozyma polyspora DSM 70294
Kluyveromyces
Naumovozyma
365759259 Saccharomyces cerevisiae x Saccharomyces kudriavzevii VIN7
401837520 Saccharomyces kudriavzevii IFO 1802
323303741 Saccharomyces cerevisiae FostersB
323307908 Saccharomyces cerevisiae FostersO
6323452 Saccharomyces cerevisiae S288c
151940937 Saccharomyces cerevisiae YJM789
4765 Saccharomyces cerevisiae
Debaryomycetaceae
Pezizales
Taphrinomycotina
213401901 Schizosaccharomyces japonicus yFS275
19115483 Schizosaccharomyces pombe 972h
528317348 Schizosaccharomyces cryophilus OY26
528061197 Schizosaccharomyces octosporus yFS286
Leotiomyceta
Chaetothyriales
Sclerotiniaceae
Helotiales
730177187 Erysiphe necator
521771086 Blumeria graminis f sp tritici 96224
528290947 Blumeria graminis f sp hordei DH14
Sordariomycetidae
Leotiomyceta
0.4

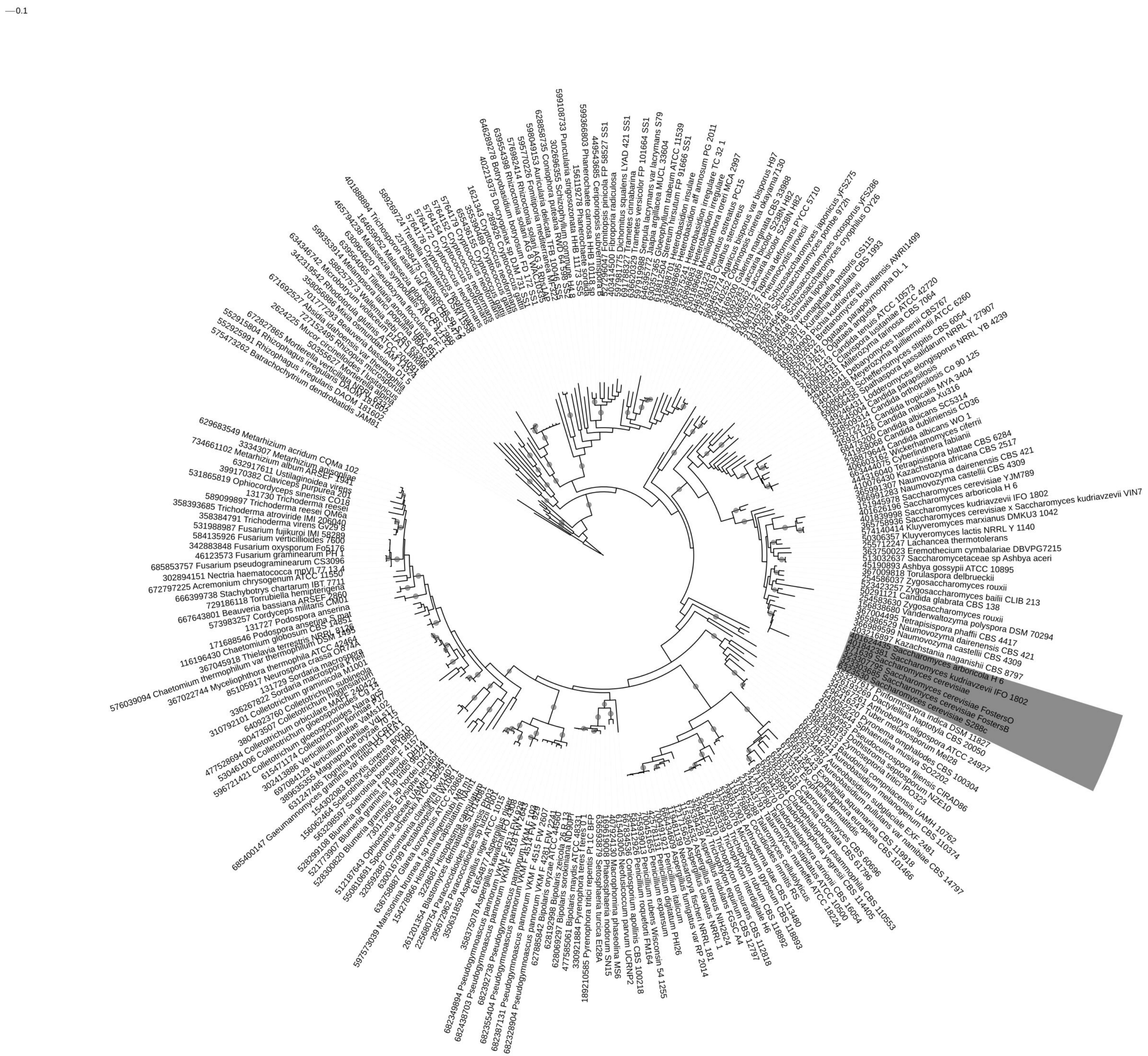
0.1

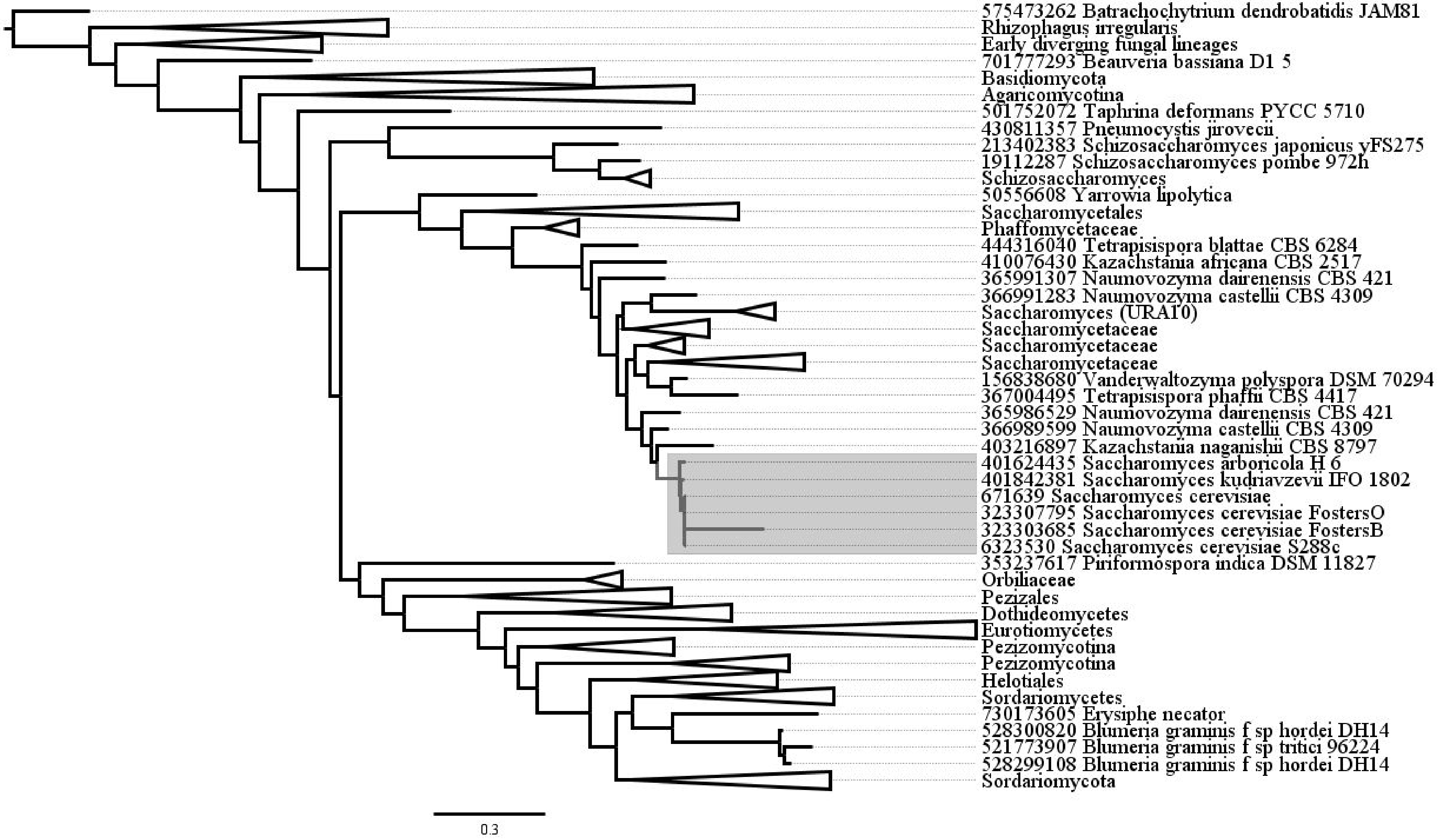
575473262 Batrachochytrium dendrobatidis JAM81
Rhizophagus irregularis
Early diverging fungal lineages
701777293 Beauveria bassiana D1 5
Basidiomycota
Agaricomycotina
501752072 Taphrina deformans PYCC 5710
430811357 Pneumocystis jirovecii
213402383 Schizosaccharomyces japonicus yFS275
19112287 Schizosaccharomyces pombe 972h
Schizosaccharomyces
50556608 Yarrowia lipolytica
Saccharomycetales
Phaffomycetaceae
444316040 Tetrapisispora blattae CBS 6284
410076430 Kazachstania africana CBS 2517
365991307 Naumovozyma dairenensis CBS 421
366991283 Naumovozyma castellii CBS 4309
Saccharomyces (URA10)
Saccharomycetaceae
Saccharomycetaceae
Saccharomycetaceae
156838680 Vanderwaltozyma polyspora DSM 70294
367004495 Tetrapisispora phaffii CBS 4417
365986529 Naumovozyma dairenensis CBS 421
366989599 Naumovozyma castellii CBS 4309
403216897 Kazachstania naganishii CBS 8797
401624435 Saccharomyces arboricola H 6
401842381 Saccharomyces kudriavzevii IFO 1802
671639 Saccharomyces cerevisiae
323307795 Saccharomyces cerevisiae FostersO
323303685 Saccharomyces cerevisiae FostersB
6323530 Saccharomyces cerevisiae S288c
353237617 Piriformospora indica DSM 11827
Orbiliaceae
Pezizales
Dothideomycetes
Eurotiomycetes
Pezizomycotina
Pezizomycotina
Helotiales
Sordariomycetes
730173605 Erysiphe necator
528300820 Blumeria graminis f sp hordei DH14
521773907 Blumeria graminis f sp tritici 96224
528299108 Blumeria graminis f sp hordei DH14
Sordariomycota
0.3

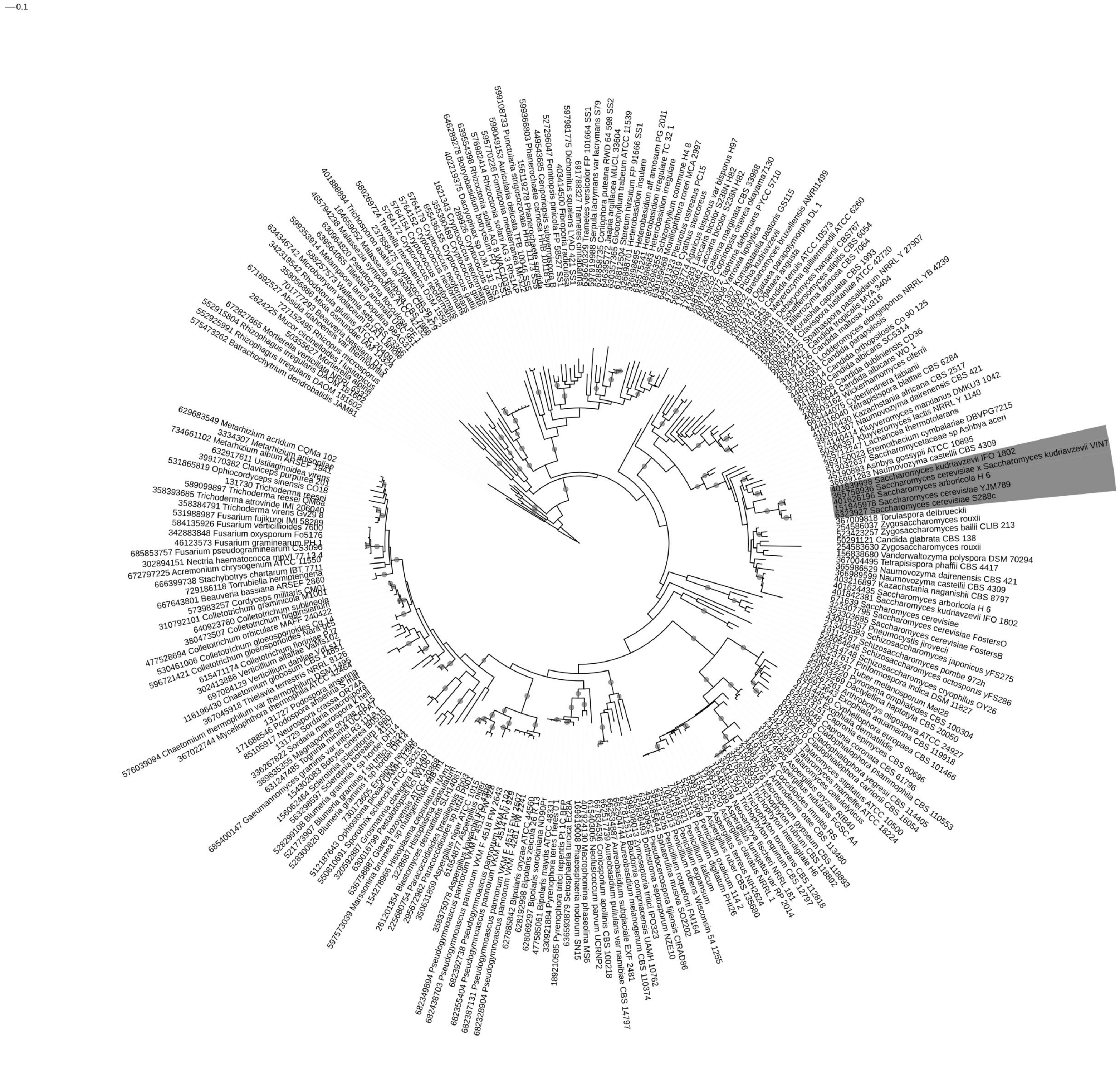
0.1

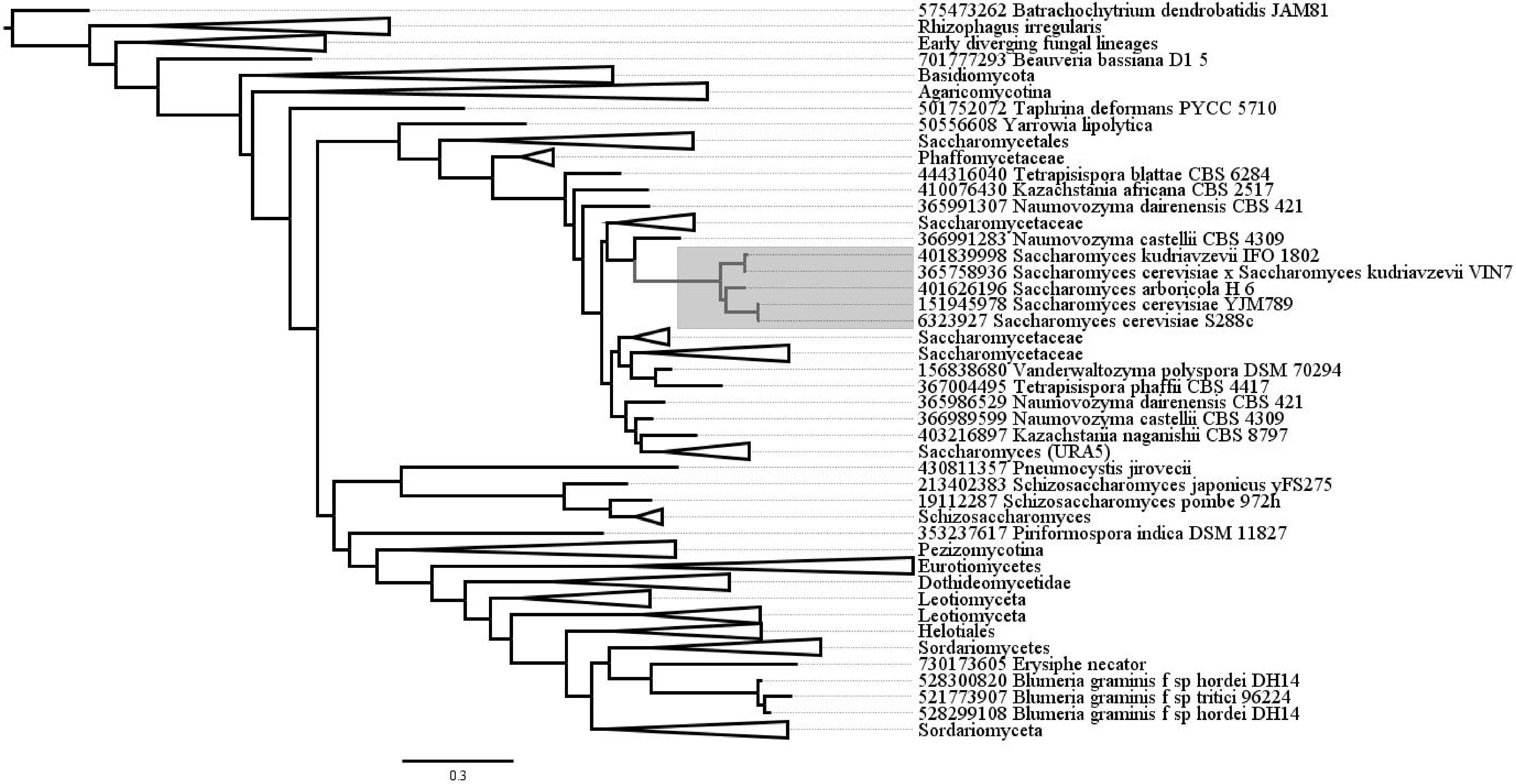
575473262 Batrachochytrium dendrobatidis JAM81
Rhizophagus irregularis
Early diverging fungal lineages
701777293 Beauveria bassiana D1 5
Basidiomycota
Agaricomycotina
501752072 Taphrina deformans PYCC 5710
50556608 Yarrowia lipolytica
Saccharomycetales
Phaffomycetaceae
444316040 Tetrapisispora blattae CBS 6284
410076430 Kazachstania africana CBS 2517
365991307 Naumovozyma dairenensis CBS 421
Saccharomycetaceae
366991283 Naumovozyma castellii CBS 4309
401839998 Saccharomyces kudriavzevii IFO 1802
365758936 Saccharomyces cerevisiae x Saccharomyces kudriavzevii VIN7
401626196 Saccharomyces arboricola H 6
151945978 Saccharomyces cerevisiae YJM789
6323927 Saccharomyces cerevisiae S288c
Saccharomycetaceae
Saccharomycetaceae
156838680 Vanderwaltozyma polyspora DSM 70294
367004495 Tetrapisispora phaffii CBS 4417
365986529 Naumovozyma dairenensis CBS 421
366989599 Naumovozyma castellii CBS 4309
403216897 Kazachstania naganishii CBS 8797
Saccharomyces (URA5)
430811357 Pneumocystis jirovecii
213402383 Schizosaccharomyces japonicus yFS275
19112287 Schizosaccharomyces pombe 972h
Schizosaccharomyces
353237617 Piriformospora indica DSM 11827
Pezizomycotina
Eurotiomycetes
Dothideomycetidae
Leotiomyceta
Leotiomyceta
Helotiales
Sordariomycetes
730173605 Erysiphe necator
528300820 Blumeria graminis f sp hordei DH14
521773907 Blumeria graminis f sp tritici 96224
528299108 Blumeria graminis f sp hordei DH14
Sordariomyceta
0.3

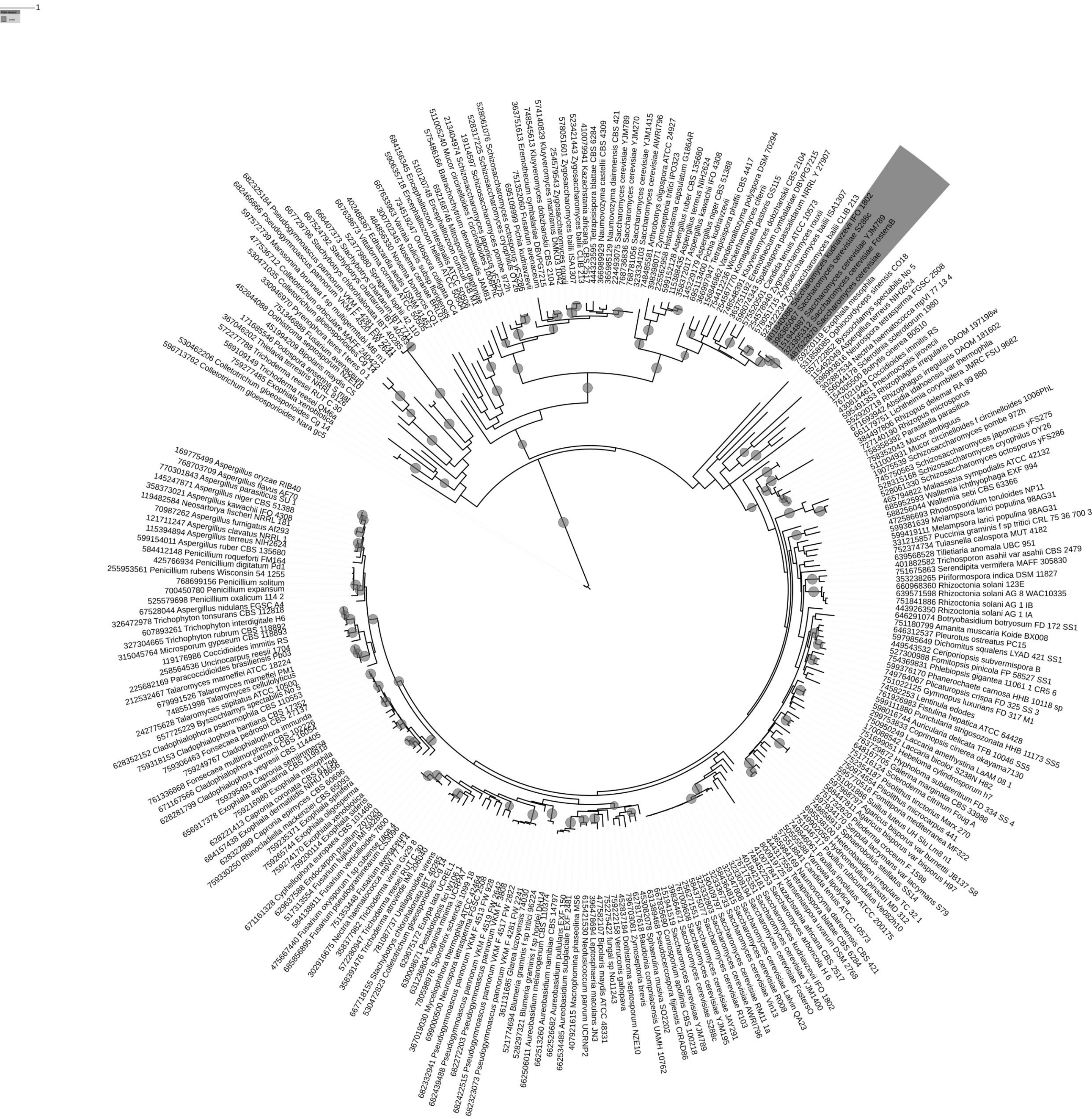
1

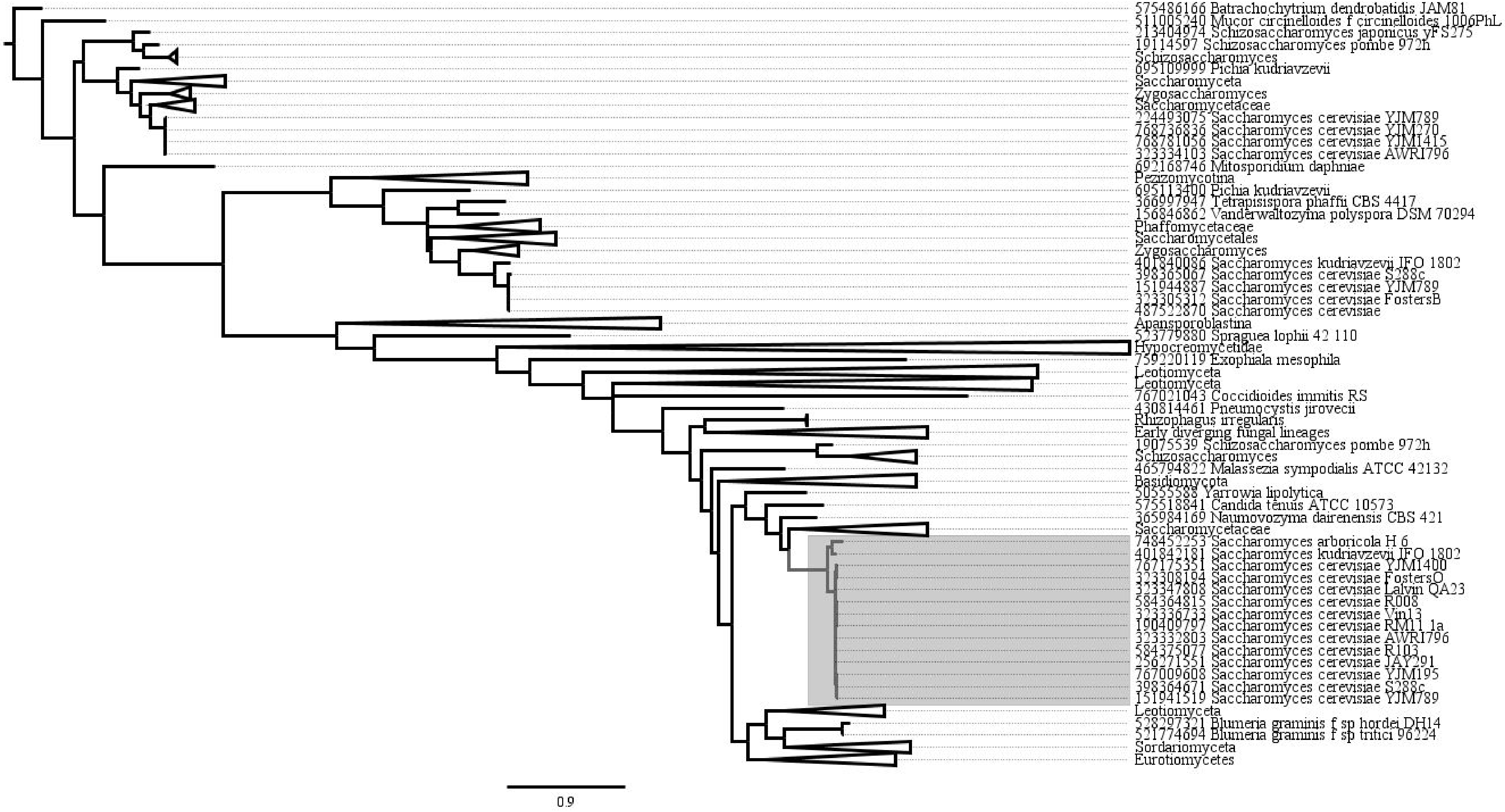
575486166 Batrachochytrium dendrobatidis JAM81
511005240 Mucor circinelloides f circinelloides 1006PhL
213404974 Schizosaccharomyces japonicus yFS275
19114597 Schizosaccharomyces pombe 972h
Schizosaccharomyces
695109999 Pichia kudriavzevii
Saccharomyceta
Zygosaccharomyces
Saccharomycetaceae
224493075 Saccharomyces cerevisiae YJM789
768736836 Saccharomyces cerevisiae YJM270
768781056 Saccharomyces cerevisiae YJM1415
323334103 Saccharomyces cerevisiae AWRI796
692168746 Mitosporidium daphniae
Pezizomycotina
695113400 Pichia kudriavzevii
366997947 Tetrapisispora phaffii CBS 4417
156846862 Vanderwaltozyma polyspora DSM 70294
Phaffomycetaceae
Saccharomycetales
Zygosaccharomyces
401840086 Saccharomyces kudriavzevii IFO 1802
398365067 Saccharomyces cerevisiae S288c
151944887 Saccharomyces cerevisiae YJM789
323305312 Saccharomyces cerevisiae FostersB
487522870 Saccharomyces cerevisiae
Apansporoblastina
523779880 Spraguea lophii 42 110
Hypocreomycetidae
759220119 Exophiala mesophila
Leotiomyceta
Leotiomyceta
767021043 Coccidioides immitis RS
430814461 Pneumocystis jirovecii
Rhizophagus irregularis
Early diverging fungal lineages
19075539 Schizosaccharomyces pombe 972h
Schizosaccharomyces
465794822 Malassezia sympodialis ATCC 42132
Basidiomycota
50555588 Yarrowia lipolytica
575518841 Candida tenuis ATCC 10573
365984169 Naumovozyma dairenensis CBS 421
Saccharomycetaceae
748452253 Saccharomyces arboricola H 6
401842181 Saccharomyces kudriavzevii IFO 1802
767175351 Saccharomyces cerevisiae YJM1400
323308194 Saccharomyces cerevisiae FostersO
323347808 Saccharomyces cerevisiae Lalvin QA23
584364815 Saccharomyces cerevisiae R008
323336733 Saccharomyces cerevisiae Vin13
190409797 Saccharomyces cerevisiae RM11 1a
323332803 Saccharomyces cerevisiae AWRI796
584375077 Saccharomyces cerevisiae R103
256271551 Saccharomyces cerevisiae JAY291
767009608 Saccharomyces cerevisiae YJM195
398364671 Saccharomyces cerevisiae S288c
151941519 Saccharomyces cerevisiae YJM789
Leotiomyceta
528297321 Blumeria graminis f sp hordei DH14
521774694 Blumeria graminis f sp tritici 96224
Sordariomyceta
Eurotiomycetes
0.9

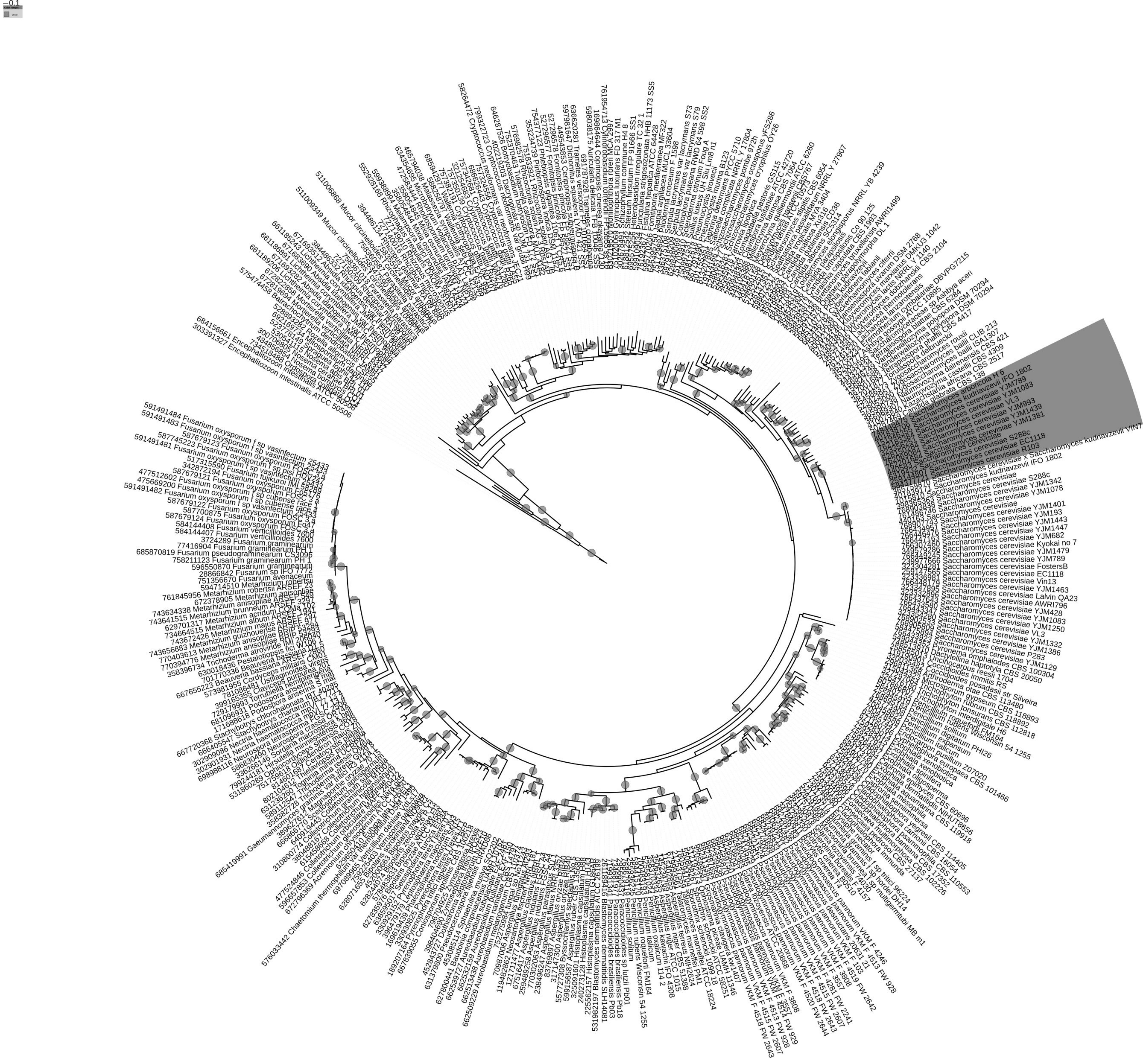

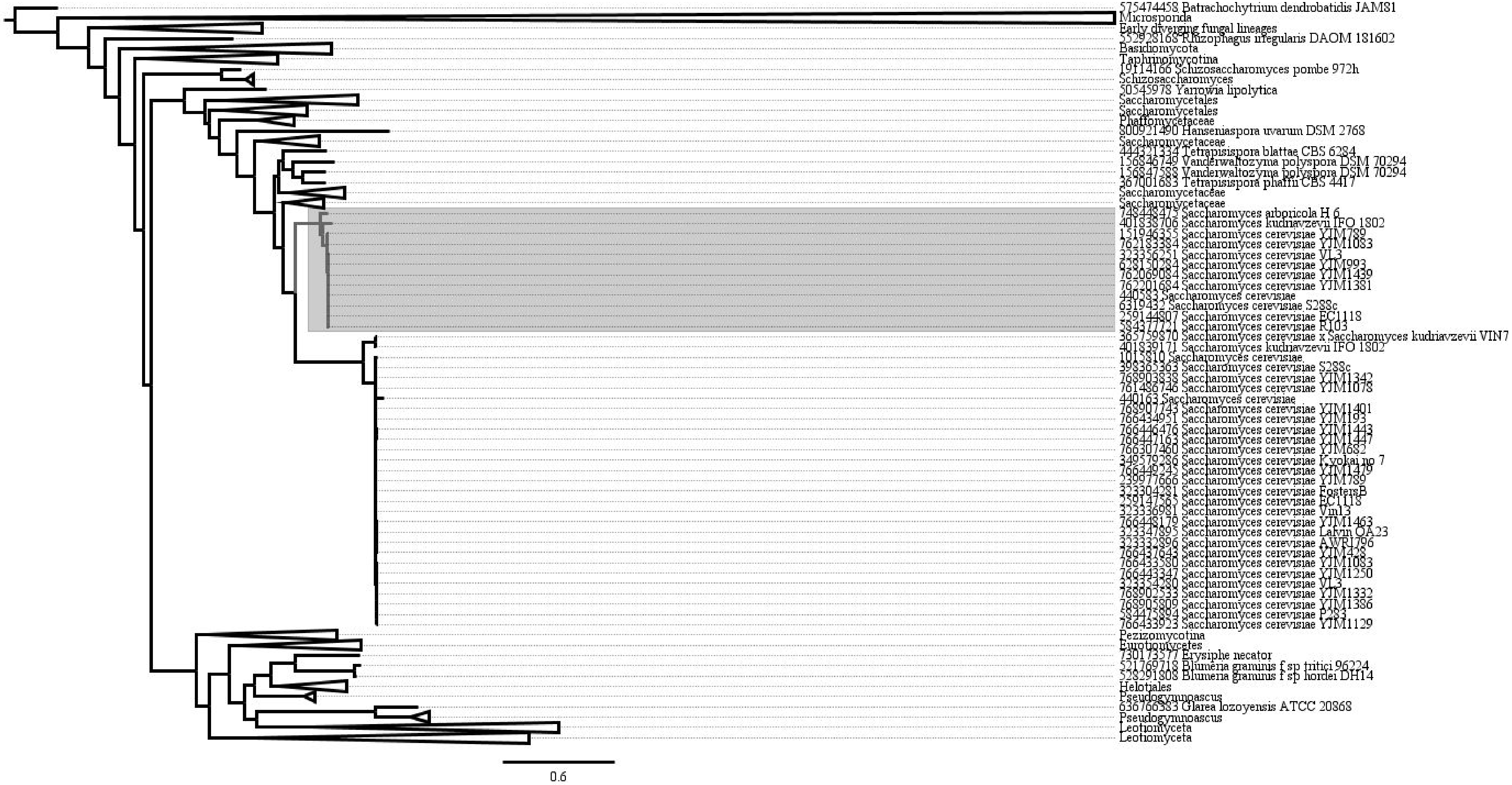
575474458 Batrachochytrium dendrobatidis JAM81
Microsporidia
Early diverging fungal lineages
552928168 Rhizophagus irregularis DAOM 181602
Basidiomycota
Taphrinomycotina
19114166 Schizosaccharomyces pombe 972h
Schizosaccharomyces
50545978 Yarrowia lipolytica
Saccharomycetales
Saccharomycetales
Phaffomycetaceae
800921490 Hanseniaspora uvarum DSM 2768
Saccharomycetaceae
444321334 Tetrapisispora blattae CBS 6284
156846749 Vanderwaltozyma polyspora DSM 70294
156847588 Vanderwaltozyma polyspora DSM 70294
367001683 Tetrapisispora phaffii CBS 4417
Saccharomycetaceae
Saccharomycetaceae
748448475 Saccharomyces arboricola H 6
401838706 Saccharomyces kudriavzevii IFO 1802
151946355 Saccharomyces cerevisiae YJM789
762183384 Saccharomyces cerevisiae YJM1083
323356251 Saccharomyces cerevisiae VL3
628150284 Saccharomyces cerevisiae YJM993
762069084 Saccharomyces cerevisiae YJM1439
762201684 Saccharomyces cerevisiae YJM1381
440583 Saccharomyces cerevisiae
6319432 Saccharomyces cerevisiae S288c
259144807 Saccharomyces cerevisiae EC1118
584377721 Saccharomyces cerevisiae R103
365759870 Saccharomyces cerevisiae x Saccharomyces kudriavzevii VIN7
401839171 Saccharomyces kudriavzevii IFO 1802
1015810 Saccharomyces cerevisiae
398365363 Saccharomyces cerevisiae S288c
768903838 Saccharomyces cerevisiae YJM1342
761486746 Saccharomyces cerevisiae YJM1078
440163 Saccharomyces cerevisiae
768907743 Saccharomyces cerevisiae YJM1401
766434951 Saccharomyces cerevisiae YJM193
766446476 Saccharomyces cerevisiae YJM1443
766447163 Saccharomyces cerevisiae YJM1447
766307460 Saccharomyces cerevisiae YJM682
349579286 Saccharomyces cerevisiae Kyokai no 7
766449245 Saccharomyces cerevisiae YJM1479
239977666 Saccharomyces cerevisiae YJM789
323304281 Saccharomyces cerevisiae FostersB
259147565 Saccharomyces cerevisiae EC1118
323336981 Saccharomyces cerevisiae Vin13
766448179 Saccharomyces cerevisiae YJM1463
323347895 Saccharomyces cerevisiae Lalvin QA23
323332896 Saccharomyces cerevisiae AWRI796
766437643 Saccharomyces cerevisiae YJM428
766433580 Saccharomyces cerevisiae YJM1083
766443347 Saccharomyces cerevisiae YJM1250
323354280 Saccharomyces cerevisiae VL3
768902533 Saccharomyces cerevisiae YJM1332
768905809 Saccharomyces cerevisiae YJM1386
584475894 Saccharomyces cerevisiae P283
766433923 Saccharomyces cerevisiae YJM1129
Pezizomycotina
Eurotiomycetes
730173577 Erysiphe necator
521769718 Blumeria graminis f sp tritici 96224
528291808 Blumeria graminis f sp hordei DH14
Helotiales
Pseudogymnoascus
636766383 Glarea lozoyensis ATCC 20868
Pseudogymnoascus
Leotiomyceta
Leotiomyceta
0.6

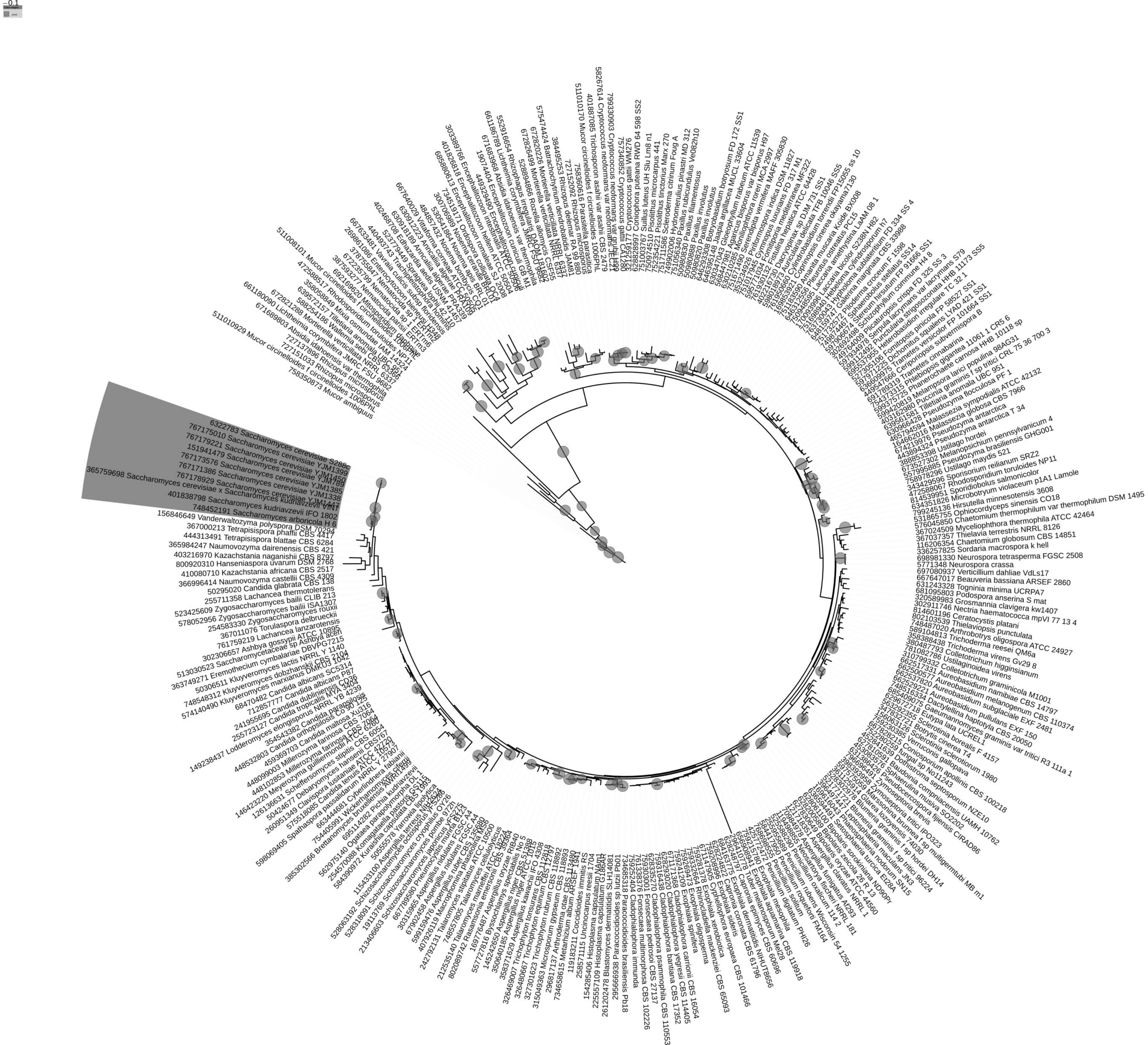

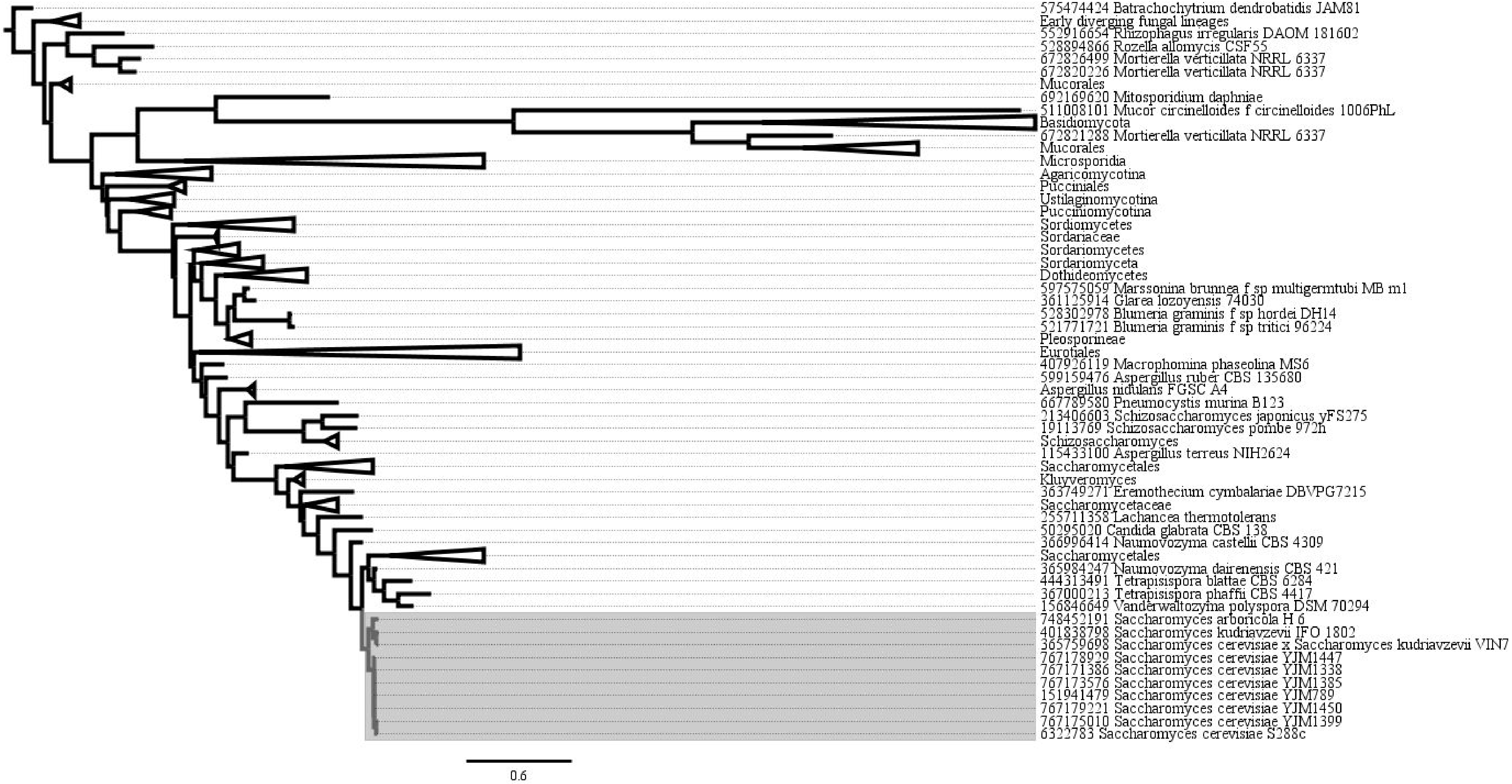
575474424 Batrachochytrium dendrobatidis JAM81
Early diverging fungal lineages
552916654 Rhizophagus irregularis DAOM 181602
528894866 Rozella allomycis CSF55
672826499 Mortierella verticillata NRRL 6337
672820226 Mortierella verticillata NRRL 6337
Mucorales
692169620 Mitosporidium daphniae
511008101 Mucor circinelloides f circinelloides 1006PhL
Basidiomycota
672821288 Mortierella verticillata NRRL 6337
Mucorales
Microsporidia
Agaricomycotina
Pucciniales
Ustilaginomycotina
Pucciniomycotina
Sordiomycetes
Sordariaceae
Sordariomycetes
Sordariomyceta
Dothideomycetes
597575059 Marssonina brunnea f sp multigermtubi MB m1
361125914 Glarea lozoyensis 74030
528302978 Blumeria graminis f sp hordei DH14
521771721 Blumeria graminis f sp tritici 96224
Pleosporineae
Eurotiales
407926119 Macrophomina phaseolina MS6
599159476 Aspergillus ruber CBS 135680
Aspergillus nidulans FGSC A4
667789580 Pneumocystis murina B123
213406603 Schizosaccharomyces japonicus yFS275
19113769 Schizosaccharomyces pombe 972h
Schizosaccharomyces
115433100 Aspergillus terreus NIH2624
Saccharomycetales
Kluyveromyces
363749271 Eremothecium cymbalariae DBVPG7215
Saccharomycetaceae
255711358 Lachancea thermotolerans
50295020 Candida glabrata CBS 138
366996414 Naumovozyma castellii CBS 4309
Saccharomycetales
365984247 Naumovozyma dairenensis CBS 421
444313491 Tetrapisispora blattae CBS 6284
367000213 Tetrapisispora phaffii CBS 4417
156846649 Vanderwaltozyma polyspora DSM 70294
748452191 Saccharomyces arboricola H 6
401838798 Saccharomyces kudriavzevii IFO 1802
365759698 Saccharomyces cerevisiae x Saccharomyces kudriavzevii VIN7
767178929 Saccharomyces cerevisiae YJM1447
767171386 Saccharomyces cerevisiae YJM1338
767173576 Saccharomyces cerevisiae YJM1385
151941479 Saccharomyces cerevisiae YJM789
767179221 Saccharomyces cerevisiae YJM1450
767175010 Saccharomyces cerevisiae YJM1399
6322783 Saccharomyces cerevisiae S288c
0.6

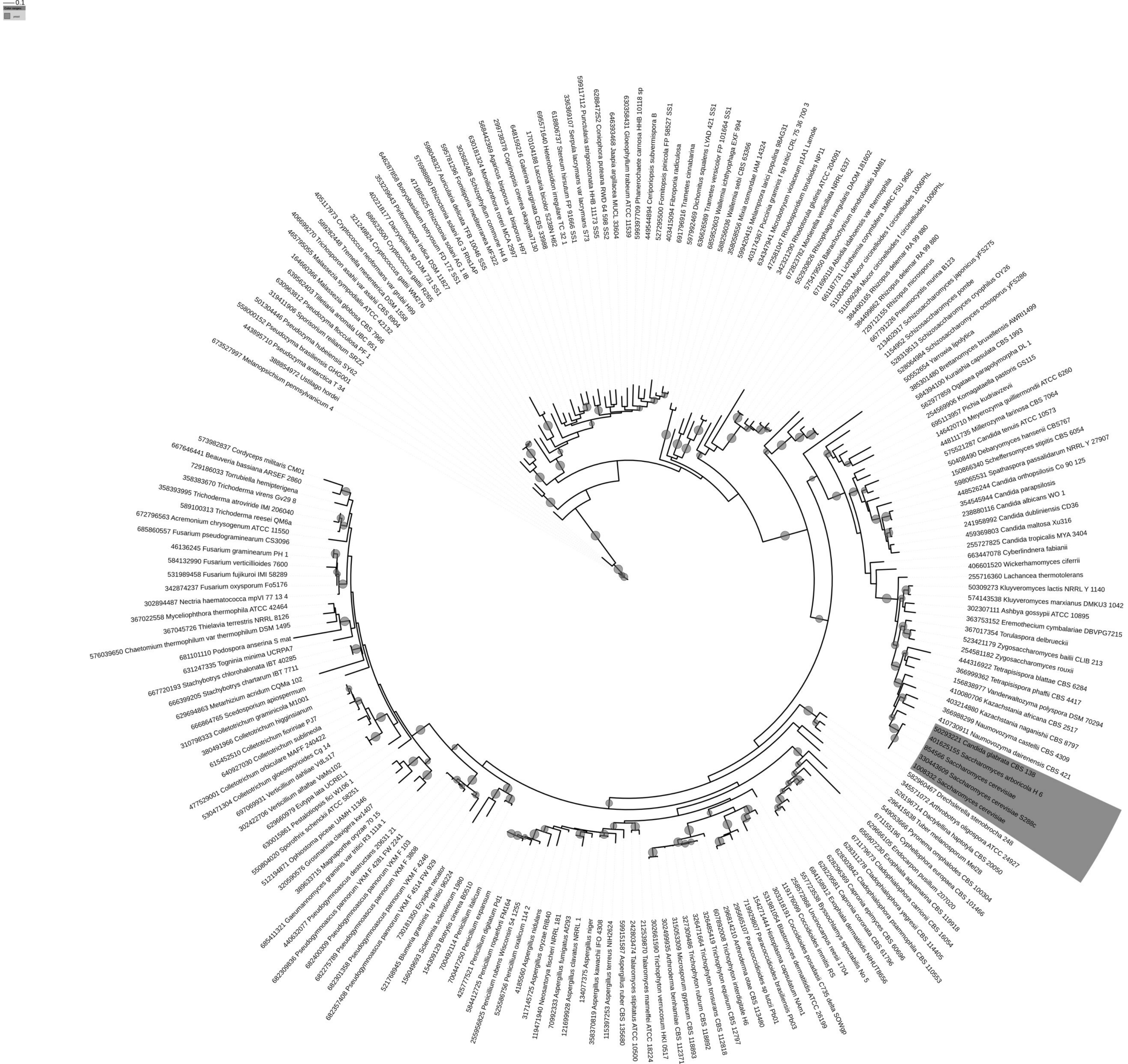
0.1
Color ranges
yeast
573982837 Cordyceps militaris CM01
667646441 Beauveria bassiana ARSEF 2860
729186033 Torrubiella hemipterigena
358383670 Trichoderma virens Gv29 8
358393995 Trichoderma atroviride IMI 206040
589100313 Trichoderma reesei QM6a
672796563 Acremonium chrysogenum ATCC 11550
685860557 Fusarium pseudograminearum CS3096
46136245 Fusarium graminearum PH 1
584132990 Fusarium verticillioides 7600
531989458 Fusarium fujikuroi IMI 58289
342874237 Fusarium oxysporum Fo5176
302894487 Nectria haematococca mpVI 77 13 4
367022558 Myceliophthora thermophila ATCC 42464
367045726 Thielavia terrestris NRRL 8126
576039650 Chaetomium thermophilum var thermophilum DSM 1495
681101110 Podospora anserina S mat
631247335 Togninia minima UCRPA7
667720193 Stachybotrys chlorohalonata IBT 40285
666399205 Stachybotrys chartarum IBT 7711
629694863 Metarhizium acridum CQMa 102
666864765 Scedosporium apiospermum
310798333 Colletotrichum graminicola M1001
380491966 Colletotrichum higginsianum
615452510 Colletotrichum fioriniae PJ7
640927030 Colletotrichum sublineola
477529001 Colletotrichum orbiculare MAFF 240422
530471304 Colletotrichum gloeosporioides Cg 14
697069931 Verticillium dahliae VdLs17
302422706 Verticillium alfalfae VaMs102
629660979 Eutypa lata UCREL1
630015861 Pestalotiopsis fici W106 1
550804020 Sporothrix schenckii ATCC 58251
512194871 Ophiostoma piceae UAMH 11346
320590576 Grosmannia clavigera kw1407
389633715 Magnaporthe oryzae 70 15
685411321 Gaeumannomyces graminis var tritici R3 111a 1
440632077 Pseudogymnoascus destructans 20631 21
682309836 Pseudogymnoascus pannorum VKM F 4281 FW 2241
682400309 Pseudogymnoascus pannorum VKM F 3808
682275789 Pseudogymnoascus pannorum VKM F 103
682301358 Pseudogymnoascus pannorum VKM F 4246
682357408 Pseudogymnoascus pannorum VKM F 4514 FW 929
521768945 Blumeria graminis f sp tritici 96224
730181350 Erysiphe necator
156049693 Sclerotinia sclerotiorum 1980
154309129 Botrytis cinerea B05 10
700492114 Penicillium italicum
700477250 Penicillium expansum
425777521 Penicillium digitatum Pd1
584412725 Penicillium roqueforti FM164
255956825 Penicillium rubens Wisconsin 54 1255
525586756 Penicillium oxalicum 114 2
4185560 Aspergillus nidulans
317145725 Aspergillus oryzae RIB40
119471940 Neosartorya fischeri NRRL 181
70992333 Aspergillus fumigatus Af293
121699928 Aspergillus clavatus NRRL 1
134077375 Aspergillus niger
358370819 Aspergillus kawachii IFO 4308
115397253 Aspergillus terreus NIH2624
599151587 Aspergillus ruber CBS 135680
242803474 Talaromyces stipitatus ATCC 10500
212539870 Talaromyces marneffei ATCC 18224
302661590 Trichophyton verrucosum HKI 0517
302499935 Arthroderma benhamiae CBS 112371
315053309 Microsporum gypseum CBS 118893
327309486 Trichophyton rubrum CBS 118892
326471664 Trichophyton tonsurans CBS 112818
326485419 Trichophyton equinum CBS 127 97
607882008 Trichophyton interdigitale H6
296814210 Arthroderma otae CBS 113480
295665107 Paracoccidioides brasiliensis Pb03
719928087 Paracoccidioides sp lutzi Pb01
154271444 Histoplasma capsulatum NAm1
531981054 Blastomyces dermatitidis ATCC 26199
303318191 Coccidioides immitis RS
119176099 Coccidioides posadasii C735 delta SOWgp
258572868 Uncinocarpus reesii 1704
557723538 Byssochlamys spectabilis No 5
684158912 Exophiala dermatitidis NIH/UT8656
628289581 Capronia epimyces CBS 606 96
628296369 Capronia coronata CBS 617 96
628383842 Cladophialophora psammophila CBS 110553
628311278 Cladophialophora yegresii CBS 114405
671179673 Cladophialophora carrionii CBS 160 54
656907230 Exophiala aquamarina CBS 119918
629666105 Endocarpon pusillum Z07020
671155196 Cyphellophora europaea CBS 101466
549053666 Pyronema omphalodes CBS 100304
296415638 Tuber melanosporum Mel28
526196714 Dactylellina haptotyla CBS 200 50
345571072 Arthrobotrys oligospora ATCC 24927
582960467 Drechslerella stenobrocha 248
1008332 Saccharomyces cerevisiae
330443609 Saccharomyces cerevisiae S288c
854566 Saccharomyces cerevisiae
401625155 Saccharomyces arboricola H 6
50293221 Candida glabrata CBS 138
410730911 Naumovozyma dairenensis CBS 421
366988299 Naumovozyma castellii CBS 4309
403214880 Kazachstania naganishii CBS 8797
410080706 Kazachstania africana CBS 2517
156838977 Vanderwaltozyma polyspora DSM 70294
366999362 Tetrapisispora phaffii CBS 4417
444316922 Tetrapisispora blattae CBS 6284
254581182 Zygosaccharomyces rouxii
523421179 Zygosaccharomyces bailii CLIB 213
367017354 Torulaspora delbrueckii
363753152 Eremothecium cymbalariae DBVPG7215
302307111 Ashbya gossypii ATCC 10895
574143538 Kluyveromyces marxianus DMKU3 1042
50309273 Kluyveromyces lactis NRRL Y 1140
255716360 Lachancea thermotolerans
406601520 Wickerhamomyces ciferrii
663447078 Cyberlindnera fabianii
255727825 Candida tropicalis MYA 3404
459369803 Candida maltosa Xu316
241958992 Candida dubliniensis CD36
238880116 Candida albicans WO 1
354545944 Candida parapsilosis
448526244 Candida orthopsilosis Co 90 125
598065531 Spathaspora passalidarum NRRL Y 27907
150866340 Scheffersomyces stipitis CBS 6054
50408490 Debaryomyces hansenii CBS767
575521287 Candida tenuis ATCC 10573
448111735 Millerozyma farinosa CBS 7064
146420710 Meyerozyma guilliermondii ATCC 6260
695113957 Pichia kudriavzevii
254569906 Komagataella pastoris GS115
562977859 Ogataea parapolymorpha DL 1
584394100 Kuraishia capsulata CBS 1993
385301480 Brettanomyces bruxellensis AWRI1499
528064984 Yarrowia lipolytica
50552654 Schizosaccharomyces octosporus yFS286
528319513 Schizosaccharomyces cryophilus OY26
1154952 Schizosaccharomyces pombe
213402917 Schizosaccharomyces japonicus yFS275
729712155 Pneumocystis murina B123
667791225 Rhizopus microsporus
384498862 Rhizopus delemar RA 99 880
384490165 Rhizopus delemar RA 99 880
511009296 Mucor circinelloides f circinelloides 1006PhL
511004333 Mucor circinelloides f circinelloides 1006PhL
661187731 Lichtheimia corymbifera JMRC FSU 9682
671690118 Absidia idahoensis var thermophila
575479550 Batrachochytrium dendrobatidis JAM81
552938826 Rhizophagus irregularis DAOM 181602
672823782 Mortierella verticillata NRRL 6337
342321290 Rhodotorula glutinis ATCC 204091
472581047 Rhodosporidium toruloides NP11
634347941 Microbotryum violaceum p1A1 Lamole
403174367 Puccinia graminis f sp tritici CRL 75 36 700 3
599420415 Melampsora larici populina 98AG31
358058856 Mixia osmundae IAM 14324
588256036 Wallemia sebi CBS 633 66
685952603 Wallemia ichthyophaga EXF 994
636626589 Trametes versicolor FP 101664 SS1
597992469 Dichomitus squalens LYAD 421 SS1
691796916 Trametes cinnabarina
403415094 Fibroporia radiculosa
527295500 Fomitopsis pinicola FP 58527 SS1
449544894 Ceriporiopsis subvermispora B
569397709 Phanerochaete carnosa HHB 10118 sp
630358431 Gloeophyllum trabeum ATCC 11539
646393468 Jaapia argillacea MUCL 33604
628847252 Coniophora puteana RWD 64 598 SS2
599117112 Punctularia strigosozonata HHB 11173 SS5
336369107 Serpula lacrymans var lacrymans S7 3
618806737 Stereum hirsutum FP 91666 SS1
695571640 Heterobasidion irregulare TC 32 1
170104188 Laccaria bicolor S238N H82
648159216 Galerina marginata CBS 339 88
299738378 Coprinopsis cinerea okayama7130
568442369 Agaricus bisporus var bisporus H97
630181324 Moniliophthora roreri MCA 2997
302682408 Schizophyllum commune H4 8
595781296 Fomitiporia mediterranea MF3 22
598048327 Auricularia delicata TFB 10046 SS5
576986990 Rhizoctonia solani AG 3 Rhs1AP
471885625 Rhizoctonia solani AG 1 IB
646287858 Botryobasidium botryosum FD 172 SS1
353239643 Piriformospora indica DSM 11827
402218177 Dacryopinax sp DJM 731 SS1
686622500 Cryptococcus gattii R265
321249824 Cryptococcus gattii WM276
405117973 Cryptococcus neoformans var grubii H99
589282448 Tremella mesenterica DSM 1558
406699270 Trichosporon asahii var asahii CBS 8904
465795055 Malassezia sympodialis ATCC 42132
164660366 Malassezia globosa CBS 7966
639562403 Tilletiaria anomala UBC 951
630963812 Pseudozyma flocculosa PF 1
319411906 Sporisorium reilianum SRZ2
501304446 Pseudozyma hubeiensis SY62
558000152 Pseudozyma brasiliensis GHG001
443895710 Pseudozyma antarctica T 34
388854972 Ustilago hordei
673527997 Melanopsichium pennsylvanicum 4

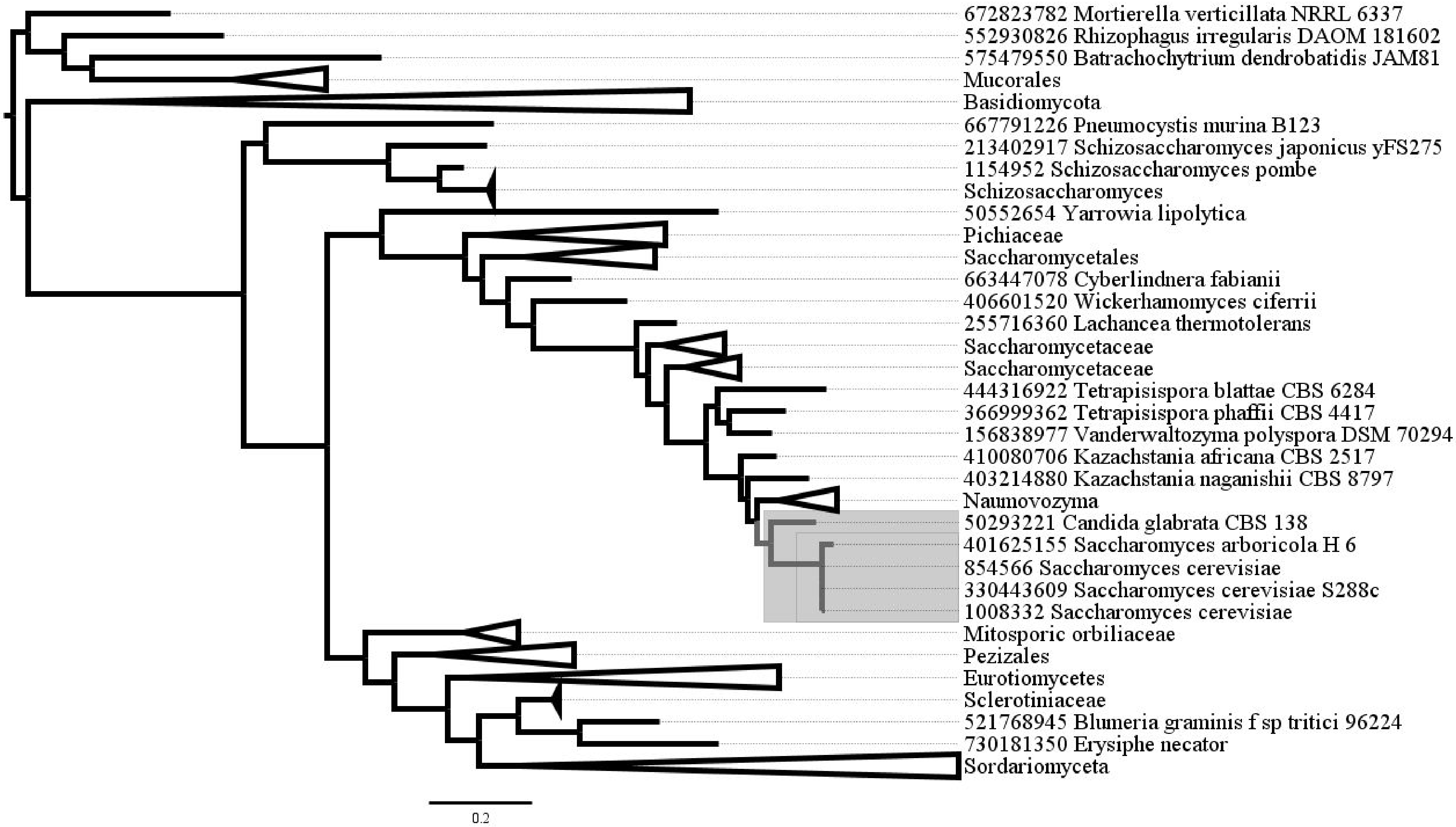
672823782 Mortierella verticillata NRRL 6337
552930826 Rhizophagus irregularis DAOM 181602
575479550 Batrachochytrium dendrobatidis JAM81
Mucorales
Basidiomycota
667791226 Pneumocystis murina B123
213402917 Schizosaccharomyces japonicus yFS275
1154952 Schizosaccharomyces pombe
Schizosaccharomyces
50552654 Yarrowia lipolytica
Pichiaceae
Saccharomycetales
663447078 Cyberlindnera fabianii
406601520 Wickerhamomyces ciferrii
255716360 Lachancea thermotolerans
Saccharomycetaceae
Saccharomycetaceae
444316922 Tetrapisispora blattae CBS 6284
366999362 Tetrapisispora phaffii CBS 4417
156838977 Vanderwaltozyma polyspora DSM 70294
410080706 Kazachstania africana CBS 2517
403214880 Kazachstania naganishii CBS 8797
Naumovozyma
50293221 Candida glabrata CBS 138
401625155 Saccharomyces arboricola H 6
854566 Saccharomyces cerevisiae
330443609 Saccharomyces cerevisiae S288c
1008332 Saccharomyces cerevisiae
Mitosporic orbiliaceae
Pezizales
Eurotiomycetes
Sclerotiniaceae
521768945 Blumeria graminis f sp tritici 96224
730181350 Erysiphe necator
Sordariomyceta
0.2

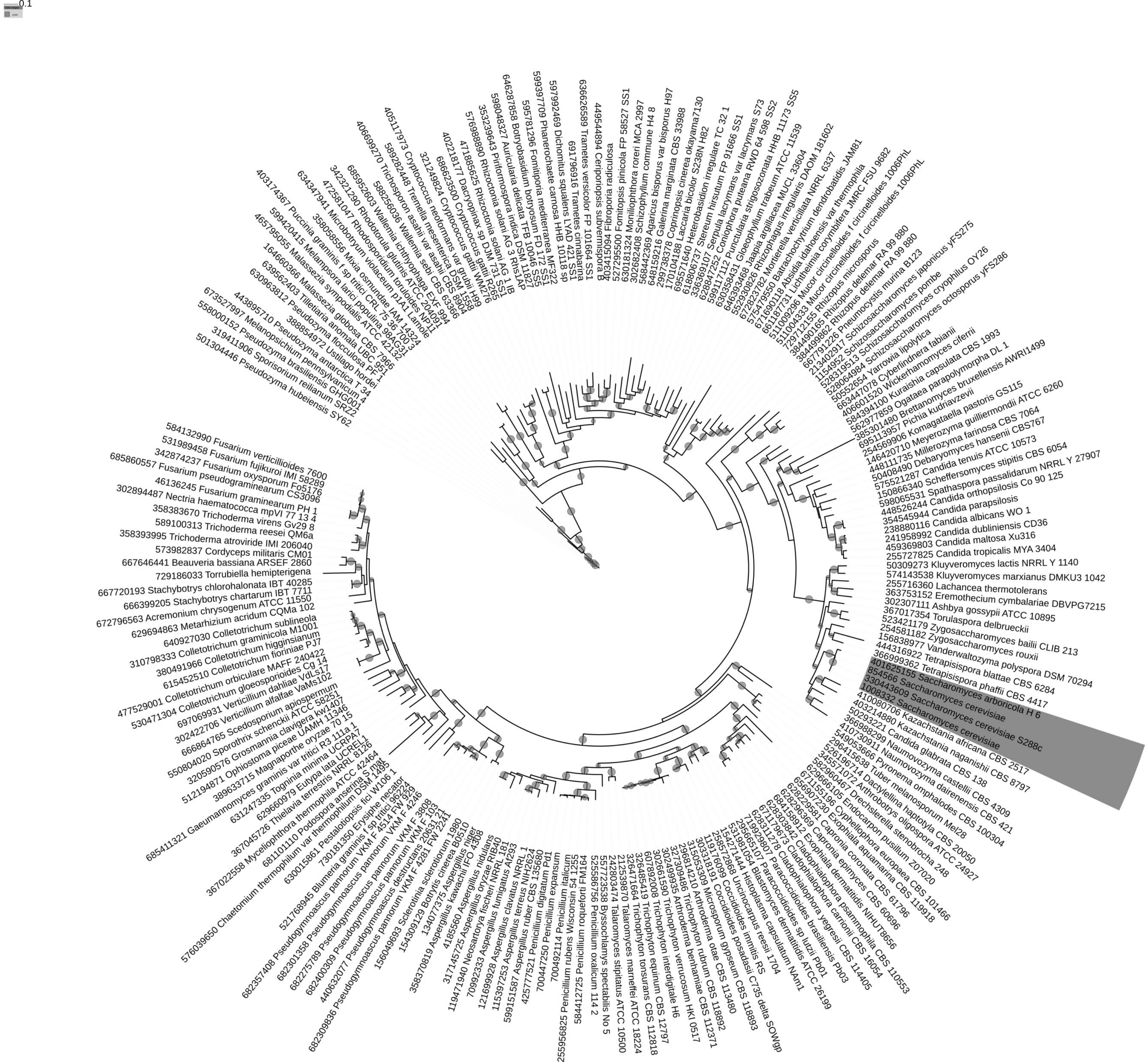
0.1
501304446 Pseudozyma hubeiensis SY62
319411906 Sporisorium reilianum SRZ2
558000152 Pseudozyma brasiliensis GHG001
673527997 Melanopsichium pennsylvanicum 4
443895710 Pseudozyma antarctica T 34
388854972 Ustilago hordei
630963812 Pseudozyma flocculosa PF 1
639562403 Tilletiaria anomala UBC 951
164660366 Malassezia globosa CBS 7966
465795055 Malassezia sympodialis ATCC 42132
599420415 Melampsora larici populina 98AG31
403174367 Puccinia graminis f sp tritici CRL 75 36 700 3
634347941 Microbotryum violaceum p1A1 Lamole
358058556 Mixia osmundae IAM 14324
472581047 Rhodosporidium toruloides NP11
342321290 Rhodotorula glutinis ATCC 204091
685952603 Wallemia ichthyophaga EXF 994
588256036 Wallemia sebi CBS 633 66
406699270 Trichosporon asahii var asahii CBS 8904
599282448 Tremella mesenterica DSM 1558
405117973 Cryptococcus neoformans var grubii H99
321249824 Cryptococcus gattii WM276
686623500 Cryptococcus gattii R265
402218177 Dacryopinax sp DJM 731 SS1
471885625 Rhizoctonia solani AG 1 IB
576988890 Rhizoctonia solani AG 3 Rhs1AP
353239643 Piriformospora indica DSM 11827
598048327 Auricularia delicata TFB 10046 SS5
646287858 Botryobasidium botryosum FD 172 SS1
595781296 Fomitiporia mediterranea MF322
599397709 Phanerochaete carnosa HHB 10118 sp
597992469 Dichomitus squalens LYAD 421 SS1
636626589 Trametes versicolor FP 101664 SS1
691796916 Trametes cinnabarina
449544894 Ceriporiopsis subvermispora B
403415094 Fibroporia radiculosa
527295500 Fomitopsis pinicola FP 58527 SS1
630181324 Moniliophthora roreri MCA 2997
302682408 Schizophyllum commune H4 8
568442369 Agaricus bisporus var bisporus H97
648159216 Galerina marginata CBS 33988
299738378 Coprinopsis cinerea okayama7130
170104188 Laccaria bicolor S238N H82
695571640 Heterobasidion irregulare TC 32 1
618806737 Stereum hirsutum FP 91666 SS1
336369107 Serpula lacrymans var lacrymans S7 3
628847252 Coniophora puteana RWD 64 598 SS2
599117112 Punctularia strigosozonata HHB 11173 SS5
630358431 Gloeophyllum trabeum ATCC 11539
646393468 Jaapia argillacea MUCL 33604
552930826 Rhizophagus irregularis DAOM 181602
672823782 Mortierella verticillata NRRL 6337
575479550 Batrachochytrium dendrobatidis JAM81
676190118 Absidia idahoensis var thermophila
661187731 Lichtheimia corymbifera JMRC FSU 9682
511009296 Mucor circinelloides f circinelloides 1006PhL
511004333 Mucor circinelloides f circinelloides 1006PhL
729712155 Rhizopus microsporus
384490165 Rhizopus delemar RA 99 880
384499862 Rhizopus delemar RA 99 880
667791226 Pneumocystis murina B123
213402917 Schizosaccharomyces japonicus yFS275
1154952 Schizosaccharomyces pombe
528319513 Schizosaccharomyces cryophilus OY26
528064984 Schizosaccharomyces octosporus yFS286
50552654 Yarrowia lipolytica
663447078 Cyberlindnera fabianii
406601520 Wickerhamomyces ciferrii
584394100 Kuraishia capsulata CBS 1993
562977859 Ogataea parapolymorpha DL 1
385301480 Brettanomyces bruxellensis AWRI1499
695113957 Pichia kudriavzevii
254569906 Komagataella pastoris GS115
146420710 Meyerozyma guilliermondii ATCC 6260
448111735 Millerozyma farinosa CBS 7064
50408490 Debaryomyces hansenii CBS767
575521287 Candida tenuis ATCC 10573
150866340 Scheffersomyces stipitis CBS 6054
598065531 Spathaspora passalidarum NRRL Y 27907
448526244 Candida orthopsilosis Co 90 125
354545944 Candida parapsilosis
238880116 Candida albicans WO 1
241958992 Candida dubliniensis CD36
459369803 Candida maltosa Xu316
255727825 Candida tropicalis MYA 3404
50309273 Kluyveromyces lactis NRRL Y 1140
574143538 Kluyveromyces marxianus DMKU3 1042
255716360 Lachancea thermotolerans
363753152 Eremothecium cymbalariae DBVPG7215
302307111 Ashbya gossypii ATCC 10895
367017354 Torulaspora delbrueckii
523421179 Zygosaccharomyces bailii CLIB 213
254581182 Zygosaccharomyces rouxii
156838977 Vanderwaltozyma polyspora DSM 70294
444316922 Tetrapisispora blattae CBS 6284
366999362 Tetrapisispora phaffii CBS 4417
401625155 Saccharomyces arboricola H 6
854566 Saccharomyces cerevisiae
330443609 Saccharomyces cerevisiae S288c
1008332 Saccharomyces cerevisiae
410080706 Kazachstania africana CBS 2517
403214880 Kazachstania naganishii CBS 8797
50293221 Candida glabrata CBS 138
366988299 Naumovozyma castellii CBS 4309
410730911 Naumovozyma dairenensis CBS 421
549053666 Pyronema omphalodes CBS 100304
296415638 Tuber melanosporum Mel28
526196714 Dactylellina haptotyla CBS 20050
345571072 Arthrobotrys oligospora ATCC 24927
582960467 Drechslerella stenobrocha 248
629666105 Endocarpon pusillum Z07020
671155196 Cyphellophora europaea CBS 101466
656907230 Exophiala aquamarina CBS 119918
628229581 Capronia coronata CBS 61796
628296369 Capronia epimyces CBS 60696
684158912 Exophiala dermatitidis NIHUT8656
628303842 Cladophialophora psammophila CBS 110553
671179673 Cladophialophora carrionii CBS 16054
628311278 Cladophialophora yegresii CBS 114405
719929807 Paracoccidioides brasiliensis Pb03
295665107 Paracoccidioides sp lutzii Pb01
531981054 Blastomyces dermatitidis ATCC 26199
154271444 Histoplasma capsulatum NAm1
258572868 Uncinocarpus reesii 1704
119176099 Coccidioides immitis RS
303318191 Coccidioides posadasii C735 delta SOWgp
315053309 Microsporum gypseum CBS 118893
296814210 Arthroderma otae CBS 113480
327309486 Trichophyton rubrum CBS 118892
302499935 Arthroderma benhamiae CBS 112371
302661590 Trichophyton verrucosum HKI 0517
607892008 Trichophyton interdigitale H6
326485419 Trichophyton equinum CBS 12797
326471664 Trichophyton tonsurans CBS 112818
212539870 Talaromyces marneffei ATCC 18224
242803474 Talaromyces stipitatus ATCC 10500
557723538 Byssochlamys spectabilis No 5
525586756 Penicillium oxalicum 114 2
584412725 Penicillium roqueforti FM164
255956825 Penicillium rubens Wisconsin 54 1255
700492114 Penicillium italicum
700447250 Penicillium expansum
425777521 Penicillium digitatum Pd1
599151587 Aspergillus ruber CBS 135680
115397253 Aspergillus terreus NIH2624
121699928 Aspergillus clavatus NRRL 1
70992333 Aspergillus fumigatus Af293
119471940 Neosartorya fischeri NRRL 181
317145725 Aspergillus oryzae RIB40
4185560 Aspergillus nidulans
358370819 Aspergillus kawachii IFO 4308
134077375 Aspergillus niger
154309129 Botrytis cinerea B0510
156049693 Sclerotinia sclerotiorum 1980
682309836 Pseudogymnoascus pannorum VKM F 4281 FW 2241
440632077 Pseudogymnoascus destructans 20631 21
682400309 Pseudogymnoascus pannorum VKM F 103
682275789 Pseudogymnoascus pannorum VKM F 3808
682301358 Pseudogymnoascus pannorum VKM F 4246
682357408 Pseudogymnoascus pannorum VKM F 4514 FW 929
521768945 Blumeria graminis f sp tritici 96224
730181350 Erysiphe necator
630015861 Pestalotiopsis fici W106 1
576039650 Chaetomium thermophilum var thermophilum DSM 1495
681101110 Podospora anserina S mat
367022558 Myceliophthora thermophila ATCC 42464
367045726 Thielavia terrestris NRRL 8126
629660979 Eutypa lata UCREL1
631247335 Togninia minima UCRPA7
685411321 Gaeumannomyces graminis var tritici R3 111a 1
389633715 Magnaporthe oryzae 70 15
512194871 Ophiostoma piceae UAMH 11346
320590576 Grosmannia clavigera kw1407
550804020 Sporothrix schenckii ATCC 58251
666864765 Scedosporium apiospermum
302422706 Verticillium alfalfae VaMs102
697069931 Verticillium dahliae VdLs17
530471304 Colletotrichum gloeosporioides Cg 14
477529001 Colletotrichum orbiculare MAFF 240422
615452510 Colletotrichum fioriniae PJ7
380491966 Colletotrichum higginsianum
310798333 Colletotrichum graminicola M1001
640927030 Colletotrichum sublineola
629694863 Metarhizium acridum CQMa 102
672796563 Acremonium chrysogenum ATCC 11550
666399205 Stachybotrys chartarum IBT 7711
667720193 Stachybotrys chlorohalonata IBT 40285
729186033 Torrubiella hemipterigena
667646441 Beauveria bassiana ARSEF 2860
573982837 Cordyceps militaris CM01
358393995 Trichoderma atroviride IMI 206040
589100313 Trichoderma reesei QM6a
358383670 Trichoderma virens Gv29 8
302894487 Nectria haematococca mpVI 77 13 4
46136245 Fusarium graminearum PH 1
685860557 Fusarium pseudograminearum CS3096
342874237 Fusarium oxysporum Fo5176
531989458 Fusarium fujikuroi IMI 58289
584132990 Fusarium verticillioides 7600

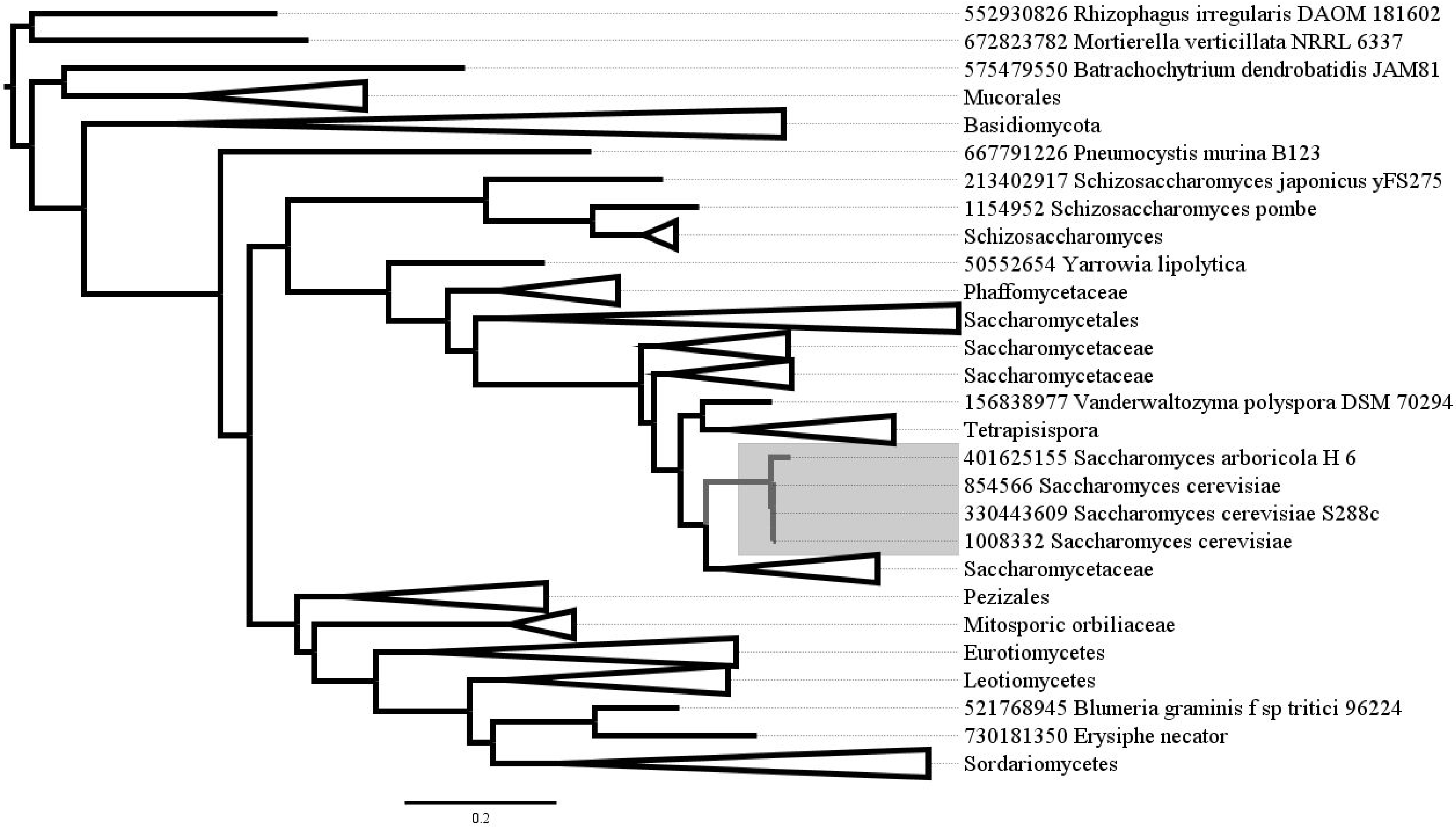
552930826 Rhizophagus irregularis DAOM 181602
672823782 Mortierella verticillata NRRL 6337
575479550 Batrachochytrium dendrobatidis JAM81
Mucorales
Basidiomycota
667791226 Pneumocystis murina B123
213402917 Schizosaccharomyces japonicus yFS275
1154952 Schizosaccharomyces pombe
Schizosaccharomyces
50552654 Yarrowia lipolytica
Phaffomycetaceae
Saccharomycetales
Saccharomycetaceae
Saccharomycetaceae
156838977 Vanderwaltozyma polyspora DSM 70294
Tetrapisispora
401625155 Saccharomyces arboricola H 6
854566 Saccharomyces cerevisiae
330443609 Saccharomyces cerevisiae S288c
1008332 Saccharomyces cerevisiae
Saccharomycetaceae
Pezizales
Mitosporic orbiliaceae
Eurotiomycetes
Leotiomycetes
521768945 Blumeria graminis f sp tritici 96224
730181350 Erysiphe necator
Sordariomycetes
0.2

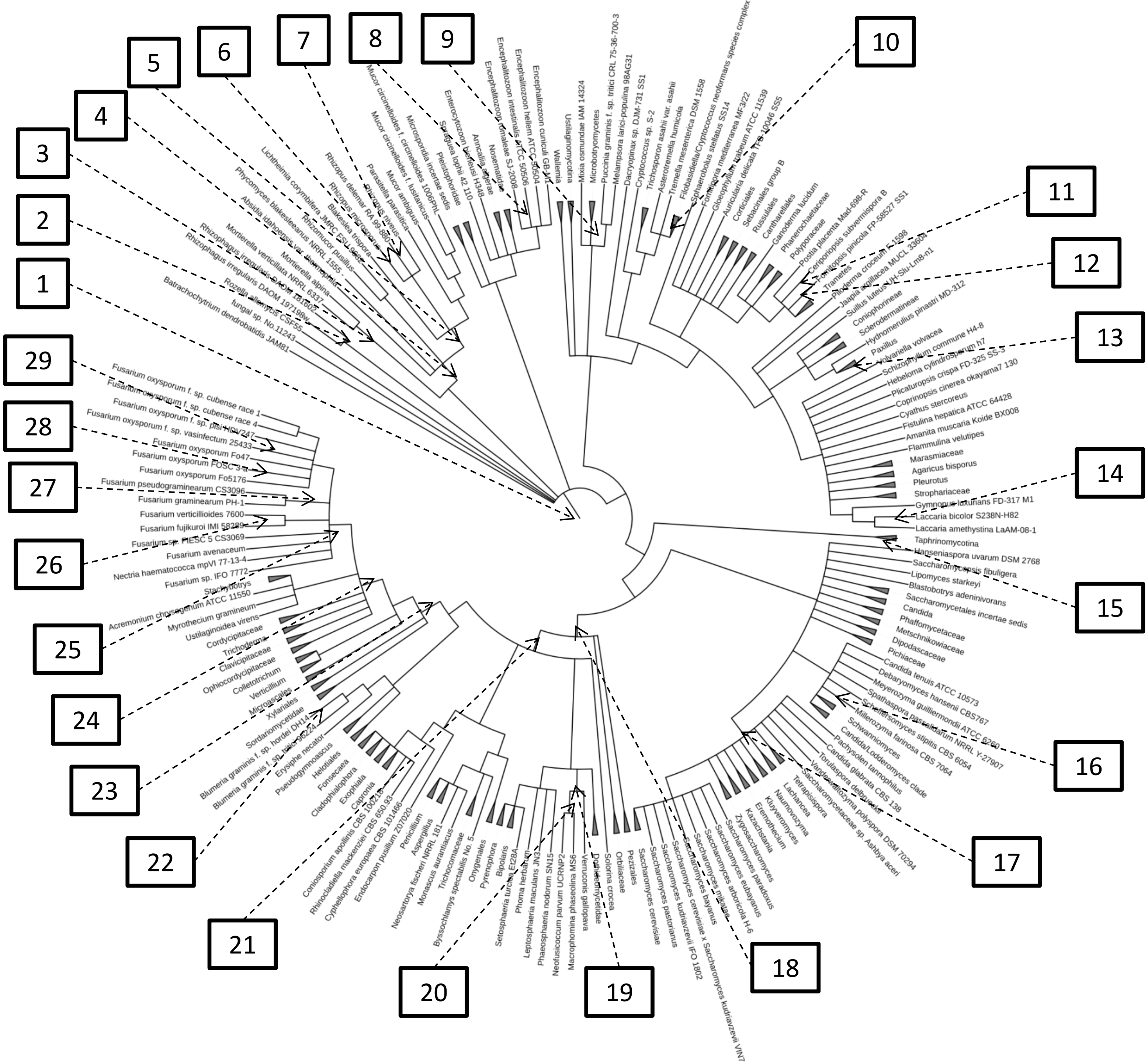
1
2
3
4
5
6
7
8
9
10
11
12
13
14
15
16
17
18
19
20
21
22
23
24
25
26
27
28
29
Batrachochytrium dendrobatidis JAM81
fungal sp. No.11243
Rozella allomycis CSF55
Rhizophagus irregularis DAOM 197198w
Rhizophagus irregularis DAOM 181602
Mortierella verticillata NRRL 6337
Mortierella alpina
Absidia idahoensis var. thermophila
Phycomyces blakesleeanus NRRL 1555
Lichtheimia corymbifera JMRC FSU 9682
Rhizomucor pusillus
Blakeslea trispora
Rhizopus microsporus
Rhizopus delemar RA 99-880
Rhizopus niveus
Parasitella parasitica
Mucor ambiguus
Mucor circinelloides 1006PhL
Mucor circinelloides f. lusitanicus
Mucor circinelloides f. circinelloides 1006PhL
Microsporidia incertae sedis
Pleistophoridae
Spraguea lophii 42 110
Enterocytozoon bieneusi H348
Amradillia algerae
Nosematidae
Encephalitozoon romaleae SJ-2008
Encephalitozoon intestinalis ATCC 50506
Encephalitozoon hellem ATCC 50504
Encephalitozoon cuniculi GB-M1
Wallemia
Ustilaginomycotina
Mixia osmundae IAM 14324
Microbotryomycetes
Puccinia graminis f. sp. tritici CRL 75-36-700-3
Melampsora larici-populina 98AG31
Dacryopinax sp. DJM-731 SS1
Cryptococcus sp. S-2
Trichosporon asahii var. asahii
Asterotremella humicola
Tremella mesenterica DSM 1558
Filobasidiella/Cryptococcus neoformans species complex
Sphaerobolus stellatus SS14
Fomitiporia mediterranea MF3/22
Gloeophyllum trabeum ATCC 11539
Auricularia delicata TFB-10046 SS5
Corticiales
Sebacinales group B
Russulales
Cantharellales
Ganoderma lucidum
Phanerochaetaceae
Polyporaceae
Postia placenta Mad-698-R
Ceriporiopsis subvermispora B
Fomitopsis pinicola FP-58527 SS1
Trametes
Piloderma croceum F 1598
Jaapia argillacea MUCL 33604
Suillus luteus UH-Slu-Lm8-n1
Coniophorineae
Sclerodermatineae
Hydnomerulius pinastri MD-312
Paxillus
Volvariella volvacea
Schizophyllum commune H4-8
Hebeloma cylindrosporum h7
Plicaturopsis crispa FD-325 SS-3
Coprinopsis cinerea okayama7 130
Cyathus stercoreus
Fistulina hepatica ATCC 64428
Amanita muscaria Koide BX008
Flammulina velutipes
Marasmiaceae
Agaricus bisporus
Pleurotus
Strophariaceae
Gymnopus luxurians FD-317 M1
Laccaria bicolor S238N-H82
Laccaria amethystina LaAM-08-1
Taphrinomycotina
Hanseniaspora uvarum DSM 2768
Saccharomycopsis fibuligera
Lipomyces starkeyi
Blastobotrys adeninivorans
Saccharomycetales incertae sedis
Candida
Phaffomycetaceae
Metschnikowiaceae
Dipodascaceae
Pichiaceae
Candida tenuis ATCC 10573
Debaryomyces hansenii CBS767
Meyerozyma guilliermondii ATCC 6260
Spathaspora passalidarum NRRL Y-27907
Scheffersomyces stipitis CBS 6054
Millerozyma farinosa CBS 7064
Schwanniomyces
Candida/Lodderomyces clade
Pachysolen tannophilus
Candida glabrata CBS 138
Torulaspora delbrueckii
Vanderwaltozyma polyspora DSM 70294
Saccharomycetaceae sp. Ashbya aceri
Tetrapisispora
Lachancea
Naumovozyma
Kluyveromyces
Eremothecium
Kazachstania
Zygosaccharomyces
Saccharomyces paradoxus
Saccharomyces eubayanus
Saccharomyces arboricola H-6
Saccharomyces mikatae
Saccharomyces bayanus
Saccharomyces cerevisiae x Saccharomyces kudriavzevii VIN7
Saccharomyces kudriavzevii IFO 1802
Saccharomyces pastorianus
Saccharomyces cerevisiae
Pezizales
Orbiliaceae
Solorina crocea
Dothideomycetidae
Verruconis gallopava
Macrophomina phaseolina MS6
Neofusicoccum parvum UCRNP2
Phaeosphaeria nodorum SN15
Leptosphaeria maculans JN3
Phoma herbarum
Setosphaeria turcica Et28A
Bipolaris
Pyrenophora
Onygenales
Byssochlamys spectabilis No. 5
Trichocomaceae
Monascus aurantiacus
Neosartorya fischeri NRRL 181
Aspergillus
Penicillium
Endocarpon pusillum Z07020
Cyphellophora europaea CBS 101466
Rhinocladiella mackenziei CBS 650.93
Coniosporium apollinis CBS 100218
Capronia
Exophiala
Cladophialophora
Fonsecaea
Helotiales
Pseudogymnoascus
Erysiphe necator
Blumeria graminis f. sp. tritici 96224
Blumeria graminis f. sp. hordei DH14
Sordariomycetidae
Xylariales
Microascales
Verticillium
Colletotrichum
Ophiocordycipitaceae
Clavicipitaceae
Trichoderma
Cordycipitaceae
Ustilaginoidea virens
Myrothecium gramineum
Acremonium chrysogenum ATCC 11550
Stachybotrys
Fusarium sp. IFO 7772
Nectria haematococca mpVI 77-13-4
Fusarium avenaceum
Fusarium sp. FIESC 5 CS3069
Fusarium fujikuroi IMI 58289
Fusarium verticillioides 7600
Fusarium graminearum PH-1
Fusarium pseudograminearum CS3096
Fusarium oxysporum Fo5176
Fusarium oxysporum FOSC 3-a
Fusarium oxysporum Fo47
Fusarium oxysporum f. sp. vasinfectum 25433
Fusarium oxysporum f. sp. pisi HDV247
Fusarium oxysporum f. sp. cubense race 4
Fusarium oxysporum f. sp. cubense race 1
